# Nitriles with High Gas-Phase Basicity—Part II Transmission of the Push–Pull Effect through Methylenecyclopropene and Cyclopropenimine Scaffolds Intercalated between Different Electron Donor(s) and the Cyano N-Protonation Site

**DOI:** 10.3390/molecules27144370

**Published:** 2022-07-07

**Authors:** Ewa D. Raczyńska, Jean-François Gal, Pierre-Charles Maria, Ghulam Sakhi Sakhawat, Mohammad Qasem Fahim, Hamid Saeidian

**Affiliations:** 1 Department of Chemistry, Warsaw University of Life Sciences (SGGW), 02-776 Warsaw, Poland; 2 Institut de Chimie de Nice, Université Côte d’Azur, Parc Valrose, 06108 Nice, France; pierre-charles.maria@univ-cotedazur.fr; 3 Department of Chemistry, Science and Research Branch, Islamic Azad University, Tehran P.O. Box 14515-775, Iran; gh.s.sakhawat@gmail.com (G.S.S.); mqasemfahim3@gmail.com (M.Q.F.); 4 Department of Science, Payame Noor University (PNU), Tehran P.O. Box 19395-4697, Iran

**Keywords:** nitrile basicity in the gas phase, methylenecyclopropene and cyclopropenimine scaffolds, geometrical and rotational isomerism, substituent effects, electron delocalization, DFT studies

## Abstract

This work extends our earlier quantum chemical studies on the gas-phase basicity of very strong N-bases to two series of nitriles containing the methylenecyclopropene and cyclopropenimine scaffolds with dissymmetrical substitution by one or two electron-donating substituents such as Me, NR_2_, N=C (NR_2_)_2_, and N=P (NR_2_)_3_, the last three being strong donors. For a proper prediction of their gas-phase base properties, all potential isomeric phenomena and reasonable potential protonation sites are considered to avoid possible inconsistencies when evaluating the energetic parameters and associated protonation or deprotonation equilibria B + H^+^ = BH^+^. More than 250 new isomeric structures for neutral and protonated forms are analyzed. The stable structures are selected and the favored ones identified. The microscopic (kinetic) gas-phase basicity parameters (PA and GB) corresponding to N sites (cyano and imino in the cyclopropenimine or in the substituents) in each isomer are calculated. The macroscopic (thermodynamic) PAs and GBs, referring to the isomeric mixtures of favored isomers, are also estimated. The total (pushing) substituent effects are analyzed for monosubstituted and disubstituted derivatives containing two identical or two different substituents. Electron delocalization is examined in the two π–π conjugated transmitters, the methylenecyclopropene and cyclopropenimine scaffolds. The aromatic character of the three-membered ring is also discussed.

## 1. Introduction

**Chart 1:**
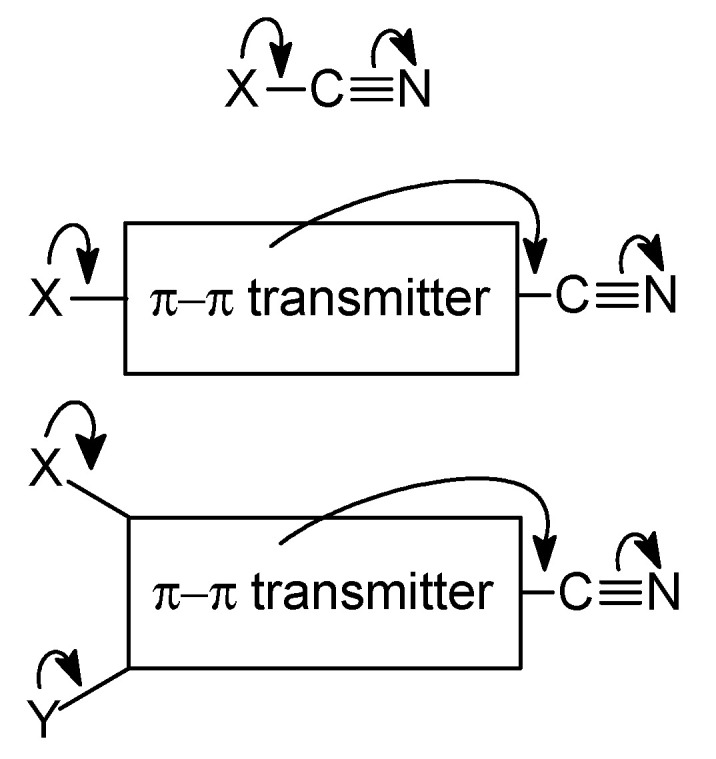
Improved push–pull effect in nitriles when the π transmitter can carry two electron-donor substituents.

The design and synthesis of very strong organic bases is a topic of continuing interest [1,2,3,4,5,6]. Among the various approaches to “superbasicity”, significant basicity increases can be expected using the push–pull concept [2]. The principle of the push–pull effect is to increase the electron density on an electron-pulling (or electron-withdrawing) part of a molecule by adding an electron-pushing (electron-donating) substituent on a scaffold capable of transmitting the resonance effect between the two entities and, at the same time, enhancing the functional group basicity. This approach can increase the basicity of a relatively weak functional group such as nitrile to the rank of a superbase [7,8], particularly when two pushing groups can be attached to a transmitting scaffold (Chart 1) [9].

**Figure 1:**
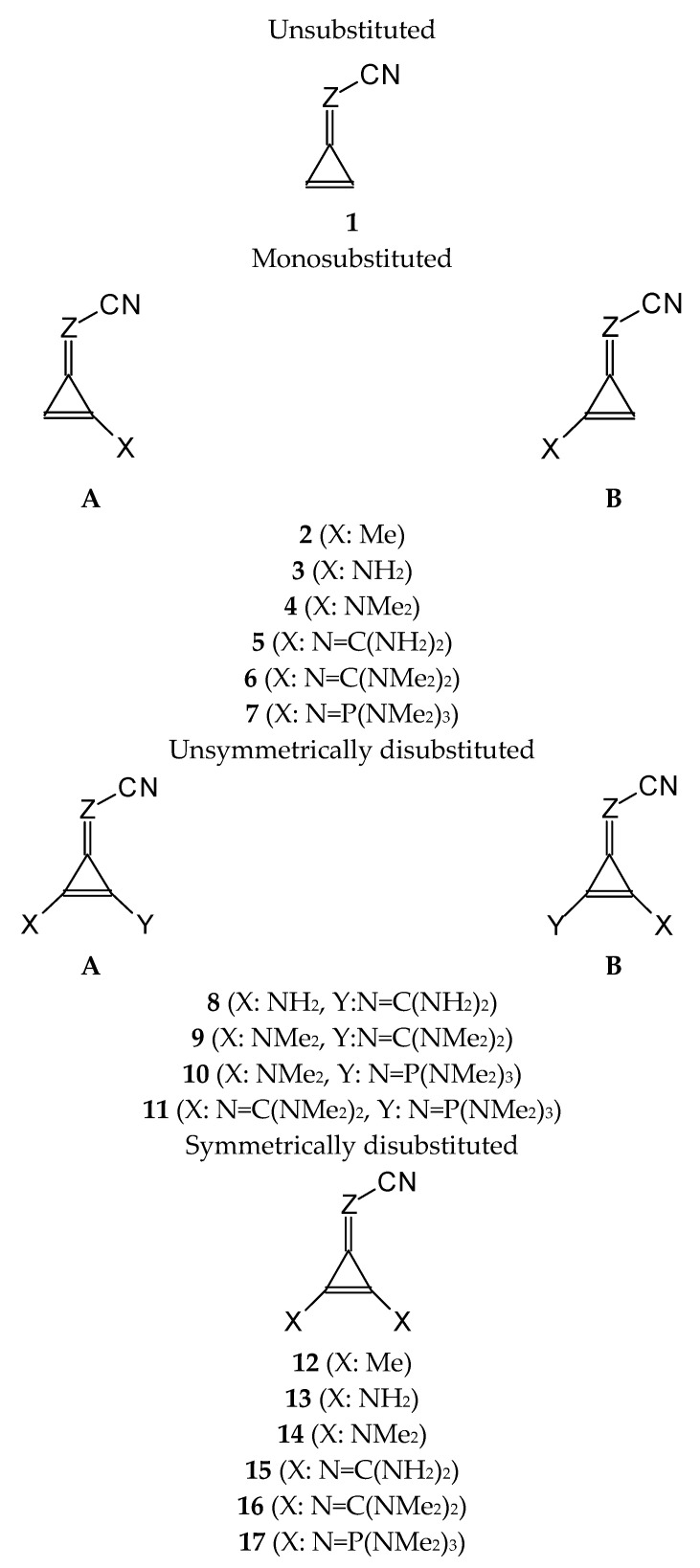
Push–pull nitriles built around the methylenecyclopropene (series **I**, Z = CH) and cyclopropenimine (series **II**, Z = N) scaffolds. Unsubstituted and symmetrically disubstituted systems, studied in [9], were compiled with those investigated in this work.

In Part I of this study [9], we underlined the specificities of the transmitting scaffold constituted by the unsaturated cyclic system made of three sp^2^ carbons with an exocyclic =CH– or =N– bond, namely the methylenecyclopropene and cyclopropenimine scaffolds depicted in Figure 1. The small dimension of the resonance-transmitting system helps reduce the molecule size and molecular weight, which are among the conditions for a practical utilization of superbases [5]. The optimization of the properties of superbasic nitrile systems based on these two scaffolds necessitates the knowledge of the combined effects of the two substituents that can be plugged to the transmitter: the additivity/non-additivity (departure from additivity of the effect of each substituent) of their pushing effects, steric/intramolecular interactions, and geometrical and/or conformational isomerisms. These aspects are studied in this work by comparing results already obtained on symmetrically substituted nitriles [9] with new data obtained on non-symmetrically substituted nitriles.

For our studies, we chose two series of nitriles (**I.2-I.11** and **II.2-II.11**), monosubstituted and unsymmetrically disubstituted by medium (Me) and strong electron donor groups (NR_2_, N=C(NR_2_)_2_, and N=P(NR_2_)_3_), and we compared their structure-basicity results to those found previously for symmetrically disubstituted derivatives (**I.12-I.17** and **II.12-II.17**). To simplify the substituents labelling, in the following the name “simple” is given to the Me and NR_2_ groups, and “large” to the others that contain a π bond.

The Me group (s) in **I.2**, **II.2**, **I.12**, and **II.12** interact with the C≡N group through the methylenecyclopropene and cyclopropenimine fragments mainly by the σ–π hyperconjugation and polarizability effect (s). The amino substituents, NH_2_ and NMe_2_, in **I.3-I.4**, **II.3-II.4**, **I.13-I.14**, and **II.13-II.14** possess a lone (n) electron pair on the amino N atom. By interacting with the pulling C≡N function through the π–π conjugated transmitters, the NH_2_ and NMe_2_ lose some electron density in favor of the cyano N atom.

The effects of strong pushing groups, N=C(NH_2_)_2_, N=C(NMe_2_)_2_, and N=P(NMe_2_)_3_, in **I.5-I.7**, **II.5-II.7**, **I.15-I.17**, and **II.15-II.17** benefit from n-electrons of the amino N atoms according to the cross n–π conjugation. The imino C=N and phosphimino P=N moieties of these groups play the role of transmitters of the labile n-electrons. In unsymmetrically disubstituted derivatives **I.8-I.11** and **II.8-II.11** containing a pair of different substituents (NH_2_ and N=C(NH_2_)_2_, NMe_2_ and N=C(NMe_2_)_2_, NMe_2_ and N=P(NMe_2_)_2_, N=C(NMe_2_)_2_ and N=P(NMe_2_)_2_), their pushing effects (although different) are cross-conjugated in an analogous way as in symmetrically disubstituted nitriles **I.13-I.17** and **II.13-II.17**.

Considering the different strengths of donor effects and different types of conjugation between the pushing and pulling groups, we differentiated in this work two subfamilies of nitriles in series **I** and **II**, the first one with simple substituents (Me and NR_2_) and the other one with larger, more complex, cross-conjugated substituents (N=C(NH_2_)_2_, N=C(NMe_2_)_2_, and N=P(NMe_2_)_3_). These subfamilies are labeled, respectively, as nitriles with simple substituents and nitriles with large substituents.

## 2. Methodology

As mentioned in the introduction, this work prolongs our previous study [9] on the gas-phase basicity of push–pull nitriles with the methylenecyclopropene- and cyclopropenimine-intercalated scaffolds. We applied here the same procedures of quantum chemical methods described previously [8,9]. In this way, we wish to examine qualitatively and quantitatively the major following points: (i) the stability of all reasonable potential isomeric forms for neutral and protonated nitriles, (ii) the favored site of protonation, (iii) enhancement of the gas-phase basicity when proceeding from the parent systems through methyl- and amino-substituted compounds (NR_2_, where R is H and/or Me) to derivatives containing very strong electron-donor substituent(s) (N=C(NR_2_)_2_ and N=P(NR_2_)_3_), (iv) the similarity of the total gas-phase substituent effects in series I (Z: CH) and II (Z: N), (v) the deviations from the additivity of the substituent effects when going from mono- to disubstituted derivatives, and also (vi) the bond length’s alternation in the isomeric neutral and protonated forms, particularly for the cyclopropene, methylenecyclopropene, and cyclopropenimine transmitters.

For our analysis, we employed the density functional theory (DFT) [10] with the hybrid B3LYP functional [11,12] and the extended 6-311+G(d,p) and/or 6-311++G(d,p) basis sets [13], abbreviated here as the DFT1 and DFT2 methods, respectively. The use of the same computational procedures allows minimization of the computational biases, and the proper examination of the push–pull effects in the derivatives studied here and previously [8,9]. For calculations, we applied the Gaussian programs [14,15].

Nitriles chosen in this work contain potential amino (N-sp^3^), imino (N-sp^2^), and cyano (N-sp) protonation sites. We showed in our previous articles [7,8,9,16] that the order of basicity in conjugated π-electron systems (N-sp > N-sp^2^ > N-sp^3^) is reversed in the gas phase when compared to that in non-conjugated bases (nitriles < imines < amines). We can generalize these previous studies showing that the N-sp^3^ amino atoms in NH_2_ and NR_2_ groups are the weakest basic sites in a monoprotonation process. For this reason, protonation at the amino N atoms has been neglected in this work.

By known procedures [1,2,4,9,17,18,19], basicity parameters such as PA (proton affinity, enthalpy term) and GB (gas-phase basicity, Gibbs energy term) were calculated *in vacuo* by quantum chemical methods only for the most probable protonation sites, the cyano and imino N atoms. Since the nitriles under scrutiny display conformational isomerism, PAs and GBs were estimated for most individual isomers and for isomeric mixtures. To distinguish these basicity parameters, we used different terms, microscopic (kinetic) basicity for individual isomers and macroscopic (thermodynamic) basicity for their mixture. The microscopic PA_i_ and GB_i_ refer to the acid-base equilibrium for individual isomer: B_i_H^+^ ⇆ B_i_ + H^+^, while the macroscopic PA_m_ and GB_m_ correspond to the equilibrium for the isomeric mixture: (*y*_1_B_1_H^+^ + *y*_2_B_2_H^+^ + *y*_3_B_3_H^+^ +…) ⇆ (*x*_1_B_1_ + *x*_2_B_2_ + *x*_3_B_3_ +…) + H^+^, where *x*_i_ and *y*_i_ are the molar fractions of neutral and protonated isomers.

Electron delocalization induced by the intercalation of methylenecyclopropene- and cyclopropenimine-transmitting scaffolds was analyzed by calculating the HOMED (Harmonic Oscillator Model of Electron Delocalization [20,21]) and HOMA (Harmonic Oscillator Model of Aromaticity [22]) indices in selected neutral and monoprotonated isomers.

The computational details are given in the Supplementary Materials (SM). Thermochemical data at 298 K and 1 atm such as the enthalpies (*H*) and Gibbs energies (*G*) for the neutral, cyano, and imino N-protonated forms of the unsubstituted compounds **I.1** and **II.1**, and also of all stable isomers of the mono- and disubstituted derivatives **I.1-I.17** and **II.1-II.17** are included in Table S1 (SM). Their microscopic basicity parameters (PA_i_ and GB_i_) are listed in Table S2 (SM). Selected bond lengths (C=Z, Z–CN, C–X, and C–Y) for neutral and cyano N-protonated derivatives are included in Tables S3 and S4 (SM). Energy barriers of configurational and conformational isomerisms in neutral and cyano-N-protonated forms of selected derivatives are summarized in Table S5 (SM). HOMEDs and HOMAs estimated for selected transmitter fragments are given in Tables S6 and S7 (SM). Partial substituent effects on PA_i_s for mono- and disubstituted nitriles are collected in Table S8 (SM). Percentage contents of neutral and protonated isomers and macroscopic basicity parameters for nitriles containing two different large substituents are given in Table S9.

## 3. Results and Discussion

### 3.1. Unsubstituted Compounds (***I.1*** and ***II.1***)

The unsubstituted bases **I.1** and **II.1** (Figure 1) belong to the family of conjugated heterocompounds. In our previous work in this series [9], we reported the gas-phase structures and acid-base properties of the symmetrically disubstituted derivatives **I.12-I.17** and **II.12-II.17**. The non-symmetrical substitution leads to a much larger number of isomers, for which we expect similar properties. In particular, the favored site of protonation in the gas phase, without any doubt, is the cyano N atom for **I.1** and **II.1** as well as for **I.12-I.17** and **II.12-II.17**. These nitriles display exceptional basicity in the gas phase when substituents are relatively strong electron donors, X or Y: NR_2_, N=C(NR_2_)_2_, and N=P(NR_2_)_3_. The imino N-protonated form of **II.1** (ZH^+^) and also of **II.12-II.17** can be neglected in the monoprotonation reaction. For **II.1**, its Gibbs energy is higher (i.e., less stable) than that of the cyano N-protonated ion (C≡NH^+^) by more than 50 kJ mol^−1^ (Table S1 in SM). Hence, the ZH^+^ form of **II.1** by its low concentration (<< 1 ppm), can be neglected for our purpose. The same is true for other derivatives (**II.12-II.17**), for which the *G*-difference is also high (>15 kJ mol^−1^).

The next property refers to the acid-base strength measured by gas-phase acidity-basicity parameters. Both the PA and GB of the cyano N atom in **I.1** and **II.1** were estimated in our previous work at the DFT, G2, and G2MP2 levels [9]. The two parent compounds seem to be stronger bases in the gas phase than Me_2_N–C≡N but weaker than Me_2_N–CH=CH–C≡N and Me_2_N–CH=N–C≡N [8]. Although the calculated PAs of **I.1** and **II.1** (respectively, 885 and 892 kJ mol^−1^, Table S2 in SM) are lower than the limit for compounds classified as superbases (PA > 1000 kJ mol^−1^) [2,23], attaching the methylenecyclopropene and cyclopropenimine groups to the nitrile strongly increases the PA of the cyano N atom (by ca. 200 kJ mol^−1^), by comparison with the PAs of HC≡N calculated at the same DFT or G2 level.

The imino N atom of the C=N group in **II.1** plays a particular role in this conjugated system. Instead of decreasing the electronic substituent effect of the cyclopropenimine group in comparison to that of the methylenecyclopropene one, it favorably interacts with the electron-accepting (pulling) C≡N function. The electronegative imino N atom in **II.1** withdraws the labile π-electrons present in the cyclopropenimine part, and transmits them to C≡N (Scheme S1 in SM). For the neutral and cyano N-protonated forms of **II.1**, a larger number of resonance structures can be written than for **I.1**, in connection with a more delocalized π-electron system [24,25]. Hence, the transmission of the electron-donating resonance effect of the cyclopropene cycle through the >C=N– bond to the cyano N-protonation site seems to be stronger in **II.1** than that through the >C=CH– bond in **I.1**. Indeed, the DFT-estimated HOMEDs, being geometrical measures of electron delocalization in the conjugated systems, highlight the important differences in the resonance and bond length’s alternation between the neutral forms of **I.1** and **II.1**, as well as between their neutral and cyano N-protonated forms [9].

### 3.2. Isomerism in Mono- and Disubstituted Derivatives

Isomeric phenomena possible in organic heterocompounds frequently influence the acid-base properties of individual isomers and isomeric mixtures. For this reason, they are considered significant for the mono- and disubstituted derivatives of N-bases investigated in this work. First, we considered the geometrical isomerism about the double C=Z bond in all unsymmetrically substituted derivatives, **I.2-I.11** and **II.2-II.11**. Then, we also considered the rotational isomerism about the single C (cyclopropene)–N (substituent) bonds for derivatives **I.5-I.11** and **II.5-II.11** containing large substituents (N=C(NR_2_)_2_ and/or N=P(NR_2_)_3_ and abbreviated here as N=A(NR_2_)_n_, where A is C or P, and n is 2 or 3, respectively).

Rotation of the NR_2_ and Me group(s) only slightly influences the stability of the conjugated system, and was not considered owing to the marginal geometric and thermochemical effects in comparison to those of the large donor substituent(s), N=A(NR_2_)_n_ [9]. We also neglected the prototropy possible in derivatives containing N=C(NH_2_)_2_ because the strong electron-withdrawing effect of the –C_2_HC=Z–C≡N part in **I.5** and **II.5** strongly favors the imino form (H_2_N)_2_C=N–C_2_HC=Z–C≡N. The same is true for **I.8** and **II.8**, for which the analogous effect of the –C_2_(NH_2_)C=Z–C≡N part also favors the imino form (H_2_N)_2_C=N–C_2_(NH_2_)C=Z–C≡N. The replacement of H by Me at the amino N atoms in the guanidino group eliminates prototropy in **I.6**, **I.9**, **II.6**, and **II.9**.

**Figure 2:**
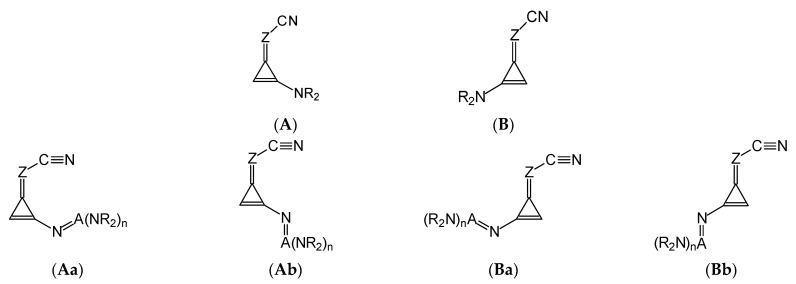
Geometrical (**A**,**B**) and rotational isomers (**a**,**b**) selected for monosubstituted derivatives investigated in this work.

Consequently, for analysis of the geometric, energetic, and basicity parameters of the investigated nitriles, two geometrical isomers were taken into account for derivatives containing one substituent X (**I.2-I.7**, and **II.2-II.7**): one isomer (**A**) with X at the *syn*-position and the other one (**B**) with X at the *anti*-position *vis-à-vis* C≡N (Figure 2). For derivatives containing the large substituents N=A(NR_2_)_n_, we additionally selected two extreme rotational isomers about the single C (cyclopropene)–N (substituent) bond: one *syn* isomer (**a**) and the other one *anti* (**b**) *vis-à-vis* C=Z at both *syn*- (**A**) and *anti*-positions (**B**) of N=A(NR_2_)_n_ *vis-à-vis* C≡N. In this way, two isomers (**A** and **B**) were selected for **I.2-I.4** and **II.2-II.4** with simple substituents (Me, NH_2_, and NMe_2_), while four isomers (**Aa**, **Ab**, **Ba**, and **Bb**) were analyzed for **I.5-I.7** and **II.5-II.7** containing large substituents (N=C(NH_2_)_2_, N=C(NMe_2_)_2_, and N=P(NMe_2_)_3_).

**Figure 3:**
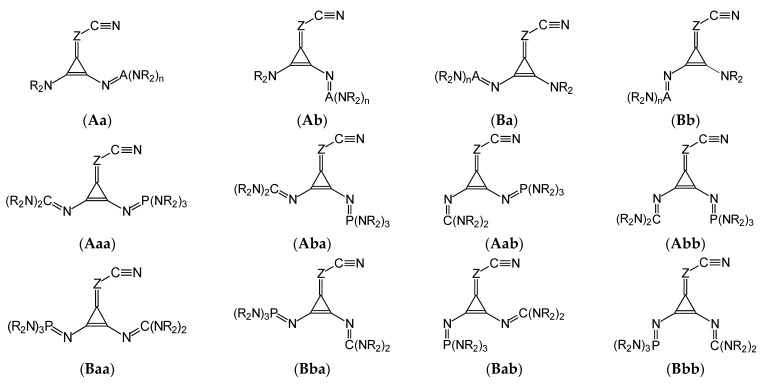
Isomeric structures considered in this work for unsymmetrically disubstituted derivatives containing one (**Aa**,**Ab**,**Ba**,**Bb**) and two large substituents (**Aaa**,**Aba**,**Aab**,**Abb**,**Baa**,**Bba**,**Bab**,**Bbb**).

The two types of isomerism (geometrical and rotational) are also possible for derivatives containing two different substituents, NR_2_ and N=A(NR_2_)_n_ (**I.8-I.10**, and **II.8-II.10**). Similar to derivatives with one large substituent (Figure 2), we examined four isomers (**Aa**, **Ab**, **Ba**, and **Bb**) for **I.8-I.10** and **II.8-II.10**, *syn* (**a**) and *anti* (**b**) rotamers *vis-à-vis* C=Z at both *syn*- (**A**) and *anti*-positions (**B**) *vis-à-vis* C≡N (Figure 3). A more complicated situation takes place for **I.11** and **II.11,** possessing two different large donor substituents, N=C(NR_2_)_2_ and N=P(NR_2_)_3_, for which the number of potential isomers is doubled, and eight isomers can be considered, **Aaa**, **Aab**, **Aba**, **Abb, Baa**, **Bab**, **Bba**, and **Bbb** included in Figure 3. For symmetrically disubstituted derivatives with two large N=A(NR_2_)_n_ groups (**I.15-I.17** and **II.15-II.17**), only the rotational isomerism about the single bonds C (cyclopropene)–N (X) was investigated, and the four isomers (**aa**, **ba**, **ab**, and **bb**) discussed in Part I of this study (as **a**–**d**, respectively) [9].

The isomeric phenomena were examined for all neutral as well as cyano and imino N-protonated forms of nitriles containing the methylenecyclopropene (**I.2-I.11**) and cyclopropenimine (**II.2-II.11**) scaffolds at the DFT1 and/or DFT2 level. Except for the derivatives with two large substituents (**I.11** and **II.11**), structures for all isomers considered in this work were found to be stable. For the unsymmetrically disubstituted derivatives **I.11** and **II.11**, only six isomers (**Aaa**, **Aab**, **Aba**, **Baa**, **Bab**, and **Bba**) were stable for both the neutral and protonated forms, analogously as for the symmetrically disubstituted derivatives **I.15-I.17** and **II.15-II.17**, for which only three isomers (**aa**, **ba**, and **ab**) were previously found to be stable. The conformation **bb** for **I.15-I.17** and **II.15-II.17**, and also the two conformations **Abb** and **Bbb** for **I.11** and **II.11** were unstable for both the neutral and imino N-protonated forms. Only structures for the cyano N-protonated forms were found to be stable for these conformations. Table S1 (SM) summarizes the thermochemical data such as enthalpy (*H*) and Gibbs energy (*G*) at 298 K and 1 atm that characterize the stability of the isomers studied here for the two series of nitriles, **I.2-I.11** and **II.2-II.11**. Note that data for the derivatives already investigated in Part I (**I.1**, **I.12-I.17**, **II.1**, and **II.12-II.17**) are also included in this table for easier comparison.

In the following step, we calculated the relative Gibbs energies between these isomers to indicate the most favored structures separately for neutral and monoprotonated forms. Their values are included in Figures S1–S4 (SM). For neutral derivatives containing one simple substituent (**I.2-I.4** and **II.2-II.4** in Figure S1), the isomer **A** has slightly lower Gibbs energy than **B** (Δ*G* < 6 kJ mol^−1^). A reverse situation takes place for their cyano N-protonated forms. The isomer **B** possesses lower Gibbs energy than **A** (Δ*G* < 6 kJ mol^−1^). The isomeric preference and order of substituent effects are analogous in both series (**I** and **II**).

For neutral mono- and disubstituted derivatives containing one large electron-donor substituent N=A(NR_2_)_n_ (**I.5-I.10** and **II.5-II.9** in Figures S2 and S3), the isomer **Aa** is favored. Important stability also characterizes the isomer **Ba** (Δ*G* < 10 kJ mol^−1^), which predominates for **II.10** (Figure S3). Isomeric preferences are not the same for nitriles with two large electron-donor substituents (**I.11** and **II.11**). The isomer **Bab** predominates for **I.11**, while **Baa** for **II.11** (Figure S4). However, the isomeric mixtures of **I.11** and **II.11** can also contain other isomers (**Aaa**, **Aba**, **Aab**, and **Bba** with Δ*G* ≤ 12 kJ mol^−1^).

Protonation changes the isomeric preferences, and therefore influences the composition of isomeric mixtures for monocations (Figures S2–S4 in SM). The cyano N-protonated form of the isomer **Ab** is favored for **I.5-I.7** and **II.6-II.7**, whereas the cyano N-protonated form of **Ba** seems to be more stable for **II.5**, **I.8-10**, and **II.8-II.10** (Figures S2 and S3 in SM). Note that the Gibbs energies of some other cyano N-protonated isomers do not differ very much from those of the favored ones (Δ*G* ≤ 10 kJ mol^−1^), and can also, to various degrees, affect physicochemical properties of the N-protonated forms. For example, the Gibbs energies of the cyano N-protonated isomer **Bb** and **Ab** for **I.5-I.7**, and **Ba** and **Ab** for **II.5-II.7**, differ by no more than 2 kJ mol^−1^, and those of the isomers **Aa** and **Ba** for **I.8-I.10** and **II.9** differ by less than 1 kJ mol^−1^. They can predominate in the isomeric mixtures of **I.5-I.10** and **II.5-II.7** and **II.9**, therefore determining their properties. In the case of **I.11** and **II.11** (Figure S4 in SM), the isomer **Aba** is favored for the cyano N-protonated form. Nevertheless, other isomers, particularly **Bba** and **Bab** for **I.11**, and **Bba**, **Baa**, **Bab**, **Aab,** and **Aaa** for **II.11**, have very close Gibbs energies to that of **Aba** (Δ*G* ≤ 12 kJ mol^−1^). Note that the Gibbs energy of the guanidino N-imino protonated isomer **Baa** of **I.11** is only slightly above that of the favored cyano-protonated derivative (Δ*G* ca. 8 kJ mol^−1^).

Structural and energetic differences between the geometric and rotational isomers of the investigated nitriles influence the basicity parameters calculated for each potential N-protonation site in the C≡N and C=N (Z, X, and Y) groups. The microscopic (kinetic) basicity parameters (PA_i_ and GB_i_), calculated at the DFT level for these sites according to Equations (S1)–(S3) given in SM, are listed in Table S2 (SM). Most estimated PA_i_s are close to or even higher than 1000 kJ mol^−1^. This means that many derivatives investigated here can be classified into the family of strong organic N-bases such as guanidines and phosphazenes [1,2,4].

### 3.3. Favored Site of Protonation

Similar to the investigated push–pull nitriles containing the unsubstituted methylenecyclopropene and cyclopropenimine groups (**I.1** and **II.1**) and symmetrically disubstituted derivatives (**I.12-I.17** and **II.12-II.17**) with electron-donor groups, previously investigated in Part I [9], the cyano N atom appears to be the favored site of protonation also in the unsymmetrically substituted derivatives **I.2-I.11** and **II.2-II.11** examined here (Figure 1). The Gibbs energies estimated for isomers protonated at the imino N atom in C=Z, X, or Y (N=A(NR_2_)_n_) groups are higher than those protonated at the cyano N atom by ca. 10–120 kJ mol^−1^ (Figures S1–S4 in SM). In our previous articles on the basicity of amino, guanidino, and phosphazeno nitriles [7,8,9], we also established computationally that protonation at the amino N atom is negligible. The favored site of protonation (cyano N atom) in compounds of series **I** and **II** is a consequence of the strong conjugation between the electron-acceptor (C≡N) and electron-donor (XYC_2_C=Z) groups in which the C_2_C=Z scaffolds act as good transmitters for the electronic substituent effects of X and Y.

A perusal of the C (cyclopropene)=Z and Z–C (cyano) bond lengths in the mono- and disubstituted push–pull nitriles **I.2-I.11** and **II.2-II.11** (see Table S3 in SM) suggests that the energy barriers for geometrical isomerism can be considerably lower than those in the case of unconjugated substituted alkenes and imines. The cyano N-protonation shortens the Z–C bond (by 0.07–0.08 and 0.08–0.09 Å for series **I** and **II**, respectively) and lengthens the C=Z bond (by 0.05–0.06 and 0.03–0.05 Å for series **I** and **II**, respectively). These effects can facilitate the rotation of the cyano group about the C=Z bond (Figure S5 and Table S5 in SM), lengthened by stronger electron delocalization in the cyano N-protonated forms. Moreover, the C=Z bond in the ionic forms (1.40–1.43 Å for **I** and 1.32–1.36 Å for **II**) and even in neutral isomers (1.35–1.38 Å for **I** and 1.29–1.32 Å for **II**) are longer than those of the double C=C and C=N bonds, calculated at the same DFT level for H_2_C=CH_2_ (1.33 Å) and H_2_C=NH (1.27 Å). In many protonated forms of nitriles, they are also longer than those for the aromatic molecules: benzene (1.39 Å) and *s*-1,3,5-triazine (1.33 Å), in which π-electrons are completely delocalized and there are no typical double C=C and C=N bonds. This observation indicates that the rotation of the cyano group about only partially double C=Z bonds in nitriles (**I** and **II**) can be less restricted than those in alkenes and imines containing unconjugated groups [26,27,28,29,30].

Smaller variations in bond lengths when proceeding from the neutral to cyano N-protonated forms are found for the C–X and C–Y bonds (all C–N) about which substituents X and Y can rotate. For large electron-donor substituents (N=A(NR_2_)_n_), these bonds shorten by ca. 0.02–0.05 Å (Table S4 in SM). In the neutral forms, bond lengths (1.33–1.37 Å) are close to or even longer than that for aromatic *s*-1,3,5-triazine, whereas they are slightly shorter for the monocations (1.30–1.33 Å). These variations indicate that rotation about the C–X and C–Y bonds can be slightly more restricted for the protonated forms for which n–π conjugation appears stronger than for the neutral ones. However, energy barriers can be of the same (or even lower) order of magnitude than those for isomerism about the C=Z bonds.

Based on our theoretical analyses on the relative Gibbs energies for the neutral and N-protonated (cyano and imino) derivatives, and additionally on the C=Z, C–X, and C–Y bonds lengths for investigated isomers, we can conclude with a high probability that in the gas phase (i) unsymmetrically substituted nitriles (**I.2-I.11** and **II.2-II.11**) can be in the form of mixtures of geometric (**A** and **B**) and rotational (**a** and **b**) isomers, (ii) the cyano N atom is preferentially protonated, and (iii) protonation of the imino N atom may be considered only in a few cases: ZH^+^ for **II.11**, and X(or Y)H^+^ for **I.6**, **I.7**, **I.9**, **I.10**, and **I.11**. However, from a physicochemical point of view, their participation in the isomeric mixture of monocations does not appear to be very significant (8 ≤ Δ*G* ≤ 20 kJ mol^−1^).

### 3.4. Electron Delocalization in Mono- and Disubstituted Derivatives

The addition of one or two substituents such as Me, NR_2_, and N=A(NR_2_)_n_ at the C atoms of the cyclopropene ring in the parent systems **I.1** and **II.1** (Figure 1), influences the distribution of all labile n- and π-electrons in monosubstituted (**I.2-I.7** and **II.2-II.7**) and unsymmetrically disubstituted derivatives (**I.8-I.11** and **II.8-II.11**) investigated in this work, as in the symmetrically disubstituted derivatives (**I.12-I.17** and **II.12-II.17**) already studied in Part I [9]. The pushing effects of the amino, guanidino, and phosphazeno groups have been documented for other π-electron N-bases, their structures and thermochemical properties (particularly for imines) [1,2,4,5,31,32,33]. In the case of nitriles with the methylenecyclopropene and cyclopropenimine transmitters, the pushing effects of NR_2_ and N=A(NR_2_)_n_ can be qualitatively illustrated by various resonance structures that can be written for their neutral and protonated forms (Schemes S2 and S3 in SM). Using the appropriate quantitative descriptors of electron delocalization, we can distinguish significant changes when proceeding from the neutral to protonated forms, particularly in the transmitter parts that are common for all derivatives of series **I** and **II**, studied here and previously [9].

To evaluate the electron delocalization quantitatively in the neutral and cyano N-protonated forms of new mono- and disubstituted derivatives, we applied the HOMED procedure [20,21]. We calculated the HOMED indices (see Computational details in SM) for the three-bonds transmitter (cyclopropene ring, HOMED3) in series **I** and **II**, and for two four-bonds transmitters (methylenecyclopropene in **I,** cyclopropenimine in **II**, HOMED4) according to Equation (S9). These values were compared with the calculated HOMED values found for the parent compounds and for the symmetrically disubstituted derivatives already reported in Part I [9]. The HOMA procedure [22] has also been applied, and the HOMA values estimated for the cyclopropene ring in the neutral and protonated nitriles. For selected derivatives, the estimated HOMED3s and HOMED4s are listed in Table S6, and HOMAs in Table S7 (SM).

For the Me derivatives **I.2** and **II.2**, the values of HOMED3 and HOMED4 do not vary very much for isomers **A** and **B** for the neutral and cyano N-protonated forms (Table S6 in SM). Generally, the HOMED differences are not larger than 0.007 HOMED units. HOMEDs are also higher for the protonated than for the neutral forms of **I.2** and **II.2** by 0.10–0.14 and 0.05–0.09 HOMED units, respectively. This HOMED trend is analogous to that for the parent compounds, **I.1** and **II.1**, for which HOMEDs increase (by 0.09–0.14 and 0.05–0.09, respectively) when going from the neutral to protonated forms (Scheme S1 in SM). When compared to the parent compounds **I.1** and **II.1**, the HOMED values for the neutral and cyano N-protonated forms of **I.2** and **II.2** are slightly higher by 0.01–0.03 HOMED units, confirming some electron-donor (polarizability and σ-π hyperconjugation) effects of Me. Stronger effects (shift by 0.03–0.06 HOMED units) occur for derivatives **I.12** and **II.12** containing two Me groups [9]. The HOMED trend for the neutral and ionic forms of **I.12** and **II.12** is analogous to that for **I.2** and **II.2**. The HOMED values increase when going from the neutral to ionic forms by 0.10–0.13 and 0.05–0.09, respectively.

**Scheme 1:**
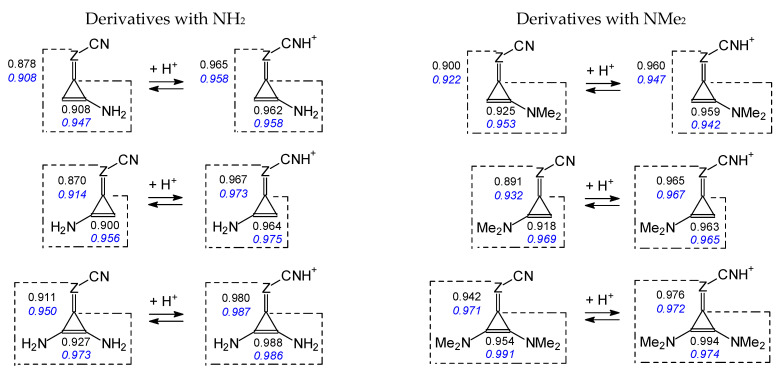
HOMED3 (cyclopropene cycle) and HOMED4 (methylenecyclopropene and cyclopropenimine scaffolds) in amino derivatives when going from the neutral to the cyano N-protonated forms (italicized blue values for series **II** of imino nitriles, Z: N). HOMED data for disubstituted derivatives taken from Part I [9].

Variations in HOMED3 and HOMED4 values in the amino (NH_2_ and NMe_2_) derivatives, monosubstituted and symmetrically disubstituted, can be observed in Scheme 1. Except for the HOMED4 values for isomers **A** and **B** of the neutral NMe_2_ nitrile (**I.4**), isomerization effects are not significant for these compounds. HOMEDs for isomers **A** and **B** of monosubstituted nitriles do not differ by more than 0.02 HOMED units. Some differences take place between mono- and disubstituted compounds of series **I** and **II** when proceeding from the neutral to protonated forms. The HOMED values are higher for the protonated (0.96–0.99) than for neutral forms (0.85–0.95) in the NH_2_ and NMe_2_ derivatives of series **I**, as for the parent compound **I.1**. Nevertheless, only for the neutral forms, the NMe_2_ group(s) cause a slight increase in HOMEDs in comparison to the NH_2_ one(s). For the ionic forms, the effects of the NH_2_ and NMe_2_ groups are almost the same, indicating some kind of saturation of electron delocalization in the π-electron systems or some additional favorable interactions between the substituent and other functions. A slightly different situation takes place for the NH_2_ and NMe_2_ derivatives of series **II**. The HOMED values do not vary so much when proceeding from the neutral to protonated forms as for series **I**. For the NMe_2_ derivatives, the HOMED values even decrease for monocations. This can be explained by some kind of unfavorable steric interaction (repulsion) of the larger NMe_2_ group (their CH parts) with a positively charged system. Only for the neutral forms, the trend of substituent effects is similar to that for series **I**, i.e., HOMEDs estimated for the NMe_2_ derivatives are slightly higher than those for the NH_2_ ones.

**Figure 4:**
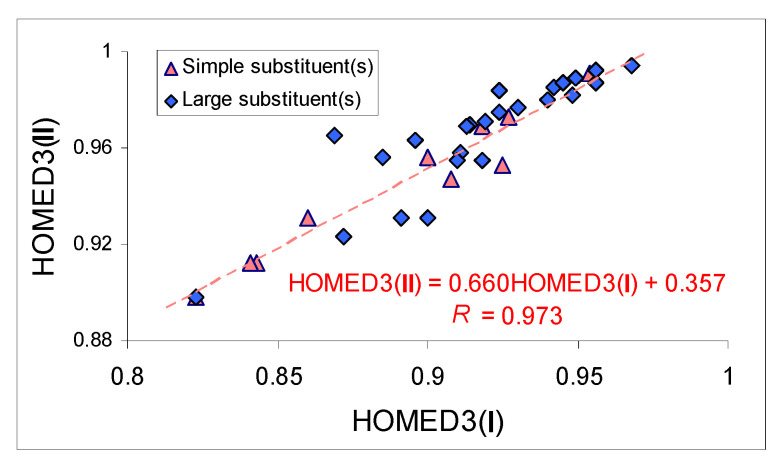
Linear trend between HOMED3s estimated for mono- and disubstituted nitriles of series **I** and **II** containing simple group(s) (H, Me, NH_2_, and NMe_2_) and one or two large substituents (N=C(NH_2_)_2_, N=C(NMe_2_)_2_, and N=P(Me_2_)_3_). HOMED3 data taken from Table S6 (SM).

Taking into account the small differences between the HOMED values of isomers **A** and **B**, and almost analogous HOMED orders in neutral monosubstituted and symmetrically disubstituted derivatives containing simple groups (H < Me < NH_2_ < NMe_2_), we plotted the HOMED3s estimated for nitriles of series **II** against those of series **I**. Indeed, some parallelism exists between the corresponding HOMED values, and a quite good linear relationship is found with a correlation coefficient *R* equal to 0.973 (Figure 4). Note that points corresponding to the isomers **A** of the monosubstituted derivatives containing NH_2_ and NMe_2_ slightly deviate from this line, plotted for a subfamily of nitriles with simple substituent (s). A different situation in HOMED variations and more significant deviations from the linear trend occur in the other subfamily containing large substituents.

A comparison of the HOMED values estimated for neutral nitriles containing N=C(NR_2_)_2_ and N=P(NR_2_)_3_ (Table S6 in SM) shows that owing to their strong pushing character, the HOMED values increase for the selected structural transmitters to a higher degree for disubstituted than for monosubstituted compounds, and generally, in parallel to the increase in the substituent pushing effects. However, HOMED differences between the geometrical and rotational isomers are considerably higher for these derivatives than those for nitriles with simple substituent(s). For example, the highest isomeric effects (0.03–0.05 HOMED units) occur for **I.5** and **II.5** and the smallest ones (≤0.02 HOMED units) for **I.8-I.10** and **II.8-II.10**. Hence, deviations of points from the linear trend given in Figure 4 are considerably greater for derivatives with large substituent(s) than for those with simple group(s). They are greater for **I.5-I.7** and **II.5-II.7** than for **I.8-I.10** and **II.8-II.10**.

Moreover, the HOMED indices estimated for compounds of series **I** with large substituent(s) increase when proceeding from the neutral to cyano N-protonated forms. In series **II**, the HOMED differences are considerably smaller, and in some cases, even the HOMED values for monocations are lower than those for neutrals. Some saturation of electron delocalization for monocations, already signaled for strong electron donor groups in the symmetrically disubstituted derivatives reported in Part I [9], is also observed for the monosubstituted and unsymmetrically disubstituted nitriles studied here. Consequently, the HOMED3 indices referring to the cyclopropene ring are close to unity for most of the protonated nitriles as for aromatic systems (benzene), indicating a strong electron delocalization. Owing to the significant differences between the isomers and the relatively small variations in the HOMED3 values (0.9–1.0) for monocations, a comparison of their values for the nitriles of series **I** and **II** is instead presented in a scatter plot (Figure S6 in SM).

Since the HOMA index is based on the already delocalized reference molecule (butadiene) [22], values of this descriptor (Table S7 in SM) are considerably smaller than those of HOMED3 based on the almost non-delocalized reference molecules (ethane and ethene) [20,21], which are also applied in the original HOMA procedure [34,35]. The use of different reference molecules for the C–C and C=C bond lengths in the hypothetical structure of cyclohexatriene leads to a different “zero” in the HOMA and HOMED scales. They correspond to moderately and non-delocalized cyclohexatriene, respectively. Only unity in the HOMA and HOMED scales refers to the same completely delocalized benzene molecule. However, the general HOMA increase trend for the investigated nitriles is analogous to that of HOMED. The HOMA values are higher for the cyano N-protonated than for the neutral forms. They also increase when the pushing effects of substituent(s) are enhanced.

### 3.5. Microscopic Basicities and Substituent Effects

All nitriles studied here and previously, and containing the methylenecyclopropene and cyclopropenimine intercalated scaffolds unsymmetrically and symmetrically substituted by electron-donor group(s), **I.2-I.17** and **II.2-II.17**, can be classified into the family of nitriles (**I**) and iminonitriles (**II**), respectively. They can be considered as homolog families of conjugated (push–pull) nitriles. Their basicity parameters as well as substituent and push–pull effects can be quantitatively compared according to the methods of LFER (Linear Free Energy Relationship) analysis, similar to those reported in the literature for a series of homolog π-electron systems having the same site of protonation/deprotonation and different substituents, or, in other words, for series of acids and bases for which the mechanism of protonation/deprotonation is the same [36,37,38,39].

**Figure 5:**
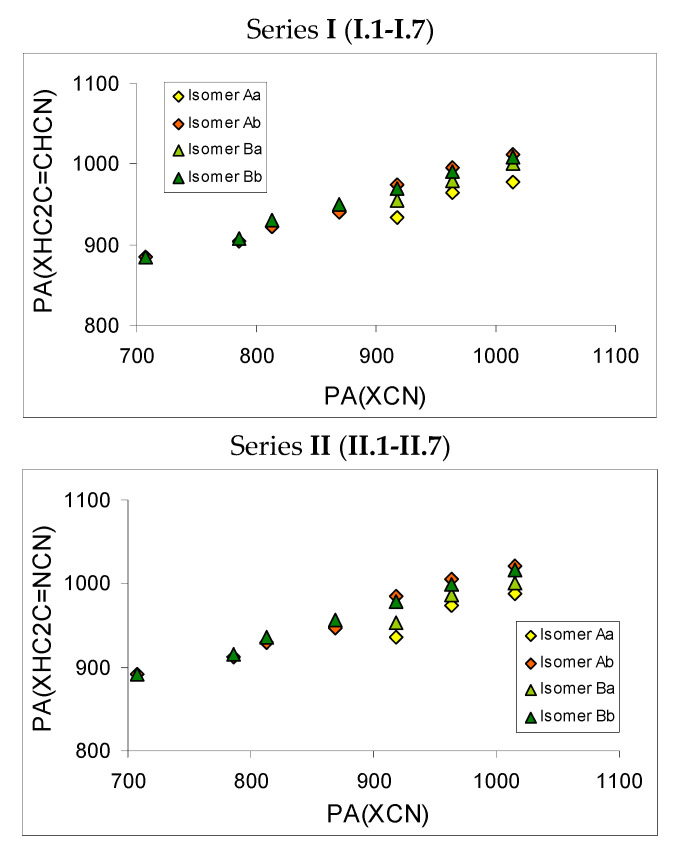
Linear trends between PA_i_s (in kJ mol^−1^) estimated for monosubstituted nitriles investigated in this work, XHC_2_C=CH–C≡N (**I.2-I.7**) and XHC_2_C=N–C≡N (**II.2-II.7**), and previously, X–C≡N. DFT data for XCN taken from [8].

Taking into account the microscopic gas-phase basicity parameters (PA_i_s and GB_i_s) given in Table S2 (SM) for compounds of series **I** and **II**, we can make the following observations. Generally, the PA_i_s and GB_i_s of monosubstituted nitriles **I.2-I.7** and **II.2-II.7** are enhanced in parallel to a greater size than substituent X and its stronger electron-donor effect, which increases as follows: Me < NH_2_ < NMe_2_ < N=C(NH_2_)_2_ < N=C(NMe_2_)_2_ < N=P(NMe_2_)_3_. This general order is analogous to that reported previously for series of nitriles X–C≡N containing substituent X directly bonded to the C atom of the C≡N group [7,8]. For nitriles studied here, PA_i_s and GB_i_s depend additionally on possible geometrical and/or rotational isomerism. For derivatives **I.2-I.4** and **II.2-II.4** with simple substituents (Me, NH_2_, and NMe_2_), the geometrical isomerism differentiates PA_i_s of the isomers **A** and **B** by 2–10 kJ mol^−1^. Geometrical and rotational isomerisms, possible in **I.5-I.7** and **II.5-II.7** containing one large substituent (N=C(NH_2_)_2_, N=C(NMe_2_)_2_, and N=P(NMe_2_)_3_), cause considerably stronger effects; PA_i_s for isomers **Aa**, **Ab**, **Ba**, and **Bb** vary by 10–50 kJ mol^−1^. Figure 5 illustrates these substituent and isomeric effects on PA_i_s for monosubstituted derivatives of series **I** (**I.2-I.7**) and **II** (**II.2-II.7**). The point corresponding to PAs of the parent compounds **I.1** and **II.1** is also included in this figure. Although PA_i_s for geometrical and rotational isomers vary in a different way, linear trends exist between substituent isomeric effects for the monosubstituted derivatives studied here (**I.1-I.7** and **II.1-II.7**) and previously (X–C≡N) [8]. In our previous report, analogous linear trends were described for the symmetrically disubstituted derivatives **I.12-I.17** and **II.12-II.17** [9].

**Figure 6:**
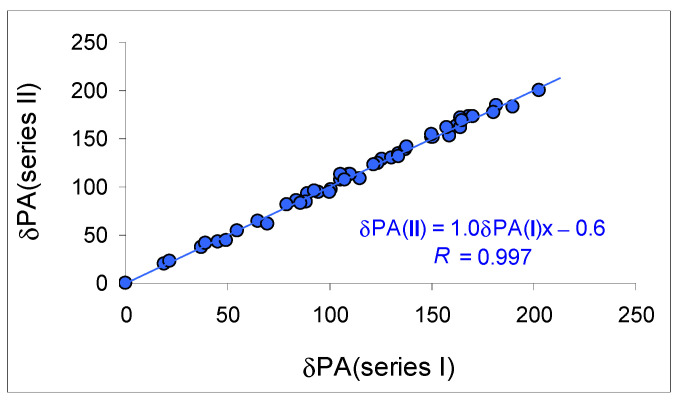
Linear relationship between total electronic substituent effects (δPA in kJ mol^−1^) estimated for isomers of series **I** and **II.**

The gas-phase total substituent effect for each isomer of **I.2-I.17** and **II.2-II.17** can be estimated as a difference between the DFT-calculated PA_i_ (or GB_i_) of substituted derivatives and that of the corresponding parent system, **I.1** and **II.1**, respectively. This difference is usually abbreviated in the literature as δPA_i_, and, in the case of the nitriles studied here, corresponds to the sum of the electronic substituent effects, intramolecular interactions between the substituent and N-protonation or other N sites, and steric effects. For δPA_i_ estimation, we considered all isomers of the monosubstituted nitriles (**I.2-I.7** and **II.2-II.7**) and unsymmetrically disubstituted derivatives (**I.8-I.11** and **II.8-II.11**) investigated in this work, and also the symmetrically disubstituted compounds (**I.12-I.17** and **II.12-II.17**) previously reported in Part I [9]. Figure 6 presents an excellent linear relationship between δPA_i_s determined for all isomers of series **I** (**I.2-I.17**) and **II** (**II.2-II.17**). This relationship (with a correlation coefficient *R* equal to 0.997) indicates that generally the structural replacement of the CH group in series **I** by the imino N atom in series **II** does not destroy π–π and n–π conjugation in the π-electron system nor transmission of the pushing substituent effects to the pulling C≡N group (protonation center). Some small deviations of points for derivatives containing the N=C(NH_2_)_2_ (**5Aa**, **5Ba**, **8Ba**) or N=P(NR_2_)_3_ group (**7Ba**, **10Ba**, **11Bba**) can be attributed to different favorable and unfavorable intramolecular interactions or steric effects between various functional groups, e.g., amino, imino, and cyano, as signaled previously for symmetrically disubstituted derivatives [9]. Figure S7 (SM) shows some intramolecular interactions for derivatives with one or two N=C(NH_2_)_2_ groups.

### 3.6. Partial Substituent Effect and Additivity

All substituents chosen in this work are electron donors. Me is σ–π hyperconjugated as in toluene, NR_2_ is n–π conjugated, N=C(NR_2_)_2_ (two NR_2_ and C=N as transmitter) is n–π cross-conjugated (double-conjugated or Y-conjugated), and N=P(NR_2_)_3_ (three NR_2_ and P=N as transmitter) is n–π cross(triple)-conjugated. We consider them as “simple conjugated” (Me, NR_2_) and “cross-conjugated” (N=C(NR_2_)_2_ and N=P(NR_2_)_3_) substituents, or “simple” and “large” (or “more complex”) groups.

Although possessing a high predictive value, the linear relationship (presented in Figure 6) gives no information about the partial gas-phase substituent effects of the investigated simple and large groups for mono- and disubstituted derivatives. Without additional analysis, it is not possible to answer the question of the additivity or non-additivity of the substituent effects. For this reason, substituent effects were separately estimated and analyzed for each derivative. First, nitriles containing simple substituents X (Me, NH_2_, and NMe_2_) were examined. Starting from PA_i_s of the parent compounds (**I.1** and **II.1**) and proceeding to the isomers **A** and **B** of **I.2-I.4** and **II.2-II.4**, and next, going from **A** and **B** of **I.2-I.4** and **II.2-II.4** to symmetrically disubstituted nitriles **I.12-I.14** and **II.12-II.14**, the partial substituent effects on PA_i_s of the cyano N atom were calculated and are given in Scheme S4 (SM). This analysis shows that transmission of the substituent effects from the *syn*-position *vis-à-vis* C≡N in the isomer **A** of **I.2-I.4** and **II.2-II.4** is slightly weaker than that from the *anti*-position in the isomer **B**. Due to the small distance between *syn*-X and C≡N, some additional unfavorable intramolecular effects can also take place. Furthermore, introduction of the second substituent Me and NMe_2_ in the cyclopropene ring of **I.2, II.2**, **I.4**, and **II.4** causes weaker effects on PA_i_s than that of the first substituent (*ca*. 90% and 70–90%, respectively). This suggests that substituent effects are not additive in the cross-conjugated disubstituted derivatives **I.12**, **I.14**, **II.12**, and **II.14**. A slightly different trend is observed for the NH_2_ derivatives **I.13** and **II.13**. Effects of the second group are slightly stronger (≤110%) than those of the first one for both isomers, **A** and **B**.

**Scheme 2:**
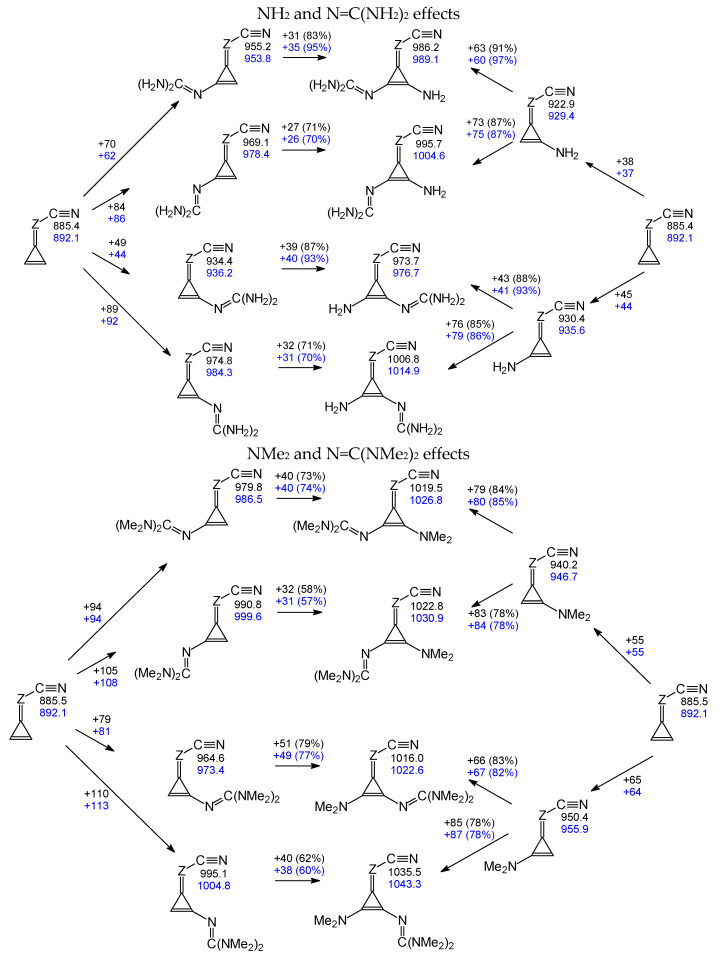

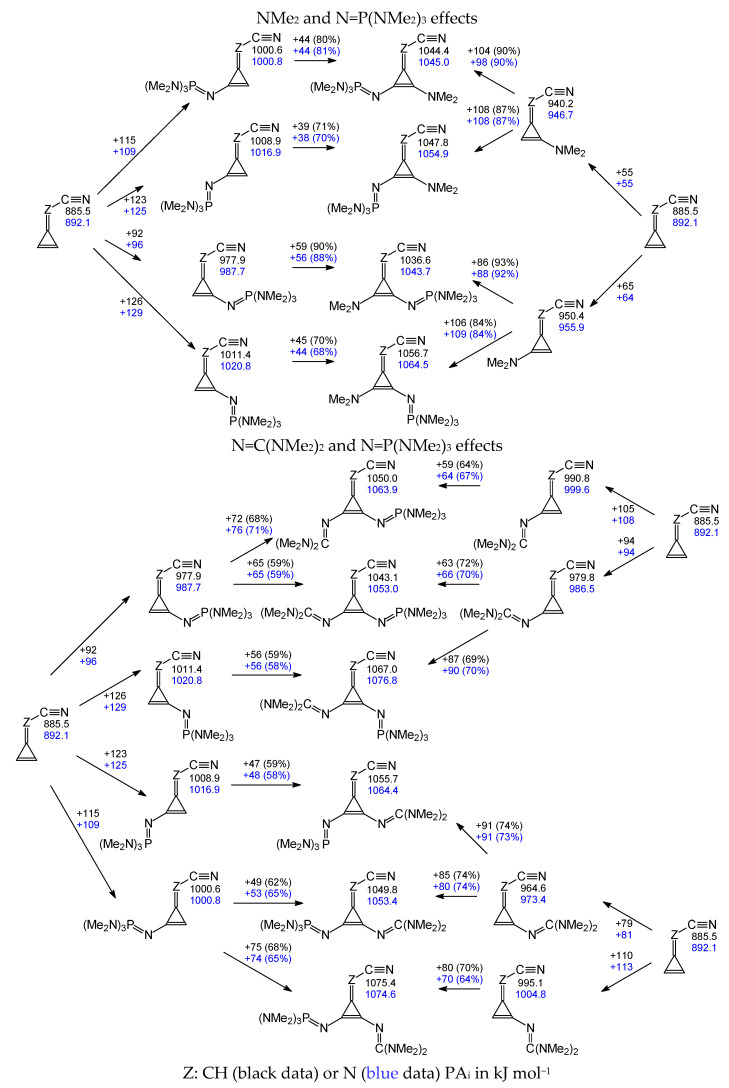
Comparison of partial effects (in kJ mol^−1^) of NH_2_, NMe_2_, N=C(NH_2_)_2_, N=C(NMe_2_)_2_, and N=P(NMe_2_)_3_ on DFT-calculated PA_i_s of the cyano N atom in monosubstituted and unsymmetrically disubstituted nitriles studied in this work. The indicated percentages correspond to the attenuation of the effect observed when introducing a substituent on the unsubstituted compound with the effect observed when introducing this substituent, at the same position and, when possible, in the same conformation, on the compound already bearing another substituent.

Similarly, the partial electronic substituent effects on PA_i_s of the cyano N atom can be examined for mono- and disubstituted derivatives containing large substituents (N=C(NH_2_)_2_, N=C(NMe_2_)_2_, and N=P(NMe_2_)_3_). Proceeding from the parent compounds (**I.1** and **II.1**), the effects of the first substituent in the isomers **Aa**, **Ab**, **Ba**, and **Bb** of the monosubstituted derivatives (**I.5-I.7** and **II.5-II.7**), then those of the second substituent in the unsymmetrically and symmetrically disubstituted nitriles (**I.8-I.17** and **II.8-II.17**) were calculated. Scheme S5 (SM) illustrates the partial substituent effects estimated in this way for the monosubstituted (**I.5-I.7** and **II.5-II.7**) and symmetrically disubstituted derivatives containing N=C(NH_2_)_2_, N=C(NMe_2_)_2_, and N=P(NMe_2_)_3_ (**I.15-I.17** and **II.15-II.17**). On the other hand, the partial substituent effects estimated for the unsymmetrically disubstituted derivatives with NH_2_ and N=C(NH_2_)_2_, NMe_2_ and N=C(NMe_2_)_2_, NMe_2_ and N=P(NMe_2_)_3_, and N=C(NMe_2_)_2_ and N=P(NMe_2_)_3_ (**I.8-I.11** and **II.8-II.11**) are compared in Scheme 2. Generally, no additivity of the substituent effects takes place for the derivatives with large substituent(s). However, some parallelism of partial substituent effects is observed between the series of mono- and disubstituted nitriles containing the same type of substituent, X in monosubstituted nitriles and Y in disubstituted derivatives. Some examples are included in Figure S8 (SM), showing the degree of non-additivity that can be expected when comparing mono- and disubstituted series.

Adding donor substituents on the transmitter should increase the basicity of the nitrile. Note that cross conjugation of substituents can depend on various factors such as the structure of substituents, their position *vis-à-vis* C≡N, and conformation *vis-à-vis* C=Z. The additivity of the effects of two substituents can be tested formally by comparing, in an arbitrary order, the effect observed when introducing a substituent on the unsubstituted compound with the effect observed when introducing this substituent at the same position and, when possible, in the same conformation, on the compound already bearing another substituent. Therefore, deviation from additivity is calculated here as the ratio of the latter to the former expressed as a percentage, given in Scheme 2 (see also Schemes S4 and S5 in SM). Perfect additivity would correspond to a 100% ratio. Actually, these percentages are in almost all cases lower than 100%, highlighting a deviation from the additivity of the donor effects of each substituent. These observations are discussed in the following on the grounds of partial saturation of the resonance effect.

If the two substituents are identical, the difference in their respective effects comes only from the initial position of the first group relative to C≡N (on the same side or opposite). The nitriles of series **I** and **II** bearing two identical substituents were studied earlier [9] and their basicities can now be compared with those of the monosubstituted systems (Schemes S4 and S5 in SM). For the methyl substitutions, the attenuation of the effect of the second Me is about 90% for both series, and the order of introduction has a relatively small effect. For the NMe_2_ substitution, a significant difference is observed between series **I** and **II**, with a respective attenuation of about 75% and 90%. For the NH_2_ substitution, we obtain an unexpected deviation from additivity, with values in the range 106–110%, meaning that there is a synergy, instead of a competing donor effect, between the two groups. This can be partially explained by differences in geometries (consequently, in conjugations) of neutral monosubstituted and disubstituted derivatives. Note that NH_2_ is planar in the DFT structures of monosubstituted neutral compounds, while in disubstituted ones two amino groups take the tetrahedral conformation. In cyano-protonated derivatives, the amino groups are planar in both types of derivatives.

For the larger substituents, the problem is complicated by their different conformations and their combinations (Scheme S5 in SM) when testing the additivity. The comparison was made between groups in the same position and conformation. Additionally, intramolecular interactions can also affect the substituent effects and, consequently, basicity of the N-cyano site. Some examples of intramolecular interactions for guanidino (N=C(NH_2_)_2_) derivatives are shown in Figure S7 (SM). Targeting the superbases, we consider the electron-donor groups in positions and conformations leading to the largest basicity increases. Within this category of effects, the addition of the second substituent is about 60–70% for N=C(NMe_2_)_2_ and N=P(NMe_2_)_3_, with little difference between series **I** and **II**. Notably, the basicity enhancement by the second substituent is reduced by only 75–90% by the weak donors (Me, N(Me_2_)) but by 60–70% for the strong donors (N=C(NMe_2_)_2_ and N=P(NMe_2_)_3_), indicating the partial saturation effect mentioned earlier. The strength of electron donors, deduced from the addition of the first substituent to **1**, is as follows: Me < NH_2_ < NMe_2_ < N=C(NH_2_)_2_ < N=C(NMe_2_)_2_ < or ≈ N=P(NMe_2_)_3_.

We expect to gain more information from the partial substituent effects on the basicity of unsymmetrically disubstituted derivatives **I.8-I.11** and **II.8-II.11**. In these cases, the order, in which substitution is introduced, changes the attenuation of the effects of the second substitution, as observed in Scheme 2. For the discussion, we consider only the electron-donor groups in the position and conformation leading to the largest basicity increases.

When going from derivatives bearing the simple substituent NH_2_ or NMe_2_, attenuations for the effect of the second substituent N=C(NH_2_)_2_, N=C(NMe_2_)_2_, and N=P(NMe_2_)_3_ vary from 78 to 97% in series **I** and **II**. On the other hand, proceeding from a nitrile already containing the large substituents N=C(NH_2_)_2_, N=C(NMe_2_)_2_, and N=P(NMe_2_)_3_, attenuations for the second simple substituent NH_2_ and NMe_2_ are from 57 to 95% for series **I** and **II**. In many cases, considering only the largest effects on basicity, we see that first introducing a strong electron-donating group attenuates the effect of a weak donating group more than for the inverse order of introduction. We interpret this observation in terms of a saturation effect of the electron donation, i.e., a large electron donation to the conjugated system hinders the power of a weaker donating group.

The groups N=C(NMe_2_)_2_ and N=P(NMe_2_)_3_ have similar electron-donation strengths, so the order of introduction in the conjugated system brings slight differences. If we still consider the strongest effects on basicity, introducing the first N=C(NMe_2_)_2_ leads to an attenuation of 70% and 64% for series **I** and **II**, respectively, and 68% and 65% when starting with N=P(NMe_2_)_3_ as the first substitution. These similar attenuation factors show that the two groups experience a comparable mutual interaction. These observations are consistent with our interpretation in terms of the partial saturation effect of electron donation.

The partial substituent effects in our series of nitriles involve a blend of different contributions: principally, an electronic donor effect, modified by the group position relative to the nitrile functional group and its conformation relative to the cyclopropene plane. When we consider the strongest increases in the first and second substitution in each cycle of Scheme 2, Schemes S4 and S5 (SM), leading to the strongest bases, the substituent effects appear fairly regular and can be interpreted in terms of the partial saturation of the pushing effect in the push–pull system. All detailed partial substituent effects estimated for derivatives studied here and previously are compiled in Table S8 (SM).

### 3.7. Macroscopic Basicities

The discussed above microscopic PA_i_s and GB_i_s (Table S2 in SM) give information on the basicity properties of potential sites of protonation (cyano and imino N atoms) in individual isomers. They are also employed in this work for analysis of the total and partial substituent effects in mono- and disubstituted derivatives containing simple (Me, NH_2_, and NMe_2_) and large groups (N=C(NH_2_)_2_, N=C(NMe_2_)_2_, and N=P(NMe_2_)_3_). These effects encompass the pushing effect transmitted through the methylenecyclopropene and cyclopropenimine parts to the favored site of protonation as well as all intramolecular interactions between the substituents and other groups in the π-electron systems. On the other hand, the macroscopic PA_m_s and GB_m_s can inform about the observable gas-phase basicity properties of the isomeric mixture at the temperature of 298 K, and can be compared with those of other N-bases. For their estimations, we considered only those isomers of the neutral and protonated forms that can significantly contribute in the isomeric mixture, i.e., for which the relative Gibbs energies are not higher than 20 kJ mol^−1^.

For nitriles containing one simple substituent Me, NH_2_, and NMe_2_, the relative Gibbs energies (Δ*G* < 6 kJ mol^−1^) of individual isomers for both the neutral and C≡NH^+^ forms are not very high (Figure S1 in SM). Assuming that geometrical isomerism has no high energy barrier in the conjugated system, particularly for cyano-N-protonated forms (Figure S5 and Table S5 in SM), we considered the isomers **A** and **B** in the isomeric mixtures of neutral and protonated derivatives **I.2-I.4** and **II.2-II.4**. The imino N(Z)-protonated forms of **II.2-II.4** are neglected since they have considerably higher *G*s than the corresponding C≡NH^+^ forms (Δ*G* 40–50 kJ mol^−1^). The same is true for the protonation of the amino N atom. We showed previously that the *syn*- and *anti*-amino N-protonation in **I.13** and **II.13** require exceptionally more energy (*ca*. 200 kJ mol^−1^) than the cyano one [9]. Taking into account the relative Gibbs energies between the isomers **A** and **B**, we calculated first the isomeric equilibrium constants and percentage contents of the two isomers in the neutral and protonated isomeric mixtures, and next the macroscopic basicity parameters PA_m_ and GB_m_ for each derivative with a simple substituent according to Equations (S4)–(S8) given in the SM. The estimated percentage contents of **A** and **B**, PA_m_s and GB_m_s for the isomeric mixtures are summarized in Table 1. The scheme of the favored acid-base equilibria is also included in this table. The estimated values of the macroscopic basicity parameters are intermediate between those found for individual isomers (Table S2 in SM).

Although four isomers are possible in the isomeric mixtures of the neutral forms of **I.5-I.10** and **II.5-II.10** containing one large substituent (N=C(NH_2_)_2_, N=C(NMe_2_)_2_, and N=P(NMe_2_)_3_), not all of them contribute significantly to the basicity properties. The relative Gibbs energies for several isomers are higher than 20 kJ mol^−1^ (Figure S2), and their contribution to the isomeric mixtures can be neglected. On the other hand, all four isomers have to be considered in the isomeric mixtures of the protonated forms. Their Δ*G*s are smaller than 20 kJ mol^−1^. The isomer **Aa** of the monocation protonated at the imino N atom in the N=C(NMe_2_)_2_ and N=P(NMe_2_)_3_ group of nitriles in series **I** (Δ*G*s ≤ 20 kJ mol^−1^) has also to be taken into account. The other monoprotonated isomers (Δ*G*s > 20 kJ mol^−1^) can be neglected in their isomeric mixtures. Calculations, performed according to Equations (S4)–(S8) included in the SM for the neutral and protonated isomeric mixtures of **I.5-I.10** and **II.5-II.10**, give the possibility to estimate the percentage contents of all considered neutral and ionic forms, and next, to calculate the macroscopic basicity parameters PA_m_ and GB_m_ for these nitriles. The estimated parameters together with the scheme of acid-base equilibria are included in Table 2. Isomers that slightly contribute to the isomeric mixture (<0.5%) marginally influence the macroscopic basicity parameters. Similarly to the simple derivatives **I.2-I.4** and **II.2-II.4**, the calculated PA_m_ and GB_m_ values are situated between those of the microscopic values found for individual isomers (Table S2 in SM).

**Scheme 3:**
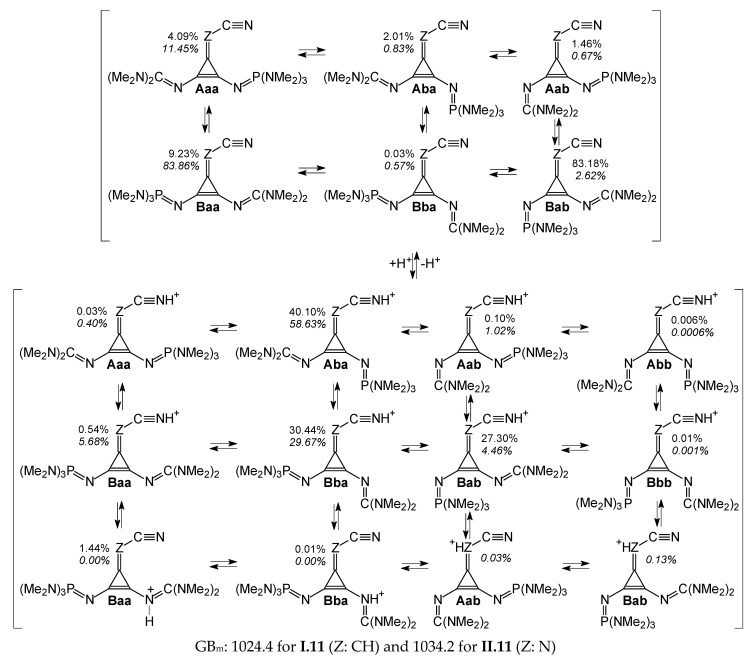
Acid-base equilibria for favored neutral and protonated isomers of nitriles unsymmetrically substituted by two large groups and estimated GB_m_ (in kJ mol^−1^). Percentage contents in normal and italic styles correspond to **I.11** and **II.11**, respectively.

Nitriles unsymmetrically substituted by two different large substituents (**I.11** and **II.11**) are specific cases, for which eight isomers are possible for the neutral and protonated forms (Figure 3). Two structures only (**Abb** and **Bbb**) are found to be stable for the cyano N-protonated forms (Table S1 in SM). For the neutral and other protonated forms, only six isomers (**Aaa**, **Aba**, **Aab**, **Baa**, **Bba**, and **Bab**) are stable and can be present in the isomeric mixtures. Regarding their relative Gibbs energies (Figure S4 in SM), all of them have to be considered in the isomeric mixture of the neutral nitriles. Using Equation (S5) given in SM, we estimated their percentage contents for neutral **I.11** and **II.11** and these are included in Table S9 (SM). For protonated nitriles, the relative Gibbs energies vary from 0 to 72 kJ mol^−1^. Most of the imino N-protonated isomers in the guanidino or phosphazeno substituent and in the C=Z group (with Δ*G*s > 20 kJ mol^−1^) can be neglected in the ionic isomeric mixtures. According to Equation (S6), their percentage contents are very low (see Table S9 in SM). Only the guanidino-protonated isomers **I.11Baa** and **I.11Bba**, and imino N(Z)-protonated isomers **II.11Aab** and **II.11Bab** slightly contribute to the ionic isomeric mixtures. Among the cyano N-protonated isomers, only **I.11Abb**, **II.11Abb**, and **II.11Bbb** can be neglected in these mixtures. Estimating the macroscopic basicity parameters for **I.11** and **II.11**, according to Equations (S7) and (S8) included in the SM, leads to the following PA_m_ and GB_m_ values: 1055.1 and 1024.4, and 1061.7 and 1034.2 kJ mol^−1^, respectively. The favored acid-base equilibria for these two nitriles are summarized in Scheme 3.

**Scheme 4:**
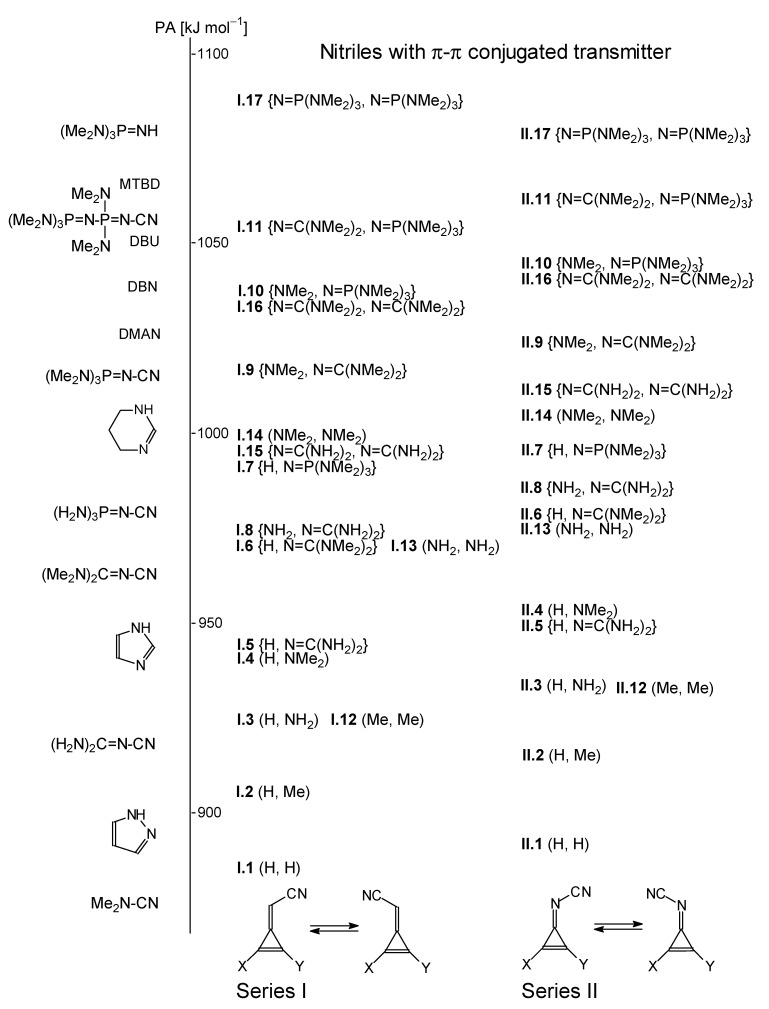
Nitriles with methylenecyclopropene and cyclopropenimine π–π conjugated transmitter on the DFT-calculated PA scale of push–pull N-bases. For selected heterocyclic bases and XCN (**left scale**), PAs are taken from [2,8], respectively. Isomeric phenomena are included in the macroscopic PAs in the case of isomeric nitriles of series **I** and **II** (**right scale**).

Taking into account all macroscopic basicities estimated for series **I** and **II** of the isomeric nitriles studied in this work, as well as in part I, [9], we can see that unsymmetrically and symmetrically substituted derivatives with two strong electron donor substituents such as guanidino and phosphazeno groups ((N=CNMe_2_)_2_ and N=P(NMe_2_)_3_, respectively} possess PAs higher than that of DMAN. Hence, they can be classified as strong bases, also called superbases. Their basicities are close to those of bicyclic N-bases (amidines DBN and DBU, and guanidine MTBD) and of the simplest phosphazene ((Me_2_N)_3_P=NH) [2]. Scheme 4 shows the position of all new nitriles containing the methylenecyclopropene (**I.1-I.17**) and cyclopropenimine (**II.1-II.17**) scaffolds in the general PA scale of superbases.

## 4. Conclusions

When investigating the acid-base properties of polyfunctional compounds by quantum chemical calculations, it is mandatory to take into account all potential sites of protonation/deprotonation and to find the favored one(s). It is also necessary to examine all structural phenomena (e.g., prototropy, rotational and/or geometrical isomerism, intramolecular hydrogen bonds) for both neutral and protonated/deprotonated forms and to determine the composition of the neutral and protonated/deprotonated isomeric mixtures. Possessing this information, it is then possible to identify the preferred acid-base equilibria and to estimate the microscopic and macroscopic acidity-basicity parameters.

Nitriles involved in the push–pull systems described in this work behave as strong bases in the gas phase, often with calculated proton affinities above 1000 kJ mol^−1^. From the DFT results, the N atom of the cyano group is largely favored as the protonation site. The probability of protonation on other nitrogen atoms, which may be foreseen in particular for the imino groups (in the cyclopropenimine or in the substituents) is very low.

For a complete picture of the structural effects on gas-phase basicity, it was necessary to explore the conformational preferences of the substituents. We observed that the protonation of the cyano group exerts, in some cases, a significant effect on the preferred conformations. Not only their position (*syn* or *anti vis-à-vis* C≡N), but also their extreme conformation (*syn* or *anti vis-à-vis* C=Z) affect the gas-phase basicity of specific conformers or configurations.

If we consider the most basic forms in monosubstituted (this work) or in symmetrically disubstituted scaffolds [9], the electron-donor effect order is qualitatively the same: Me < NH_2_ < NMe_2_ < N=C(NH_2_)_2_ < N=C(NMe_2_)_2_ < N=P(NMe_2_)_3_. This order is also analogous to that observed previously for a series of nitriles with an electron donor substituent directly bonded to the cyano group. [7,8]. The substitution of the scaffolds by the two different substituents indicated, which measure the electron-donor effects, could be combined to increase the gas-phase basicity of the nitrile. The results were translated in terms of the non-additivity of the individual effect of each substituent. When proceeding from the unsubstituted nitriles **I.1** (Z: CH) and **II.1** (Z: N) with PAs of about 890 kJ mol^−1^, to the most basic nitriles with PAs just below 1100 kJ mol^−1^, the basicity can be modulated in this 200 kJ mol^−1^ range by playing with the substitution.

## Figures and Tables

**Table 1:** Percentage contents of isomers **A** and **B** in the isomeric mixtures of neutral and protonated nitriles containing simple substituent (% C≡N and % C≡NH^+^) and their macroscopic basicity parameters (PA_m_ and GB_m_ in kJ mol^−1^ at 298.15 K) estimated for the DFT2 structures (see Figure S1 and Table S1 in SM).

Acid-Base Equilibria										
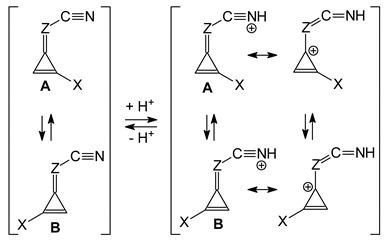										
**X**	**Isomer**	**% C≡N**	**% C≡NH^+^**	**PA_m_**	**GB_m_**	**Isomer**	**% C≡N**	**% C≡NH^+^**	**PA_m_**	**GB_m_**
Me	**I.2A**	63.68	34.87	905.9	875.9	**II.2A**	51.83	41.94	913.8	891.2
	**I.2B**	36.32	65.13			**II.2B**	48.17	58.06		
NH_2_	**I.3A**	85.98	27.65	925.4	892.5	**II.3A**	60.39	11.87	934.4	905.3
	**I.3B**	14.02	72.35			**II.3B**	39.61	88.13		
NMe_2_	**I.4A**	90.01	23.00	943.0	911.0	**II.4A**	69.44	8.97	953.3	924.2
	**I.4B**	9.99	77.00			**II.4B**	30.56	91.03		

**Table 2:** Percentage contents of isomers **Aa**, **Ab**, **Ba**, and **Bb** in the isomeric mixtures of neutral and protonated nitriles containing one large substituent (% C≡N, % C≡NH^+^, and % H^+^N=A) and their macroscopic basicity parameters (PA_m_ and GB_m_ in kJ mol^−1^ at 298.15 K) estimated for the DFT1 or DFT2 structures (see Figures S2 and S3, and Table S1 in SM).

(a) Monosubstituted Derivatives										
Acid-Base Equilibria										
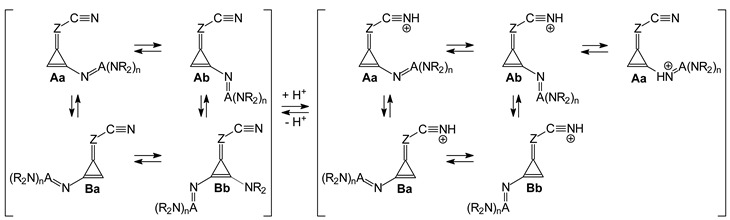										
**Large** **substituent**	**Isomer**	**% C≡N**	**% C≡NH^+^** **(A=NH^+^)**	**PA_m_**	**GB_m_**	**Isomer**	**% C≡N**	**% C≡NH^+^** **(A=NH^+^)**	**PA_m_**	**GB_m_**
	**I.5Aa**	99.85	0.47	943.6	917.1	**II.5Aa**	66.96	0.42	950.4	924.5
N=C(NH_2_)_2_	**I.5Ab**	0.002	66.83			**II.5Ab**	<0.001	24.67		
	**I.5Ba**	0.14	1.95			**II.5Ba**	33.04	70.24		
	**I.5Bb**	0.008	30.75			**II.5Bb**	<0.001	4.67		
	**I.6Aa**	98.03	1.60			**II.6Aa**	87.65	5.16		
		-	(0.37)				-	(<0.001)		
N=C(NMe_2_)_2_	**I.6Ab**	0.08	58.34	971.8	943.9	**II.6Ab**	0.02	48.34	979.0	954.3
	**I.6Ba**	1.70	5.84			**II.6Ba**	12.30	32.91		
	**I.6Bb**	0.19	33.85			**II.6Bb**	0.03	13.59		
	**I.7Aa**	89.48	0.35			**II.7Aa**	56.64	2.09		
		-	(0.20)				-	(<0.001)		
N=P(NMe_2_)_3_	**I.7Ab**	0.61	70.32	990.6	968.2	**II.7Ab**	0.09	58.67	996.2	974.7
	**I.7Ba**	8.89	2.13			**II.7Ba**	43.19	26.37		
	**I.7Bb**	1.02	27.00			**II.7Bb**	0.08	12.87		
**(b) Unsymmetrically Disubstituted Derivatives**										
**Acid-Base Equilibria**										
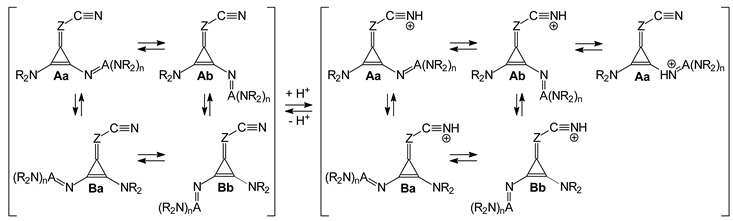										
**Large** **Substituent**	**Isomer**	**% C** **≡N**	**% C** **≡NH^+^** **(A=NH^+^)**	**PA_m_**	**GB_m_**	**Isomer**	**% C** **≡N**	**% C** **≡NH^+^** **(A=NH^+^)**	**PA_m_**	**GB_m_**
	**I.8Aa**	97.89	33.02	973.7	945.0	**II.8Aa**	69.35	3.32	986.3	956.4
N=C(NH_2_)_2_	**I.8Ab**	<0.001	15.45			**II.8Ab**	<0.001	0.67		
	**I.8Ba**	2.11	45.52			**II.8Ba**	30.65	95.97		
	**I.8Bb**	0.005	6.01			**II.8Bb**	<0.001	0.04		
	**I.9Aa**	61.19	46.15			**II.9Aa**	65.66	40.09		
		-	(0.11)				-	(<0.001)		
N=C(NMe_2_)_2_	**I.9Ab**	0.01	5.16	1016.6	985.2	**II.9Ab**	0.001	1.85	1023.9	993.1
	**I.9Ba**	38.64	46.79			**II.9Ba**	34.34	57.96		
	**I.9Bb**	0.16	1.79			**II.9Bb**	0.003	0.10		
	**I.10Aa**	92.05	42.64			**II.10Aa**	12.09	7.22		
		-	(0.01)				-	(<0.001)		
N=P(NMe_2_)_3_	**I.10Ab**	<0.001	0.78	1036.9	1008.1	**II.10Ab**	<0.001	0.11	1045.1	1017.0
	**I.10Ba**	7.90	55.04			**II.10Ba**	87.91	92.63		
	**I.10Bb**	0.05	1.53			**II.10Bb**	0.008	0.03		

## Data Availability

Not applicable.

## Supplementary Materials

The following supporting information can be downloaded at: https://www.mdpi.com/article/10.3390/molecules27144370/s1, the supplementary materials cited in the text are available online.

## Sample Availability

Samples of the compounds are not available from the authors.
