# Supplementary material for: Nitriles with High Gas-Phase Basicity—Part II Transmission of the Push–Pull Effect through Methylenecyclopropene and Cyclopropenimine Scaffolds Intercalated between Different Electron Donor(s) and the Cyano N-Protonation Site
Source: Molecules. 2022 Jul 7;27(14):4370. doi: 10.3390/molecules27144370 (PMC9323925; doi:10.3390/molecules27144370)
Supplement: Supplementary file 1 [file molecules-27-04370-s001.zip › molecules-1795673-supplementary.pdf]

Supplementary Materials

Nitriles with high gas-phase basicity - Part II Transmission of the push-pull effect through methylenecyclopropene and cyclopropenimine scaffolds intercalated between different electron donor(s) and the cyano N-protonation site

Ewa D. Raczyńska,*[a] Jean-François Gal,*[b] Pierre-Charles Maria,[b] Ghulam Sakhi Sakhawat,[c] Mohammad Qasem Fahim,[c] Hamid Saeidian*[d]

[a] Department of Chemistry, Warsaw University of Life Sciences (SGGW), ul. Nowoursynowska 159c, 02-776 Warsaw, Poland

[b] Institut de Chimie de Nice, UMR 7272, Université Côte d'Azur, Parc Valrose, 06108 Nice, France

[c] Department of Chemistry, Science and Research Branch, Islamic Azad University, P.O. Box: 14515-775, Tehran, Iran

[d] Department of Science, Payame Noor University (PNU), P.O. Box: 19395-4697, Teheran, Iran

* Correspondence: ewa_raczynska@sggw.edu.pl (E.D.R.); Jean-Francois.GAL@univ-cotedazur.fr (J.-F.G.); saeidian1980@pnu.ac.ir; saeidian1980@gmail.com (H.S.)

**Contents**	**Page**
Computational details	S2
Selected resonance structures for **I.1** and **II.1** (Scheme S1)	S3
Selected thermochemical data for **I.1-I.17** and **II.1-II.17** (Table S1)	S4
Relative Gibbs energies between two isomers of neutral and protonated forms of monosubstituted nitriles with Me, NH_2, and NMe_2 (Fig. S1).	S8
Relative Gibbs energies between four isomers of neutral and monocationic forms of nitriles with one $N{=}C(NH_2)_2$, $N{=}C(NMe_2)_2$, and $N{=}P(NMe_2)_3$ (Fig. S2)	S9
Relative Gibbs energies between four isomers of neutral and monocationic forms of disubstituted nitriles containing NH_2 and $N{=}C(NH_2)_2$, NMe_2 and $N{=}C(NMe_2)_2$, and NMe_2 and $N{=}P(NMe_2)_3$ (Fig. S3)	S10
Relative Gibbs energies estimated for eight isomers of neutral and monocationic forms of nitriles substituted by $N{=}C(NMe_2)_2$ and $N{=}P(NMe_2)_3$ (Fig. S4)	S11
Microscopic PAs and GBs of the cyano and imino N atoms in investigated nitriles (Table S2)	S13
Z=C and Z−CN bonds lengths in neutral and cyano N-protonated nitriles (Table S3)	S15
C−X and C−Y bonds lengths in neutral and cyano N-protonated nitriles (Table S4)	S16
Energy variations for configurational transformations in selected nitriles (Fig. S5)	S17
Energy barriers for conversion A → B in selected neutral and protonated nitriles (Table S5)	S18
Resonance structures for substituted nitriles (Scheme S2 and Scheme S3)	S19
HOMEDs estimated for selected fragments of neutral and cyano protonated nitriles (Table S6)	S21
HOMAs for cyclopropene ring of selected neutral and cyano protonated nitriles (Table S7)	S22
Scatter plot between HOMED3s for cyano N-protonated forms of nitriles **I** and **II** (Fig. S6)	S23
Intramolecular interactions for nitriles with one or two $N{=}C(NH_2)_2$ (Fig. S7)	S23
Additivity of substituent effects from linear relationships between mono- and disubstituted systems (Fig. S8)	S24
Partial effects of simple substituents for monosubstituted and symmetrically disubstituted nitriles (Scheme S4)	S25
Partial effects of large substituents for monosubstituted and symmetrically disubstituted nitriles (Scheme S5)	S26
Partial substituent effects on PA_is for mono- and disubstituted nitriles (Table S8)	S28
Percentage contents of neutral and protonated isomers and macroscopic basicity parameters for nitriles containing two different large substituents (Table S9)	S29

Computational details

Geometries of selected isomers of neutral, N_{cyano} and N_{imino} protonated nitriles (given in Fig. 1-3) were optimized in their ground states without structural restriction at the DFT1 {B3LYP/6-311+G(d,p)} and/or DFT2 {B3LYP/6-311++G(d,p)} level [10−13]. For calculations, the Gaussian 03 and/or Gaussian 16 programs were employed [14,15]. Structural and energetic parameters such as bonds lengths, energy (*E*), enthalpy (*H*), entropy (*S*), and Gibbs energy (*G*) were calculated at the same DFT1 or DFT2 level for each isomer possessing only real frequencies. Selected thermochemical data are included in Table S1.

Gas-phase basicity parameters such as PA_i (Proton Affinity) and GB_i (Gas-phase Basicity), corresponding to equilibria (S1) for the potential sites of monoprotonation in isomeric nitriles, were determined using equations (S2) and (S3), respectively [17]. In these equations, B_i and B_iH^+ correspond to the analogous isomeric neutral and monoprotonated forms, and their H_i^{298} and G_i^{298} to the enthalpy and Gibbs energy, respectively, calculated at 298 K. For the proton, we applied: $H^{298}(H^+) = 6.2$ kJ mol^{-1} and $G^{298}(H^+) = -26.3$ kJ mol^{-1} [18,19] The microscopic PA_i and GB_i calculated for the cyano and imino N-protonated sites in selected isomers are included in Table S2. For protonation, the imino N atoms in the guanidine and phosphazene X and Y groups as well as the imino N atom in Z were considered. As we showed previously for other push-pull molecules, protonation of the amino N atoms can be neglected for monoprotonation of investigated nitriles [9].

$$B_iH^+ \rightarrow B_i + H^+ \quad (S1)$$
$$PA_i = H_i^{298}(B_i) + H^{298}(H^+) - H_i^{298}(B_iH^+) \quad (S2)$$
$$GB_i = G_i^{298}(B_i) + G^{298}(H^+) - G_i^{298}(B_iH^+) \quad (S3)$$

For estimation of the macroscopic basicity parameters, corresponding to the equilibrium (S4) for the isomeric mixture, the isomer mole-fraction x_i in the neutral isomeric mixture and the isomers mole-fractions y_i in the isomeric mixture of monocation were found on the basis of the relative Gibbs energies (ΔG_i^{298}) calculated for individual isomers of the neutral and monocationic forms using equations (S5) and (S6), respectively. The macroscopic PA_m and GB_m were estimated on the basis of equations (S7) and (S8). Note that contribution of some isomers in the isomeric mixture can be neglected when ΔG_i^{298} is larger than 40 kJ mol^{-1}.

$$y_1B_1H^+ + y_2B_2H^+ + y_3B_3H^+ + \ldots \rightarrow x_1B_1 + x_2B_2 + x_3B_3 + \ldots + H^+ \quad (S4)$$
$$x_i \approx \{\exp(-\Delta G_i^{298}(B_i)/RT)\}/\{\Sigma_1^n[\exp(-\Delta G_i^{298}(B_i)/RT)]\} \quad (S5)$$
$$y_i \approx \{\exp(-\Delta G_i^{298}(B_iH^+)/RT)\}/\{\Sigma_1^n[\exp(-\Delta G_i^{298}(B_iH^+)/RT)]\} \quad (S6)$$
$$PA_m = \Sigma_1^n x_i H_i^{298}(B_i) + H^{298}(H^+) - \Sigma_1^n y_i H_i^{298}(B_iH^+) \quad (S7)$$
$$GB_m = \Sigma_1^n x_i\, G_i^{298}(B_i) + G^{298}(H^+) - \Sigma_1^n y_i\, G_i^{298}(B_iH^+) \quad (S8)$$

Alternation of bonds lengths in selected molecules was measured by the geometry-based HOMA (Harmonic Oscillator Model of Aromaticity) [22] and/or HOMED (Harmonic Oscillator Model of Electron Delocalization) indices [20,21]. Both descriptors are based on the original HOMA idea proposed by Kruszewski and Krygowski [34,35] in the 1970s. The values of HOMA and HOMED were estimated for conjugated fragments of selected neutral and monoprotonated forms according to equation (S9) [20−22,34,35], where n, α, R_o, and R_x are the number of bonds in the considered fragment, the normalization constant (different for different bonds CC and CN), optimum bond length for the reference molecule (also different for different CC and CN bonds), and calculated bond lengths for studied fragment, respectively.

$$\text{HOMA or HOMED} = 1 - \{\Sigma\alpha\Sigma[R_o - R_x]^2\}/n \quad (S9)$$

The HOMA and HOMED procedures are analogous, but their parametrizations (α and R_o) are different. Since there are some discrepancies between the HOMA and HOMED values for heterocompounds due to the use of different reference molecules, and consequently, different zero in the two scales [20-22,34,35], we employed the HOMA index only for the cyclopropene fragment containing three CC bonds, and the HOMED index for fragments containing CC and CN bonds, *i.e.*, cyclopropene (three CC bonds), methylenecyclopropene, (four CC bonds) and cyclopropenimine parts (three CC bonds and one CN bond). For HOMA estimation, we applied the following parameters: α(CC) = 257.7 and R_o(CC) = 1.388, proposed by Krygowski in 1993 for the reformulated HOMA [22]. In the HOMED calculations, the parameters α and R_o for the CC and CN bonds were the same as those already applied for symmetrically disubstituted derivatives: R_o(CC) = 1.3943, R_o(CN) = 1.3342, α(CC) = 72.96 for the cyclopropene ring (three bonds), and α(CC) = 88.09 and α(CN) = 91.6 for the methylenecyclopropene and cyclopropenimine parts (four bonds) [20,21].

Scheme S1. Selected resonance structures for nitriles **I.1** and **II.1**, and HOMEDs estimated for the cyclopropene, methylenecyclopropene, and cyclopropenimine fragments of the neutral and cyano N-protonated forms (HOMED data taken from ref. [9]).

Methylenecyclopropene derivative (**I.1**)

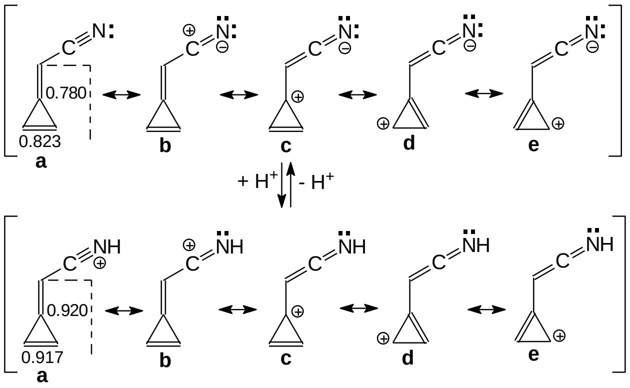

Cyclopropenimine derivative (**II.1**)

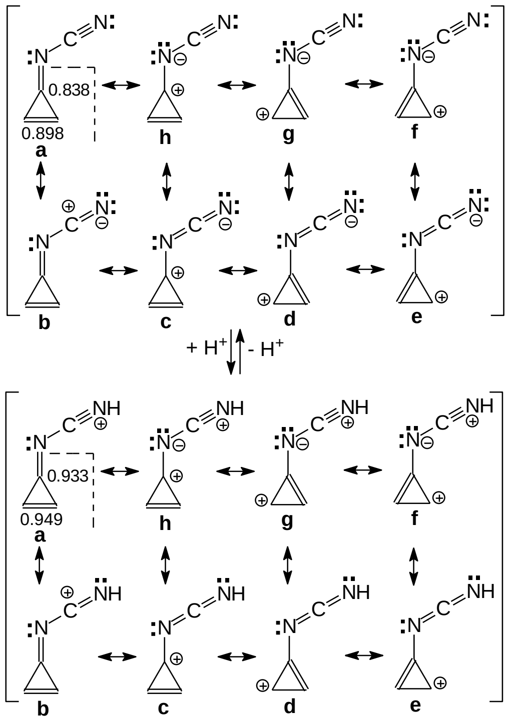

The resonance structures **a-e**, resulting from the pulling C≡N effect, are partially analogous for neutrals and singly protonated forms (labeled monocations in the following) of **I.1** and **II.1**. Localization of π-electrons is the same in the resonance Lewis structures. Nevertheless, their difference results from separation and delocalization of positive and negative charge in the neutral forms. In the case of the monocations, there is no separation of charges. Only positive change is delocalized in parallel to delocalization of π-electrons. This difference in the classical resonance structures seems to dictate stronger electron delocalization for the protonated than neutral forms.

Higher HOMED values, taken from ref. [9], for the neutral and cyano N-protonated forms of **II.1** confirm quantitatively stronger electron delocalization in their cyclopropene and cyclopropenimine parts (Scheme S1). The HOMED values for the cyclopropene part of the monocation of **I.1** and **II.1** are even larger than 0.9, indicating some aromatic character of the ring. Note that HOMED is equal to unity for the reference benzene molecule and equal to zero for hypothetical 1,3,5-cyclohexatriene with localized three single C–C and three double C=C bonds equal to those for ethane and ethene, respectively.

Table S1. Compilation of thermochemical data (at 298 K and 1 atm; *H* and *G* in Hartree) found at the DFT1 and/or DFT2 level for the neutral, N_{cyano} (C≡NH^+) and N_{imino} (ZH^+, $X_{syn}H^+$, and $X_{anti}H^+$) protonated forms of unsubstituted (**1**), unsymmetrically (**2–11**), and symmetrically (**12–17**) substituted nitriles:

(a) parent compounds and derivatives with simple substituents

Compound	Method	*H*(neutral)	*G*(neutral)	*H*(C≡NH^+) [*H*(ZH^+)]	*G*(C≡NH^+) [*G*(ZH^+)]
**I.1** (Z: CH)[a]	DFT1	-246.956008	-246.991320	-247.290894	-247.326809
	DFT2	-246.956054	-246.991366	-247.290970	-247.326883
**I.2A** (Z: CH)	DFT2	-286.265349	-286.304960	-286.607457	-286.648003
**I.2B** (Z: CH)	DFT2	-286.264827	-286.304430	-286.608059	-286.648593
**I.3A** (Z: CH)	DFT1	-302.332570	-302.371418	-302.681712	-302.720472
	DFT2	-302.332649	-302.371523	-302.681821	-302.720580
**I.3B** (Z: CH)	DFT1	-302.330570	-302.369710	-302.682590	-302.721369
	DFT2	-302.330649	-302.369811	-302.682705	-302.721488
**I.4A** (Z: CH)	DFT2	-380.909723	-380.956219	-381.265472	-381.312111
**I.4B** (Z: CH)	DFT2	-380.906870	-380.954143	-381.266500	-381.313252
**I.12** (Z: CH)[a]	DFT1	-325.572510	-325.616525	-325.922299	-325.967769
	DFT2	-325.572587	-325.616576	-325.922411	-325967792
**I.13** (Z: CH)[a]	DFT1	-357.693625	-357.735929	-358.061283	-358.103669
	DFT2	-357.693742	-357.736039	-358.061437	-358.103819
**I.14** (Z: CH)[a]	DFT1	-514.841700	-514.898447	-515.219638	-515.279016
	DFT2	-514.841905	-514.898701	-515.219714	-515.279181
**II.1** (Z: N)[a]	DFT1	-263.012916	-263.047710	-263.350337 [-263.331104]	-263.387202 [-263.366444]
	DFT2	-263.012945	-263.047743	-263.350381 [-263.331172]	-263.387222 [-263.366506]
**II.2A** (Z: N)	DFT2	-302.323085	-302.362350	-302.668268 [-302.652923]	-302.711615 [-302.693160]
**II.2B** (Z: N)	DFT2	-302.323035	-302.362281	-302.669107 [-302.652302]	-302.711922 [-302.692582]
**II.3A** (Z: N)	DFT1	-318.392178	-318.430351	-318.743800 [-318.729678]	-318.783339 [-318.768024]
	DFT2	-318.392247	-318.430416	-318.743889 [-318.729779]	-318.783416 [-318.768127]
**II.3B** (Z: N)	DFT1	-318.391746	-318.429949	-318.745735 [-318.727942]	-318.785183 [-318.766307]
	DFT2	-318.391809	-318.430018	-318.745830 [-318.728037]	-318.785309 [-318.766409]
**II.4A** (Z: N)	DFT2	-396.970684	-397.016415	-397.328919 [-397.317525]	-397.376193 [-397.363611]
**II.4B** (Z: N)	DFT2	-396.969595	-397.015640	-397.331306 [-397.314955]	-397.378381 [-397.361460]
**II.12** (Z: N)[a]	DFT1	-341.631452	-341.675186	-341.984488 [-341.970849]	-342.031421 [-342.016382]
	DFT2	-341.631513	-341.675230	-341.984582 [-341.970949]	-342.031386 [-342.016365]
**II.13** (Z: N)[a]	DFT1	-373.757225	-373.799742	-374.126439 [-374.111096]	-374.168958 [-374.153270]
	DFT2	-373.757327	-373.799849	-374.126554 [-374.111231]	-374.169070 [-374.153413]
**II.14** (Z: N)[a]	DFT1	-530.906978	-530.963795	-531.287205 [-531.273062]	-531.346306 [-531.332109]
	DFT2	-530.907113	-530.963805	-531.287405 [-531.273282]	-531.346555 [-531.332533]

(b) derivatives with large substituents

Compound	Method	H(neutral)<br>G(neutral)	H(C≡NH$^+$)<br>G(C≡NH$^+$)	H(ZH$^+$)<br>G(ZH$^+$)	H[(XorY)$_{syn}$H$^+$]<br>G[(XorY)$_{syn}$H$^+$]	H[(XorY)$_{anti}$H$^+$]<br>G[(XorY)$_{anti}$H$^+$]
**I.5Aa**	DFT1	-451.163907	-451.517427	-	-451.510557	-
		-451.210034	-451.564972		-451.557919	
**I.5Ab**	DFT1	-451.152307	-451.521239	-	-451.498372	-
		-451.199709	-451.569656		-451.547331	
**I.5Ba**	DFT1	-451.157176	-451.518638	-	-	-451.494967
		-451.203800	-451.566319			-451.542276
**I.5Bb**	DFT1	-451.153739	-451.520507	-	-	-451.492566
		-451.201109	-451.568923			-451.540369
**I.6Aa**	DFT2	-608.295279	-608.660318	-	-608.660737	-
		-608.354214	-608.720633		-608.719254	
**I.6Ab**	DFT2	-608.286703	-608.663369	-	-608.653402	-
		-608.347522	-608.724031		-608.713940	
**I.6Ba**	DFT2	-608.290209	-608.661038	-	-	-608.649314
		-608.350384	-608.721858			-608.709233
**I.6Bb**	DFT2	-608.287714	-608.662724	-	-	-608.647606
		-608.348306	-608.723517			-608.707744
**I.7Aa**	DFT2	-1046.109990	-1046.480090	-	-1046.481825	
		-1046.180981	-1046.554560		-1046.554042	
**I.7Ab**	DFT2	-1046.101477	-1046.484357	-	-1046.470753	
		-1046.176266	-1046.559562		-1046.545813	
**I.7Ba**	DFT2	-1046.102701	-1046.481431	-		-1046.471090
		-1046.176627	-1046.556259			-1046.545334
**I.7Bb**	DFT2	-1046.101987	-1046.483906	-		-1046.469103
		-1046.176755	-1046.558950			-1046.543624
**I.8Aa**	DFT1	-506.530212	-506.898702	-	-506.887259	-
		-506.579764	-506.949701		-506.937249	
**I.8Ab**	DFT1	-506.516995	-506.898124	-	-506.873566	-
		-506.567279	-506.948984		-506.925207	
**I.8Ba**	DFT1	-506.525632	-506.898893	-	-	-506.875540
		-506.576140	-506.950004			-506.926837
**I.8Bb**	DFT1	-506.520271	-506.897154	-	-	-506.871393
		-506.570586	-506.948093			-506.923564
**I.9Aa**	DFT2	-742.234396	-742.618990		-742.613145	-
		-742.304707	-742.689963		-742.684209	
**I.9Ab**	DFT2	-742.223980	-742.616024		-742.602674	-
		-742.296736	-742.687894		-742.675428	
**I.9Ba**	DFT2	-742.232461	-742.618397		-	-742.605438
		-742.304273	-742.689976			-742.676900
**I.9Bb**	DFT2	-742.227880	-742.615093		-	-742.600920
		-742.299098	-742.686896			-742.672615
**I.10Aa**	DFT2	-1180.043486	-1180.435953		-1180.429832	-
		-1180.127682	-1180.521409		-1180.513929	
**I.10Ab**	DFT2	-1180.032870	-1180.432990		-1180.418989	-
		-1180.116633	-1180.517630		-1180.504765	
**I.10Ba**	DFT2	-1180.040322	-1180.435748		-	-1180.418842
		-1180.125364	-1180.521650			-1180.503909
**I.10Bb**	DFT2	-1180.036799	-1180.433505		-	-1180.415404
		-1180.120497	-1180.518265			-1180.500099
**I.11Aaa**	DFT2	-1407.425960	-1407.820895	-	-1407.819475	-1407.818406
		-1407.523889	-1407.919957		-1407.916495	-1407.916272
**I.11Aba**	DFT2	-1407.424020	-1407.828064	-	-1407.818675	-1407.809930
		-1407.523218	-1407.926715		-1407.918267	-1407.909484
**I.11Aab**	DFT2	-1407.424634	-1407.822188	-	-1407.811702	-1407.820442
		-1407.522914	-1407.921055		-1407.909303	-1407.918093
**I.11Abb**	DFT2	Not found	-1407.818812	-	Not found	Not found
			-1407.918420			
**I.11Baa**	DFT2	-1407.426654	-1407.824143	-	-1407.825594	-1407.819753
		-1407.524657	-1407.922642		-1407.923572	-1407.917385

**I.11Bba**	DFT2	-1407.419899	-1407.827144	-	-1407.819643	-1407.802257
		-1407.519189	-1407.926455		-1407.919301	-1407.901933
**I.11Bab**	DFT2	-1407.428847	-1407.828568	-	-1407.820566	-1407.817831
		-1407.526733	-1407.926352		-1407.918800	-1407.914785
**I.11Bbb**	DFT2	Not found	-1407.819092	-	Not found	Not found
			-1407.919138			
**I.15a**[a]	DFT1	-655.355945	-655.728858	-	-655.727403	-655.721555
		-655.413245	-655.788816		-655.786470	-655.780619
**I.15b**[a]	DFT1	-655.352295	-655.738191	-	-655.722773	-655.704598
		-655.409862	-655.797981		-655.783394	-655.763926
**I.15c**[a]	DFT1	-655.361188	-655.737068	-	-655.719616	-655.728396
		-655.417898	-655.796735		-655.778314	-655.787684
**I.15d**[a]	DFT1	Not found	-655.730102	-	-655.708945	Not found
			-655.790568		-655.766692	
**I.16a**[a]	DFT2	-969.617721	-970.005058	-	-970.009357	-970.004272
		-969.701982	-970.090719		-970.092939	-970.088082
**I.16b**[a]	DFT2	-969.611949	-970.009607	-	-970.004267	-969.990496
		-969.697565	-970.094753		-970.089223	-970.076450
**I.16c**[a]	DFT2	-969.617762	-970.009821	-	-970.001757	-970.004302
		-969.701862	-970.094180		-970.086340	-970.087922
**I.16d**[a]	DFT2	Not found	-970.000489	-	Not found	Not found
			-970.087512			
**I.16a**[a]	DFT2	-1845.233382	-1845.637109	-	-1845.634908	-1845.633388
		-1845.345316	-1845.750630		-1845.746131	-1845.743233
**I.17b**[a]	DFT2	-1845.230255	-1845.642336	-	-1845.633537	-1845.620084
		-1845.342696	-1845.755167		-1845.748029	-1845.732928
**I.17c**[a]	DFT2	-1845.233872	-1845.639229	-	-1845.628697	-1845.633858
		-1845.345395	-1845.751551		-1845.739963	-1845.745613
**I.17d**[a]	DFT2	Not found	-1845.645442	-	Not found	Not found
			-1845.758830			
**II.5Aa**	DFT1	-467.226711	-467.580951	-467.575619	-467.563835	-
		-467.272374	-467.629836	-467.622777	-467.610069	
**II.5Ab**	DFT1	-467.212220	-467.584772	-467.573624	-467.547313	-
		-467.259340	-467.633682	-467.621333	-467.594729	
**II.5Ba**	DFT1	-467.225737	-467.586681	-467.570566	-	-467.560387
		-467.271707	-467.634670	-467.617865		-467.606291
**II.5Bb**	DFT1	-467.212881	-467.583155	-467.574650	-	-467.545568
		-467.260043	-467.632111	-467.622359		-467.592797
**II.6Aa**	DFT2	-624.357533	-624.725915	-624.720931	-624.714586	-
		-624.415762	-624.787298	-624.780893	-624.772442	
**II.6Ab**	DFT2	-624.347722	-624.728054	-624.718792	-624.704698	-
		-624.407948	-624.789411	-624.779447	-624.764449	
**II.6Ba**	DFT2	-624.354445	-624.727826	-624.715738	-	-624.709374
		-624.413908	-624.789048	-624.776500		-624.768503
**II.6Bb**	DFT2	-624.348159	-624.726529	-624.719721	-	-624.702713
		-624.408168	-624.788213	-624.780225		-624.762322
**II.7Aa**	DFT2	-1062.173741	-1062.547577	-1062.544367	-1062.537111	-
		-1062.244125	-1062.622579	-1062.616703	-1062.609099	
**II.7Ab**	DFT2	-1062.163538	-1062.549979	-1062.542086	-1062.523543	-
		-1062.238049	-1062.625726	-1062.616931	-1062.597771	
**II.7Ba**	DFT2	-1062.171516	-1062.550345	-1062.538318	-	-1062.531469
		-1062.243869	-1062.624971	-1062.614166		-1062.605076
**II.7Bb**	DFT2	-1062.163629	-1062.548590	-1062.543080	-	-1062.525508
		-1062.237935	-1062.624294	-1062.617397		-1062.599617
**II.8Aa**	DFT1	-522.596145	-522.965785	-522.956905	-522.944759	-
		-522.646108	-523.017122	-523.007657	-522.993898	
**II.8Ab**	DFT1	-522.580303	-522.964510	-522.950515	-522.927028	-
		-522.630239	-523.015602	-523.001227	-522.977759	
**II.8Ba**	DFT1	-522.594809	-522.969177	-522.953037	-	-522.941160
		-522.645337	-523.020297	-523.003650		-522.991335
**II.8Bb**	DFT1	-522.581415	-522.961671	-522.953441	-	-522.925599
		-522.631348	-523.012978.	-523.004088		-522.976295

**II.9Aa**	DFT1	-758.300813<br>-758.371328	-758.687927<br>-758.759248	-758.678501<br>-758.749812	-758.672398<br>-758.742088	-
**II.9Ab**	DFT1	-758.289581<br>-758.361243	-758.684599<br>-758.756343	-758.671236<br>-758.743421	-758.659623<br>-758.731016	-
**II.9Ba**	DFT1	-758.299289<br>-758.370716	-758.688026<br>-758.759596	-758.675998<br>-758.747667	-	-758.667895<br>-758.738651
**II.9Bb**	DFT1	-758.291085<br>-758.362108	-758.681361<br>-758.753570	-758.674776<br>-758.746229	-	-758.658657<br>-758.729487
**II.10Aa**	DFT2	-1196.110053<br>-1196.193975	-1196.505224<br>-1196.590789	-1196.495567<br>-1196.580872	-1196.491006<br>-1196.574651	-
**II.10Ab**	DFT2	-1196.099340<br>-1196.182521	-1196.502441<br>-1196.586845	-1196.490113<br>-1196.575379	-1196.477404<br>-1196.562351	-
**II.10Ba**	DFT2	-1196.111832<br>-1196.195848	-1196.507472<br>-1196.593198	-1196.496666<br>-1196.582777	-	-1196.485219<br>-1196.569551
**II.10Bb**	DFT2	-1196.101302<br>-1196.184924	-1196.500733<br>-1196.585729	-1196.493415<br>-1196.578089	-	-1196.475580<br>-1196.559724
**II.11Aaa**	DFT2	-1423.495022<br>-1423.592265	-1423.893744<br>-1423.993011	-1423.886624<br>-1423.985076	-1423.882718<br>-1423.979035	-1423.885419<br>-1423.982039
**II.11Aba**	DFT2	-1423.491465<br>-1423.589782	-1423.899242<br>-1423.997727	-1423.888693<br>-1423.987841	-1423.880091<br>-1423.978548	-1423.874304<br>-1423.973281
**II.11Aab**	DFT2	-1423.492062<br>-1423.589589	-1423.894905<br>-1423.993903	-1423.892502<br>-1423.990702	-1423.871026<br>-1423.970347	-1423.882330<br>-1423.979360
**II.11Abb**	DFT2	Not found	-1423.886675<br>-1423.986966	Not found	Not found	Not found
**II.11Baa**	DFT2	-1423.497525<br>-1423.594145	-1423.896386<br>-1423.995523	-1423.889555<br>-1423.988238	-1423.890406<br>-1423.987024	-1423.883136<br>-1423.979329
**II.11Bba**	DFT2	-1423.491206<br>-1423.589433	-1423.898162<br>-1423.997084	-1423.887759<br>-1423.986637	-1423.883084<br>-1423.980520	-1423.871026<br>-1423.970347
**II.11Bab**	DFT2	-1423.494025<br>-1423.590874	-1423.897062<br>-1423.995295	-1423.893956<br>-1423.991977	-1423.879206<br>-1423.976213	-1423.880071<br>-1423.976616
**II.11Bbb**	DFT2	Not found	-1423.886759<br>-1423.987355	Not found	Not found	Not found
**II.15a**[a]	DFT1	-671.427405<br>-671.483992	-671.800748<br>-671.860934	-671.790152<br>-671.849675	-671.792168<br>-671.850572	-671.790676<br>-671.848730
**II.15b**[a]	DFT1	-671.422738<br>-671.479574	-671.810282<br>-671.869803	-671.793085<br>-671.852192	-671.784715<br>-671.843766	-671.774313<br>-671.832751
**II.15c**[a]	DFT1	-671.425208<br>-671.481323	-671.803408<br>-671.863990	-671.800980<br>-671.859888	-671.776662<br>-671.834819	-671.787492<br>-671.845213
**II.15d**[a]	DFT1	Not found	-671.795555<br>-671.856181	-671.787984<br>-671.847932	-671.761074<br>-671.818583	Not found
**II.16a**[a]	DFT2	-985.684640<br>-985.768210	-986.075929<br>-986.162094	-986.069701<br>-986.154794	-986.071391<br>-986.153923	-986.069339<br>-986.152260
**II.16b**[a]	DFT2	-985.677935<br>-985.762862	-986.079819<br>-986.165080	-986.068583<br>-986.153855	-986.063936<br>-986.147904	-986.053912<br>-986.139073
**II.16c**[a]	DFT2	-985.680505<br>-985.763855	-986.076854<br>-986.161875	-986.074684<br>-986.159012	-986.059250<br>-986.142934	-986.064861<br>-986.147574
**II.16d**[a]	DFT2	Not found	Not found	Not found	Not found	Not found
**II.17a**[a]	DFT2	-1861.307573<br>-1861.418174	-1861.712715<br>-1861.825479	-1861.705910<br>-1861.817036-	-1861.701562<br>-1861.811203	-1861.700179<br>-1861.810006
**II.17b**[a]	DFT2	-1861.302911<br>-1861.413725	-1861.716282<br>-1861.829054	-1861.706907<br>-1861.819151	-1861.697181<br>-1861.809338	-1861.689097<br>-1861.801737
**II.17c**[a]	DFT2	-1861.300972<br>-1861.411608	Not found	-1861.707141<br>-1861.818373	-1861.688641<br>-1861.799286	-1861.695800<br>-1861.807289
**II.17d**[a]	DFT2	Not found	-1861.700549<br>-1861.814683	Not found	Not found	Not found

[a] As in ref. [9].

Figure S1. Relative Gibbs energies (given in parenthesis in kJ mol^{-1} at 298 K) estimated at the DFT1 (*) or DFT2 (**) level for two isomers possible for neutral, cyano and imino N-protonated forms of nitriles containing simple substituent X: Me (**2**), NH_2 (**3**), and NMe_2 (**4**).

Neutral

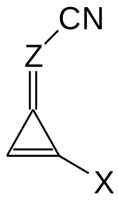

I.2A (**0.0****)
II.2A (**0.0****)
I.3A (**0.0***)
II.3A (**0.0***)
I.4A (**0.0****)
II.4A (**0.0****)

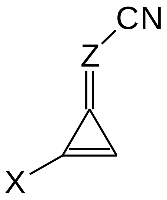

I.2B (1.4**)
II.2B (0.2**)
I.3B (4.5*)
II.3B (1.0*)
I.4B (5.4**)
II.4B (2.0**)

Cyano monocation (C≡NH$^+$)

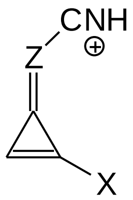

I.2A (1.5**)
II.2A (0.8**)
I.3A (2.4*)
II.3A (4.8*)
I.4A (3.0**)
II.4A (5.7**)

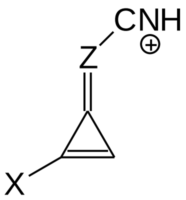

I.2B (**0.0****)
II.2B (**0.0****)
I.3B (**0.0***)
II.3B (**0.0***)
I.4B (**0.0****)
II.4B (**0.0****)

Imino monocation (C=ZH$^+$)

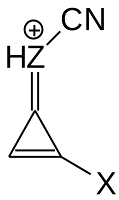

II.2A (49.2**)
II.3A (45.0*)
II.4A (38.8**)

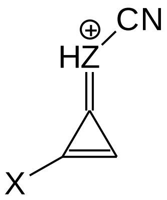

II.2B (50.8**)
II.3B (50.0*)
II.4B (44.4**)

Figure S2. Relative Gibbs energies (given in parentheses in kJ mol^{-1} at 298K) estimated at the DFT1 (*) or DFT2 (**) level for four isomers of neutral and monocationic forms of cyclopropenene monosubstituted derivatives containing one guanidino {Y: C, n = 2, R: H (**I.5** and **II.5**) and Me (**I.6** and **II.6**)} or phosphazeno {Y: P, n = 3, R: Me (**I.7** and **II.7**)} group.

Neutral

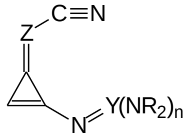

I.5Aa (**0.0***)
II.5Aa (**0.0***)
I.6Aa (**0.0****)
II.6Aa (**0.0****)
I.7Aa (**0.0****)
II.7Aa (**0.0****)

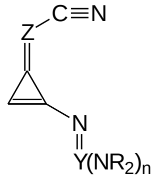

I.5Ab (27.1*)
II.5Ab (34.2*)
I.6Ab (17.6**)
II.6Ab (20.5**)
I.7Ab (12.4**)
II.7Ab (16.0**)

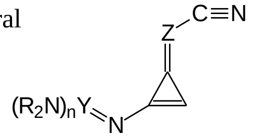

I.5Ba (16.4*)
II.5Ba (1.8*)
I.6Ba (10.1**)
II.6Ba (4.9**)
I.7Ba (11.4**)
II.7Ba (0.7**)

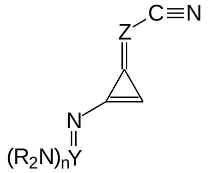

I.5Bb (23.4*)
I.5Bb (32.4*)
I.6Bb (15.5**)
II.6Bb (19.9**)
I.7Bb (11.1**)
II.7Bb (16.2**)

Cyano monocation (C≡NH$^+$)

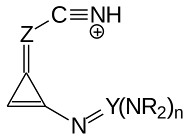

I.5Aa (12.3*)
II.5Aa (12.7*)
I.6Aa (8.9**)
II.6Aa (5.5**)
I.7Aa (13.1**)
II.7Aa (8.3**)

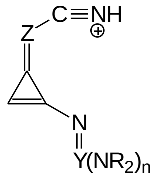

I.5Ab (**0.0***)
II.5Ab (2.6*)
I.6Ab (**0.0****)
II.6Ab (**0.0****)
I.7Ab (**0.0****)
II.7Ab (**0.0****)

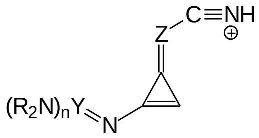

I.5Ba (8.8*)
II.5Ba (**0.0***)
I.6Ba (5.7**)
II.6Ba (1.0**)
I.7Ba (8.7**)
II.7Ba (2.0**)

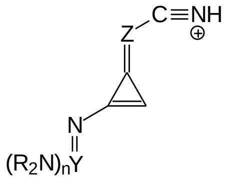

I.5Bb (1.9*)
II.5Bb (6.7*)
I.6Bb (1.3**)
II.6Bb (3.1**)
I.7Bb (1.6**)
II.7Bb (3.8**)

Imino (Z) monocation (C=NH$^+$)

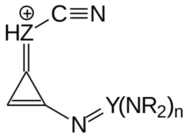

II.5Aa (31.2*)
II.6Aa (22.4**)
II.7Aa (23.7**)

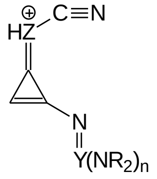

II.5Ab (35.0*)
II.6Ab (26.2**)
II.7Ab (23.1**)

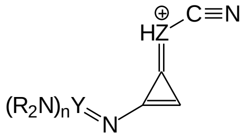

II.5Ba (44.1*)
II.6Ba (33.9**)
II.7Ba (30.4**)

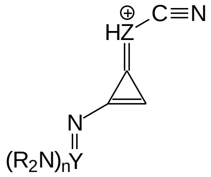

II.5Bb (32.3*)
II.6Bb (24.1**)
II.7Bb (21.9**)

Imino (X) monocation (C=NH$^+$)

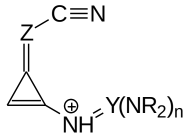

I.5Aa (30.8*)
II.5Aa (64.6*)
I.6Aa (**12.5****)
II.6Aa (44.6**)
I.7Aa (**14.5****)
II.7Aa (43.6**)

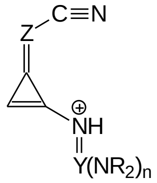

I.5Ab (58.6*)
II.5Ab (104.9*)
I.6Ab (26.5**)
II.6Ab (65.5**)
I.7Ab (36.1**)
II.7Ab (73.4**)

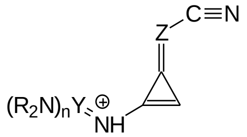

I.5Ba (71.9*)
II.5Ba (74.5*)
I.6Ba (38.5**)
II.6Ba (54.9**)
I.7Ba (37.4**)
II.7Ba (54.2**)

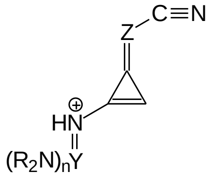

I.5Bb (76.9*)
II.5Bb (109.9*)
I.6Bb (42.8**)
II.6Bb (71.1**)
I.7Bb (41.8**)
II.7Bb (68.5**)

Figure S3. Relative Gibbs energies (given in parentheses in kJ mol^{-1} at 298K) estimated at the DFT1 (*) or DFT2 (**) level for four isomers of neutral and monocationic forms of cyclopropenene disubstituted derivatives containing one NR_2 and one guanidino {Y: C, n = 2, R: H (**I.8** and **II.8**) and Me (**I.9** and **II.9**)} or phosphazeno {Y: P, n = 3, R: Me (**I.10** and **II.10**)} group.

Neutral

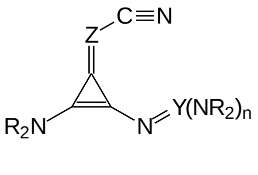

I.8Aa (**0.0***)
II.8Aa (**0.0***)
I.9Aa (**0.0****)
II.9Aa (**0.0****)
I.10Aa (**0.0****)
II.10Aa (4.9**)

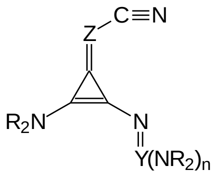

I.8Ab (32.8*)
II.8Ab (41.7*)
I.9Ab (20.9**)
II.9Ab (26.5**)
I.10Ab (29.0**)
II.10Ab (35.0**)

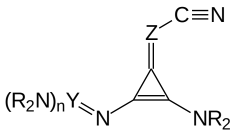

I.8Ba (9.5*)
II.8Ba (2.0*)
I.9Ba (1.1**)
II.9Ba (1.6**)
I.10Ba (6.1**)
II.10Ba (**0.0****)

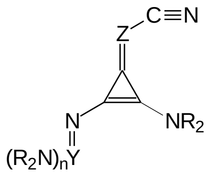

I.8Bb (24.1*)
II.8Bb (38.8*)
I.9Bb (14.7**)
II.9Bb (24.2**)
I.10Bb (18.9**)
II.10Bb (28.7**)

Cyano monocation ($C\equiv NH^+$)

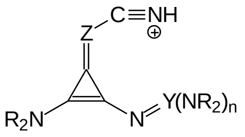

I.8Aa (0.8*)
II.8Aa (8.3*)
I.9Aa (0.03**)
II.9Aa (0.9**)
I.10Aa (0.6**)
II.10Aa (6.3**)

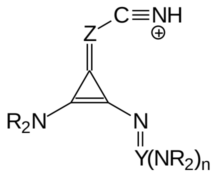

I.8Ab (2.7*)
II.8Ab (12.3*)
I.9Ab (5.5**)
II.9Ab (8.5**)
I.10Ab (10.6**)
II.10Ab (16.7**)

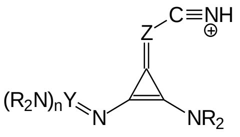

I.8Ba (**0.0***)
II.8Ba (**0.0***)
I.9Ba (**0.0****)
II.9Ba (**0.0****)
I.10Ba (**0.0****)
II.10Ba (**0.0****)

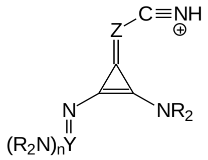

I.8Bb (5.0*)
II.8Bb (19.2*)
I.9Bb (8.1**)
II.9Bb (15.8**)
I.10Bb (8.9**)
II.10Bb (19.6**)

Imino (Z) monocation ($C=NH^+$)

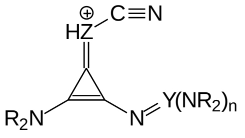

II.8Aa (33.2*)
II.9Aa (25.7**)
II.10Aa (32.4**)

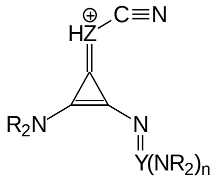

II.8Ab (50.1*)
II.9Ab (42.5**)
II.10Ab (46.8**)

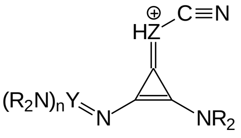

II.8Ba (43.7*)
II.9Ba (31.3**)
II.10Ba (27.4**)

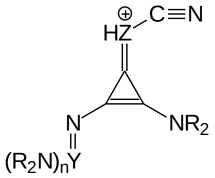

II.8Bb (42.6*)
II.9Bb (35.1**)
II.10Bb (40.0**)

Imino (Y) monocation ($C=NH^+$)

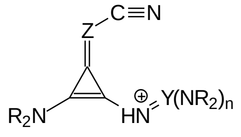

I.8Aa (33.5*)
II.8Aa (69.3*)
I.9Aa (**15.1****)
II.9Aa (46.0**)
I.10Aa (**20.3****)
II.10Aa (48.7**)

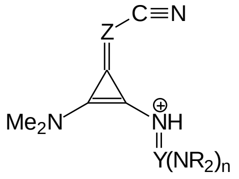

I.8Ab (65.1*)
II.8Ab (111.7*)
I.9Ab (38.2**)
II.9Ab (75.0**)
I.10Ab (44.3**)
II.10Ab (81.0**)

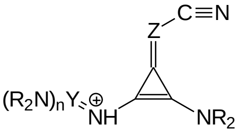

I.8Ba (60.8*)
II.8Ba (76.0*)
I.9Ba (34.3**)
II.9Ba (55.0**)
I.10Ba (46.6**)
II.10Ba (62.1**)

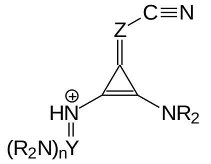

I.8Bb (69.4*)
II.Bab (115.5*)
I.9Bb (45.6**)
II.9Bb (70.0**)
I.10Bb (56.6**)
II.10Bb (87.9**)

Figure S4. Relative Gibbs energies (given in parentheses in kJ mol^{-1} at 298K) estimated at the DFT2 level for eight isomers of neutral and monocationic forms of cyclopropenene disubstituted derivatives (**I.11** and **II.11**) containing one guanidino (Y: C, n = 2, R: Me) and one phosphazeno (Y: P, n = 3, R: Me) group (NF – structure not found).

Neutral

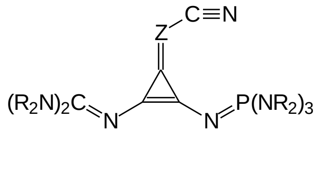

I.11Aaa (7.5)
II.11Aaa (4.9)

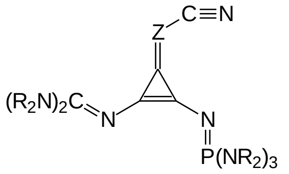

I.11Aba (9.2)
II.11Aba (11.4)

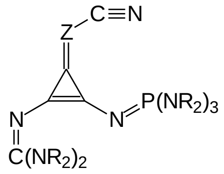

I.11Aab (10.0)
II.11Aab (12.0)

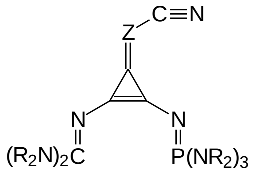

I.11Abb (NF)
II.11Abb (NF)

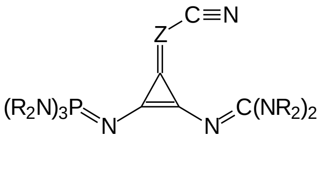

I.11Baa (5.4)
II.11Baa (**0.0**)

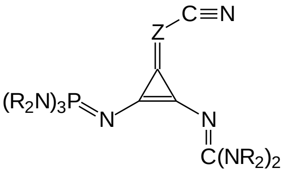

I.11Bba (19.8)
II.11Bba (12.4)

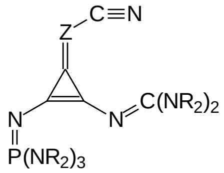

I.11Bab (**0.0**)
II.11Bab (8.6)

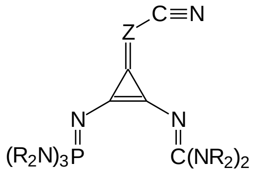

I.11Bbb (NF)
II.11Bbb (NF)

Cyano monocation (C≡NH$^+$)

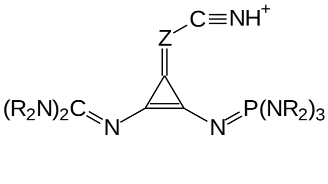

I.11Aaa (17.7)
II.11Aaa (12.4)

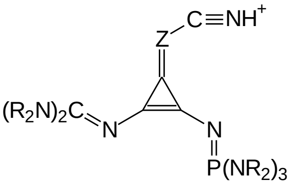

I.11Aba (**0.0**)
II.11Aba (**0.0**)

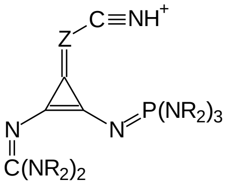

I.11Aab (14.9)
II.11Aab (10.0)

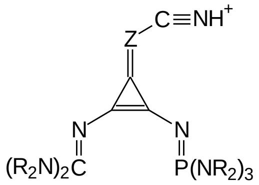

I.11Abb (21.8)
II.11Abb (28.2)

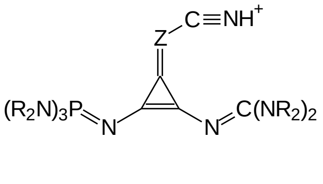

I.11Baa (10.7)
II.11Baa (5.8)

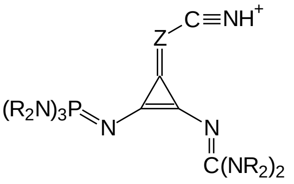

I.11Bba (0.7)
II.11Bba (1.7)

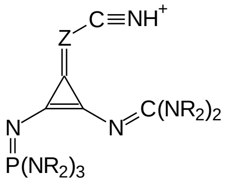

I.11Bab (1.0)
II.11Bab (6.4)

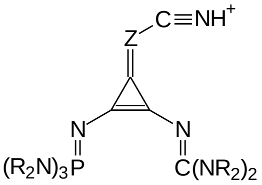

I.11Bbb (19.9)
II.11Bbb (27.2)

Imino (Z) monocation (C=NH$^+$)

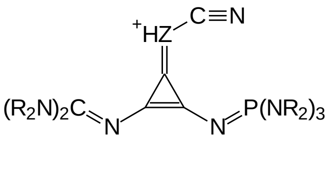

II.11Aaa (33.2)

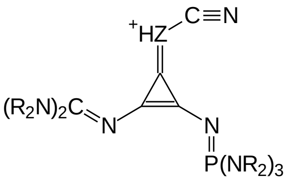

II.11Aba (26.0)

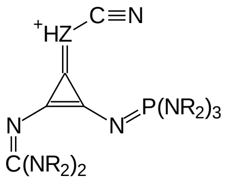

II.11Aab (**18.4**)

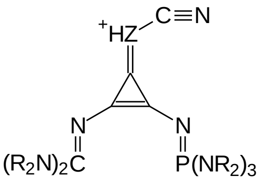

II.11Abb (NF)

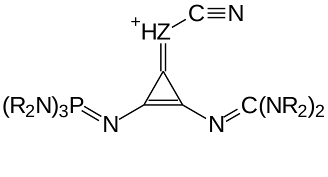

II.11Baa (24.9)

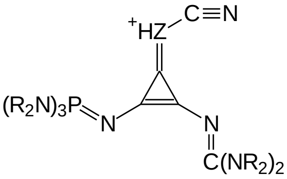

II.11Bba (29.1)

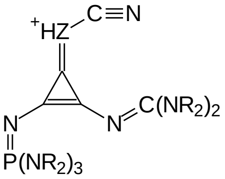

II.11Bab (**15.1**)

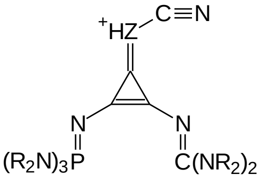

II.11Bbb (NF)

Imino (X – guanidino) monocation (C=NH$^+$)

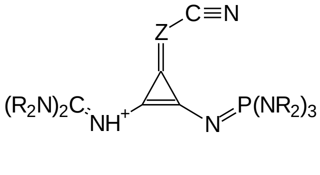

I.11Aaa (27.4)
II.11Aaa (41.2)

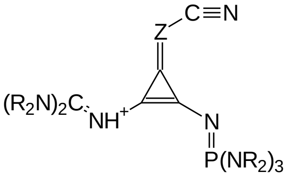

I.11Aba (45.3)
II.11Aba (64.2)

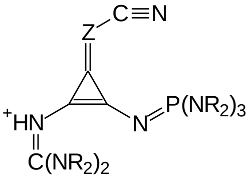

I.11Aab (22.6)
II.11Aab (48.2)

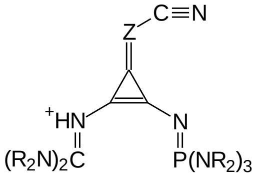

I.11Abb (NF)
II.11Abb (NF)

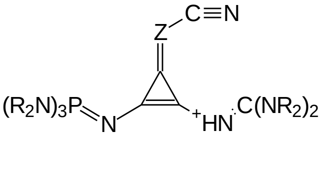

I.11Baa (**8.2**)
II.11Baa (28.1)

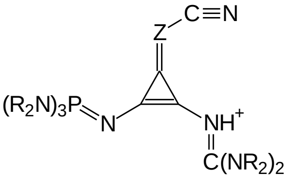

I.11Bba (19.5)
II.11Bba (45.2)

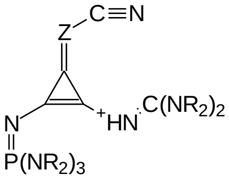

I.11Bab (20.8)
II.11Bab (56.5)

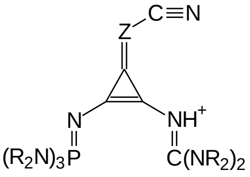

I.11Bbb (NF)
II.11Bbb (NF)

Imino (Y – phosphazeno) monocation (C=NH$^+$)

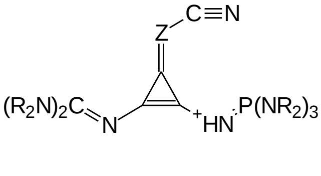

I.11Aaa (26.8)
II.11Aaa (49.1)

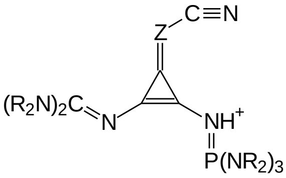

I.11Aba (22.2)
II.11Aba (50.4)

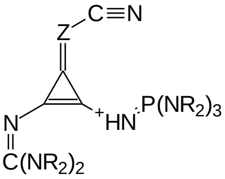

I.11Aab (45.7)
II.11Aab (71.9)

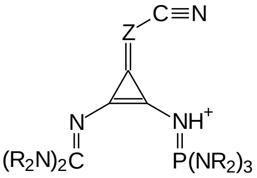

I.11Abb (NF)
II.11Abb (NF)

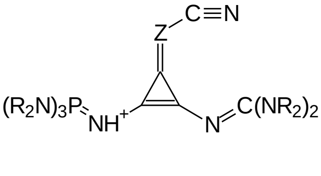

I.11Baa (24.5)
II.11Baa (48.3)

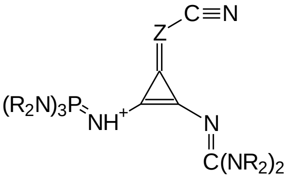

I.11Bba (65.1)
II.11Bba (71.9)

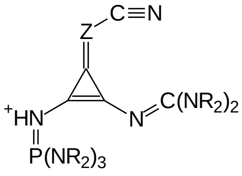

I.11Bab (31.3)
II.11Bab (54.4)

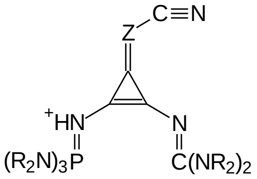

I.11Bbb (NF)
II.11Bbb (NF)

Table S2. Comparison of microscopic PA_i and GB_i (in kJ mol^{-1}) estimated at the DFT1 and/or DFT2 level for cyano and imino N atoms of unsubstituted (**1**), unsymmetrically (**2-11**), and symmetrically (**12-17**) substituted nitriles:

(a) parent compounds and derivatives with simple substituents (Me and NR_2)

Isomer	N_{cyano}		Isomer	N_{cyano}		$N(Z)_{imino}$	
	PA_i	GB_i		PA_i	GB_i	PA_i	GB_i
**I.1**[a]	885.4[b]	854.5[b]	**II.1**[a]	892.1[b]	865.0[b]	841.6[b]	810.5[b]
	885.5[c]	854.6[c]		892.1[c]	865.0[c]	841.7[c]	810.6[c]
**I.2A**	904.4[c]	874.4[c]	**II.2A**	912.5[c]	890.7[c]	872.2[c]	842.2[c]
**I.2B**	907.4[c]	877.3[c]	**II.2B**	914.8[c]	891.7[c]	870.7[c]	840.9[c]
**I.3A**	922.9[b]	890.1[b]	**II.3A**	929.4[b]	900.5[b]	892.3[b]	860.3[b]
	922.9[c]	890.2[c]		929.4[c]	900.5[c]	892.4[c]	860.4[c]
**I.3B**	930.4[b]	897.0[b]	**II.3B**	935.6[b]	906.4[b]	888.9[b]	856.8[b]
	930.5[c]	897.0[c]		935.7[c]	906.5[c]	889.0[c]	856.9[c]
**I.4A**	940.2[c]	908.1[c]	**II.4A**	946.7[c]	918.3[c]	916.8[c]	885.3[c]
**I.4B**	950.4[c]	916.5[c]	**II.4B**	955.9[c]	926.1[c]	912.9[c]	881.6[c]
**I.12**[a]	924.6[b]	895.9[b]	**II.12**[a]	933.1[b]	909.0[b]	897.3[b]	869.5[b]
	924.7[c]	895.8[c]		933.2[c]	908.8[c]	897.4[c]	869.3[c]
**I.13**[a]	971.5[b]	939.2[b]	**II.13**[a]	975.6[b]	943.1[b]	935.3[b]	901.9[b]
	971.6[c]	939.3[c]		975.6[c]	943.1[c]	935.3[c]	902.0[c]
**I.14**[a]	998.5[b]	972.9[b]	**II.14**[a]	1004.5[b]	978.0[b]	967.4[b]	940.7[b]
	991.1[c]	972.7[c]		1004.6[c]	978.6[c]	967.6[c]	941.8[c]

(b) derivatives with large substituents {$N=C(NR_2)_2$, and $N=P(NR_2)_3$}

Isomer	N_{cyano}		$N(Z)_{imino}$		$N(X_{syn})_{imino}$		$N(X_{anti})_{imino}$	
	PA_i	GB_i	PA_i	GB_i	PA_i	GB_i	PA_i	GB_i
**I.5Aa**	934.4[b]	903.6[b]	-	-	916.3[b]	887.1[b]	-	-
**I.5Ab**	974.8[b]	945.0[b]	-	-	914.8[b]	886.4[b]	-	-
**I.5Ba**	955.2[b]	925.5[b]	-	-	-	-	893.1[b]	862.4[b]
**I.5Bb**	969.1[b]	939.4[b]	-	-	-	-	895.8[b]	864.4[b]
**I.6Aa**	964.6[c]	935.7[c]	-	-	965.7[c]	932.1[c]	-	-
**I.6Ab**	995.1[c]	962.3[c]	-	-	969.0[c]	935.7[c]	-	-
**I.6Ba**	979.8[c]	949.0[c]	-	-	-	-	949.0[c]	915.8[c]
**I.6Bb**	990.8[c]	958.8[c]	-	-	-	-	951.1[c]	917.4[c]
**I.7Aa**	977.9[c]	954.5[c]	-	-	982.4[c]	953.2[c]	-	-
**I.7Ab**	1011.4[c]	980.0[c]	-	-	975.7[c]	943.9[c]	-	-
**I.7Ba**	1000.6[c]	970.4[c]	-	-	-	-	973.4[c]	941.7[c]
**I.7Bb**	1008.9[c]	977.2[c]	-	-	-	-	970.1[c]	936.9[c]
**I.8Aa**	973.7[b]	945.0[b]	-	-	943.6[b]	912.3[b]	-	-
**I.8Ab**	1006.8[b]	975.9[b]	-	-	942.4[b]	913.4[b]	-	-
**I.8Ba**	986.2[b]	955.3[b]	-	-	-	-	924.9[b]	894.4[b]
**I.8Bb**	995.7[b]	964.8[b]	-	-	-	-	928.1[b]	900.4[b]
**I.9Aa**	1016.0[c]	985.2[c]	-	-	1000.6[c]	970.1[c]	-	-
**I.9Ab**	1035.5[c]	1000.7[c]	-	-	1000.5[c]	968.0[c]	-	-
**I.9Ba**	1019.5[c]	986.4[c]	-	-	-	-	985.4[c]	952.0[c]
**I.9Bb**	1022.8[c]	991.9[c]	-	-	-	-	985.6[c]	954.4[c]
**I.10Aa**	1036.6[c]	1007.4[c]	-	-	1020.6[c]	987.8[c]	-	-
**I.10Ab**	1056.7[c]	1026.5[c]	-	-	1020.0[c]	992.7[c]	-	-
**I.10Ba**	1044.4[c]	1014.1[c]	-	-	-	-	1000.0[c]	967.6[c]
**I.10Bb**	1047.8[c]	1018.0[c]	-	-	-	-	1000.2[c]	970.3[c]

**I.11Aaa**	1043.1[c]	1013.6[c]	-	-	1039.4[c]	1004.5[c]	1036.6[c]	1003.9[c]
**I.11Aba**	1067.0[c]	1033.1[c]	-	-	1042.4[c]	1010.9[c]	1019.4[c]	987.8[c]
**I.11Aab**	1050.0[c]	1019.0[c]	-	-	1022.4[c]	988.2[c]	1045.4[c]	1011.2[c]
**I.11Baa**	1049.8[c]	1018.6[c]	-	-	1053.6[c]	1021.0[c]	1038.3[c]	1004.8[c]
**I.11Bba**	1075.4[c]	1043.0[c]	-	-	1055.7[c]	1024.2[c]	1010.1[c]	978.6[c]
**I.11Bab**	1055.7[c]	1022.9[c]	-	-	1034.6[c]	1003.1[c]	1027.5[c]	992.5[c]
**I.15a[a]**	985.3[b]	959.8[b]	-	-	981.5[b]	953.6[b]	966.1[b]	938.2[b]
**I.15b[a]**	1019.4[b]	992.7[b]	-	-	978.9[b]	954.4[b]	931.2[b]	903.3[b]
**I.15c[a]**	993.1[b]	968.3[b]	-	-	947.2[b]	952.5[b]	970.3[b]	944.6[b]
**I.16a[a]**	1023.2[c]	994.3[c]	-	-	1034.b[c]	1001.2[c]	1021.1[c]	987.4[c]
**I.16b[a]**	1050.2[c]	1016.5[c]	-	-	1036.2[c]	1002.0[c]	1000.1[c]	968.5[c]
**I.16c[a]**	1035.6[c]	1003.7[c]	-	-	1014.4[c]	983.1[c]	1021.1[c]	987.3[c]
**I.17a[a]**	1066.2[c]	1037.8[c]	-	-	1060.4[c]	1026.0[c]	1056.4[c]	1018.4[c]
**I.17b[a]**	1088.1[c]	1056.6[c]	-	-	1065.0[c]	1037.9[c]	1029.7[c]	998.2[c]
**I.17c[a]**	1070.5[c]	1040.1[c]	-	-	1042.8[c]	1009.6[c]	1056.4[c]	1024.5[c]
**II.5Aa**	936.2[b]	912.2[b]	922.2[b]	893.7[b]	891.3[b]	860.3[b]	-	-
**II.5Ab**	984.3[b]	956.5[b]	955.1[b]	924.1[b]	886.0[b]	854.3[b]	-	-
**II.5Ba**	953.8[b]	926.6[b]	911.5[b]	882.5[b]	-	-	884.8[b]	852.2[b]
**II.5Bb**	978.4[b]	950.6[b]	956.0[b]	925.0[b]	-	-	879.7[b]	847.3[b]
**II.6Aa**	973.4[c]	949.2[c]	960.3[c]	932.4[c]	943.6[c]	910.2[c]	-	-
**II.6Ab**	1004.8[c]	975.2[c]	980.4[c]	949.1[c]	943.4[c]	909.7[c]	-	-
**II.6Ba**	986.5[c]	958.6[c]	954.8[c]	925.7[c]	-	-	938.1[c]	904.7[c]
**II.6Bb**	999.6[c]	971.5[c]	981.7[c]	950.5[c]	-	-	937.1[c]	903.5[c]
**II.7Aa**	987.7[c]	967.3[c]	979.3[c]	951.9[c]	960.2[c]	931.9[c]	-	-
**II.7Ab**	1020.8[c]	991.5[c]	1000.1[c]	968.4[c]	951.4[c]	918.2[c]	-	-
**II.7Ba**	1000.8[c]	974.3[c]	969.2[c]	945.9[c]	-	-	951.2[c]	922.0[c]
**II.7Bb**	1016.9[c]	988.1[c]	1002.4[c]	970.0[c]	-	-	956.3[c]	923.3[c]
**II.8Aa**	976.7[b]	947.8[b]	953.4[b]	922.9[b]	921.5[b]	886.8[b]	-	-
**II.8Ab**	1014.9[b]	985.5[b]	978.2[b]	947.7[b]	916.5[b]	886.1[b]	-	-
**II.8Ba**	989.1[b]	958.2[b]	946.7[b]	914.4[b]	-	-	909.8[b]	879.4[b]
**II.8Bb**	1004.6[b]	975.7[b]	983.0[b]	952.3[b]	-	-	915.5[b]	882.1[b]
**II.9Aa**	1022.6[c]	992.2[c]	997.8[c]	967.4[c]	981.8[c]	947.1[c]	-	-
**II.9Ab**	1043.3[c]	1011.0[c]	1008.2[c]	977.1[c]	977.7[c]	944.5[c]	-	-
**II.9Ba**	1026.8[c]	994.7[c]	995.2[c]	963.4[c]	-	-	974.0[c]	939.7[c]
**II.9Bb**	1030.9[c]	1001.5[c]	1013.6[c]	982.2[c]	-	-	971.3[c]	930.2[c]
**II.10Aa**	1043.7[c]	1015.5[c]	1018.4[c]	989.5[c]	1006.4[c]	973.2[c]	-	-
**II.10Ab**	1064.5[c]	1035.2[c]	1032.2[c]	1005.1[c]	998.8[c]	970.9[c]	-	-
**II.10Ba**	1045.0[c]	1016.9[c]	1016.6[c]	989.6[c]	-	-	986.5[c]	954.8[c]
**II.10Bb**	1054.9[c]	1026.0[c]	1035.7[c]	1006.0[c]	-	-	988.9[c]	957.7[c]
**II.11Aaa**	1053.0[c]	1025.9[c]	1034.3[c]	1005.0[c]	1024.1[c]	989.2[c]	1031.2[c]	997.0[c]
**II.11Aba**	1076.8[c]	1044.8[c]	1049.1[c]	1018.8[c]	1026.5[c]	994.4[c]	1011.3[c]	980.6[c]
**II.11Aab**	1063.9[c]	1035.2[c]	1057.6[c]	1026.8[c]	1001.2[c]	973.4[c]	1030.8[c]	997.0[c]
**II.11Baa**	1053.4[c]	1027.5[c]	1035.5[c]	1008.4[c]	1037.7[c]	1005.2[c]	1018.6[c]	985.0[c]
**II.11Bba**	1074.6[c]	1044.0[c]	1047.3[c]	1016.6[c]	1035.1[c]	1000.5[c]	1003.4[c]	973.8[c]
**II.11Bab**	1064.4[c]	1035.5[c]	1056.2[c]	1026.8[c]	1017.5[c]	985.4[c]	1019.8[c]	986.5[c]
**II.15a[a]**	986.4[b]	963.4[b]	958.6[b]	933.8[b]	963.9[b]	936.2[b]	960.0[b]	931.3[b]
**II.15b[a]**	1023.7[b]	998.2[b]	978.5[b]	952.0[b]	956.6[b]	929.9[b]	929.3[b]	901.0[b]
**II.15c[a]**	999.2[b]	978.4[b]	992.8[b]	967.2[b]	928.9[b]	901.8[b]	957.4[b]	929.1[b]
**II.16a[a]**	1033.5[c]	1007.8[c]	1017.2[c]	988.7[c]	1021.6[c]	986.4[c]	1016.2[c]	982.0[c]
**II.16b[a]**	1061.3[c]	1029.7[c]	1031.8[c]	1000.2[c]	1019.6[c]	984.6[c]	993.3[c]	961.4[c]
**II.16c[a]**	1046.8[c]	1018.7[c]	1041.1[c]	1011.2[c]	1000.6[c]	969.0[c]	1015.3[c]	981.2[c]
**II.17a[a]**	1069.9[c]	1043.1[c]	1052.0[c]	1020.9[c]	1040.6[c]	1005.6[c]	1037.0[c]	1002.4[c]
**II.17b[a]**	1091.5[c]	1064.1[c]	1066.9[c]	1038.1[c]	1041.4[c]	1012.4[c]	1020.1[c]	992.4[c]
**II.17c[a]**	-	-	1072.6[c]	1041.7[c]	1024.0[c]	991.5[c]	10.42.8[c]	1012.6[c]

[a] As in ref. [9]. [b] B3LYP/6-311+G(d,p). [c] B3LYP/6-311++G(d,p).

Table S3. Variations of Z=C and Z−CN bonds lengths (in Å) calculated at the DFT level for selected nitriles when going from the neutral to cyano N-protonated forms.

Compound	Neutral		Cyano N-protonated		Compound	Neutral		Cyano N-protonated	
	Z=C	Z−CN	Z=C	Z−CN		Z=C	Z−CN	Z=C	Z−CN
**I.1**[a]	1.342	1.418	1.379	1.361	**II.1**[a]	1.279	1.335	1.304	1.265
**I.2A**	1.346	1.417	1.388	1.354	**II.2A**	1.284	1.334	1.311	1.260
**I.2B**	1.345	1.417	1.387	1.355	**II.2B**	1.284	1.334	1.311	1.262
**I.3A**	1.352	1.414	1.399	1.346	**II.3A**	1.291	1.331	1.321	1.250
**I.3B**	1.352	1.414	1.398	1.348	**II.3B**	1.289	1.333	1.322	1.256
**I.4A**	1.356	1.412	1.405	1.341	**II.4A**	1.295	1.328	1.328	1.245
**I.4B**	1.354	1.413	1.404	1.345	**II.4B**	1.293	1.331	1.329	1.252
**I.5Aa**	1.360	1.407	1.409	1.339	**II.5Aa**	1.305	1.320	1.331	1.238
**I.5Ab**	1.349	1.415	1.401	1.344	**II.5Ab**	1.289	1.331	1.331	1.249
**I.5Ba**	1.355	1.415	1.407	1.343	**II.5Ba**	1.300	1.332	1.340	1.252
**I.5Bb**	1.350	1.415	1.402	1.343	**II.5Bb**	1.290	1.332	1.330	1.248
**I.6Aa**	1.359	1.410	1.414	1.335	**II.6Aa**	1.302	1.323	1.340	1.235
**I.6Ab**	1.353	1.414	1.406	1.340	**II.6Ab**	1.293	1.330	1.338	1.246
**I.6Ba**	1.356	1.414	1.412	1.340	**II.6Ba**	1.299	1.331	1.337	1.245
**I.6Bb**	1.353	1.414	1.408	1.340	**II.6Bb**	1.294	1.330	1.343	1.247
**I.7Aa**	1.364	1.407	1.418	1.332	**II.7Aa**	1.306	1.319	1.343	1.230
**I.7Ab**	1.355	1.414	1.410	1.338	**II.7Ab**	1.296	1.329	1.342	1.243
**I.7Ba**	1.360	1.412	1.415	1.338	**II.7Ba**	1.304	1.330	1.348	1.245
**I.7Bb**	1.355	1.413	1.418	1.332	**II.7Bb**	1.296	1.329	1.341	1.244
**I.8Aa**	1.370	1.403	1.422	1.332	**II.8Aa**	1.315	1.318	1.348	1.236
**I.8Ab**	1.358	1.412	1.415	1.337	**II.8Ab**	1.297	1.329	1.344	1.243
**I.8Ba**	1.366	1.411	1.420	1.334	**II.8Ba**	1.309	1.327	1.353	1.242
**I.8Bb**	1.359	1.412	1.416	1.334	**II.8Bb**	1.299	1.328	1.343	1.238
**I.9Aa**	1.371	1.405	1.428	1.328	**II.9Aa**	1.314	1.320	1.357	1.231
**I.9Ab**	1.364	1.410	1.422	1.333	**II.9Ab**	1.305	1.327	1.353	1.238
**I.9Ba**	1.369	1.408	1.426	1.330	**II.9Ba**	1.313	1.324	1.357	1.234
**I.9Bb**	1.366	1.408	1.424	1.330	**II.9Bb**	1.307	1.324	1.352	1.234
**I.10Aa**	1.377	1.403	1.431	1.326	**II.10Aa**	1.318	1.314	1.358	1.225
**I.10Ab**	1.366	1.410	1.424	1.331	**II.10Ab**	1.308	1.326	1.356	1.236
**I.10Ba**	1.375	1.406	1.430	1.328	**II.10Ba**	1.320	1.323	1.362	1.233
**I.10Bb**	1.369	1.407	1.426	1.328	**II.10Bb**	1.311	1.322	1.356	1.231

[a] Data taken from ref. [9].

Table S4. Variations of C−X and C−Y bonds lengths (in Å) calculated at the DFT level for selected nitriles substituted by strong electron donor group(s) when going from the neutral to cyano N-protonated forms.

Compound	Neutral		Cyano N-protonated		Compound	Neutral		Cyano N-protonated	
	C−X	C−Y	C−X	C−Y		C−X	C−Y	C−X	C−Y
**I.5Aa**	1.334	-	1.305	-	**II.5Aa**	1.328	-	1.299	-
**I.5Ab**	1.339	-	1.308	-	**II.5Ab**	1.335	-	1.303	-
**I.5Ba**	1.341	-	1.306	-	**II.5Ba**	1.331	-	1.302	-
**I.5Bb**	1.342	-	1.307	-	**II.5Bb**	1.336	-	1.301	-
**I.6Aa**	1.332	-	1.298	-	**II.6Aa**	1.327	-	1.293	-
**I.6Ab**	1.343	-	1.301	-	**II.6Ab**	1.330	-	1.296	-
**I.6Ba**	1.338	-	1.299	-	**II.6Ba**	1.332	-	1.295	-
**I.6Bb**	1.338	-	1.300	-	**II.6Bb**	1.332	-	1.294	-
**I.7Aa**	1.322	-	1.288	-	**II.7Aa**	1.317	-	1.283	-
**I.7Ab**	1.327	-	1.293	-	**II.7Ab**	1.322	-	1.288	-
**I.7Ba**	1.326	-	1.290	-	**II.7Ba**	1.320	-	1.286	-
**I.7Bb**	1.331	-	1.288	-	**II.7Bb**	1.324	-	1.287	-
**I.8Aa**	1.348	1.346	1.322	1.319	**II.8Aa**	1.339	1.342	1.319	1.315
**I.8Ab**	1.357	1.353	1.328	1.322	**II.8Ab**	1.350	1.350	1.324	1.318
**I.8Ba**	1.341	1.354	1.322	1.320	**II.8Ba**	1.338	1.346	1.320	1.313
**I.8Bb**	1.351	1.356	1.328	1.321	**II.8Bb**	1.347	1.352	1.326	1.316
**I.9Aa**	1.357	1.349	1.315	1.317	**II.9Aa**	1.334	1.346	1.312	1.314
**I.9Ab**	1.346	1.354	1.321	1.319	**II.9Ab**	1.339	1.350	1.317	1.314
**I.9Ba**	1.336	1.355	1.316	1.319	**II.9Ba**	1.331	1.351	1.313	1.314
**I.9Bb**	1.342	1.355	1.321	1.318	**II.9Bb**	1.336	1.352	1.318	1.312
**I.10Aa**	1.353	1.336	1.318	1.304	**II.10Aa**	1.343	1.331	1.316	1.300
**I.10Ab**	1.358	1.340	1.324	1.310	**II.10Ab**	1.371	1.338	1.320	1.305
**I.10Ba**	1.347	1.342	1.319	1.306	**II.10Ba**	1.341	1.338	1.316	1.303
**I.10Bb**	1.352	1.346	1.324	1.307	**II.10Bb**	1.344	1.340	1.321	1.301

Restricted rotation about C=C, C=N, and selected C–N bonds

Conformational (*syn/anti*) or configurational (*cis/trans* or *E/Z*) conversions can be treated as rotations about C=C, C=N, and C–N bonds in **I** and **II** or additionally as inversions of nitrogen in iminonitriles **II** [26,27]. Energy barriers for these transformations depend on the nature of isomeric systems [28−30]. All derivatives studied in this work are delocalized to different degrees. Consequently, the C=C and C=N bonds are only partially double and the C–N bonds (C–X and C–Y) are single bonds with a fractional double bond character. The lengthening effects of the C=C and C=N bonds can indicate some decrease of the energy barriers for rotation about these partially double bonds. On the other hand, the shortening effects of the C–N bonds can inform about an increase of the energy barriers for rotation about these single bonds having a partial double bond character. To estimate the energy barrier for restricted rotation about the partially double C=C and C=N bonds (C=Z), first, we considered compounds without substituents X and Y (**I.1** and **II.1**) and we rotated the C≡N group. As it could be expected, we observed a strong decrease of this barrier for the cyano N-protonated forms in comparison to the neutral ones, as well as when proceeding from **I.1** to **II.1** (Fig. S5). Next, we considered the rotation of the C≡N group for some monosubstituted derivatives.

Figure S5. Variations of PM3-estimated energy for configurational (**A** → **B**) transformations in
selected nitriles:

(a) from neutral to cyano N-protonated form of unsubstituted derivatives

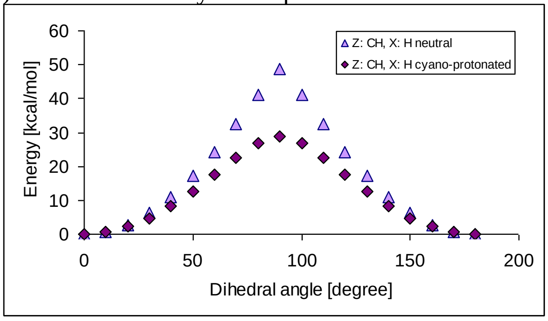

I.1

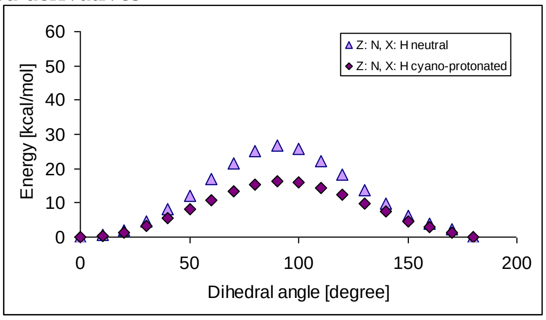

II.1

(b) from cyano N-protonated forms of unsubstituted derivatives to those of monosubstituted ones containing NH_2, *syn*-N=C(NH_2)$_2$, and *syn*-N=P(NH_2)$_3$ group in series **I** and **II**

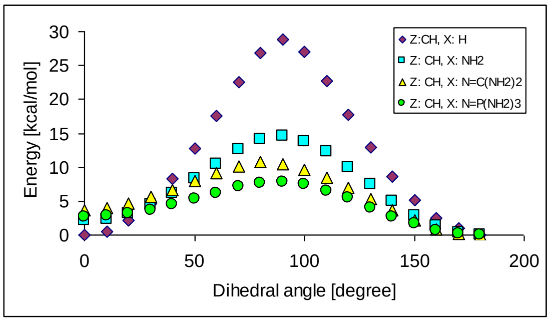

Series **I**

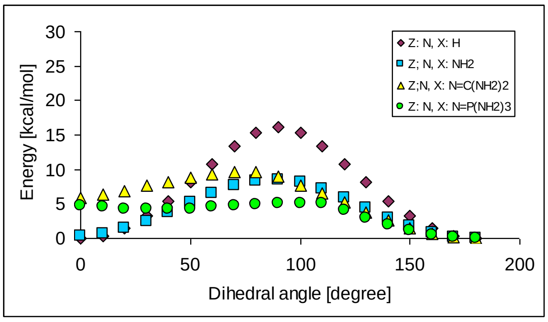

Series **II**

An introduction of one NH_2, $N{=}C(NH_2)_2$, or $N{=}P(NH_2)_3$ group at the cyclopropene ring additionally reduces the energy barrier for C≡N rotation in both the neutral and cyano protonated forms. The barrier seems to be lower for series **II** (Z: N) than for series **I** (Z: CH). Table S5 summarizes the energy barriers for unsubstituted derivatives and for derivatives with one NH_2, $N{=}C(NH_2)_2$, or $N{=}P(NH_2)_3$ in *syn* and *anti* conformation, estimated as $\Delta E = E(90°) - E(0°)$, when proceeding from the isomer **A** to **B**. These simple semi-empirical (PM3)-estimations give some quantitative idea on reduction of the energy barrier after C≡N protonation for derivatives with electron-donating group(s). They also inform about difference between the energy barriers for CH and N derivatives.

Table S5. PM3-estimated energy barriers for isomerism **A** → **B** in selected neutral and protonated nitriles {$\Delta E = E(90°) - E(0°)$, kcal mol^{-1}, 1cal = 4.184 J}

Neutral	Δ*E*(Z=CH)	Δ*E*(Z=N)	Monocation	Δ*E*(Z=CH)	Δ*E*(Z=N)
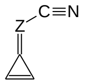	49	27	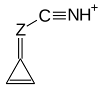	29	16
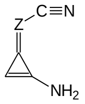	41	22	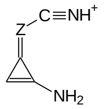	12	8
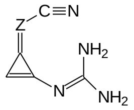	41	23	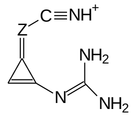	7	1
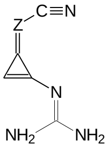	44	25	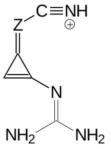	14	8
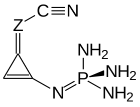	34	19	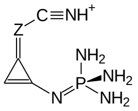	5	-1[a]
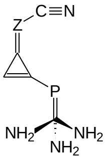	38	19	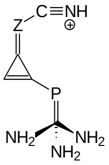	10	6

[a] Negative value is a consequence of very low energy barrier and considerably higher energy of isomer A (dihedral angle 0°) than B (dihedral angle 180°), see in Fig. S5.

Note that analogous PM3-estimations for rotation (**a** → **b**) of $N{=}C(NH_2)_2$ about C(cyclopropane)–N(substituent) lead to the energy barriers ca. 9-11 kcal mol^{-1} for the monocations and ca. 2-4 kcal mol^{-1} for the neutral forms. There are not significant differences between the CH and N derivatives.

Resonance structures for mono- and disubstituted nitriles

Generally, the resonance structures corresponding to the π-π conjugation of the methylenecyclopropene (**b-e**) and cyclopropenimine parts (**b-h**) with the C≡N group in the parent systems (Scheme 1) are analogous for amino substituted derivatives, and their formulae are not included in Scheme S2. To simplify the resonance hybrids for NR_2 derivatives, only additional resonance structures, in which substituent(s) take part in n-π conjugation, are given. For the monosubstituted amino derivatives **I.3**, **I.4**, **II.3**, and **II.4**, one additional resonance structure (**i** or **j** for isomers **A** or **B**, respectively) can be considered, whereas two additional resonance structures **i** and **j** are possible for the disubstituted amino derivatives **I.13**, **I.14**, **II.13**, and **II.14**.

Scheme S2. Selected resonance structures for amino derivatives.

Monosubstituted amino derivatives

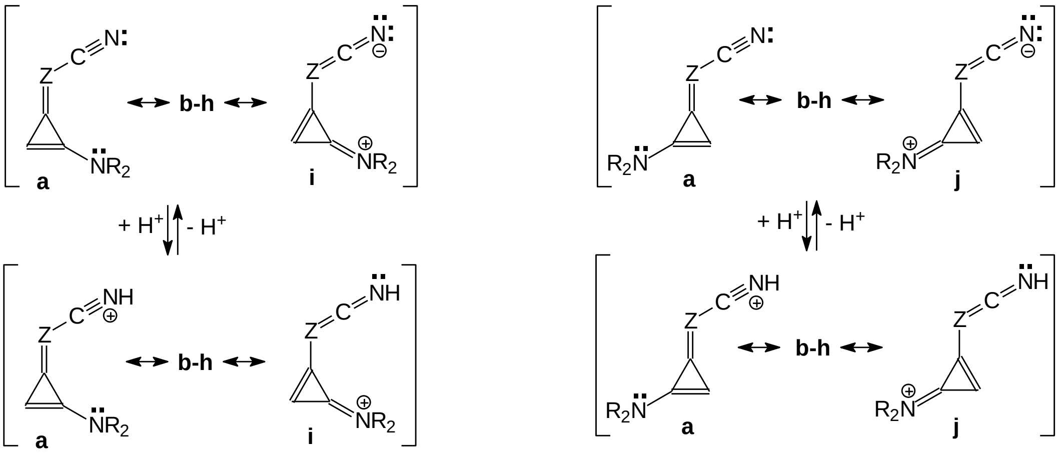

Disubstituted amino derivatives

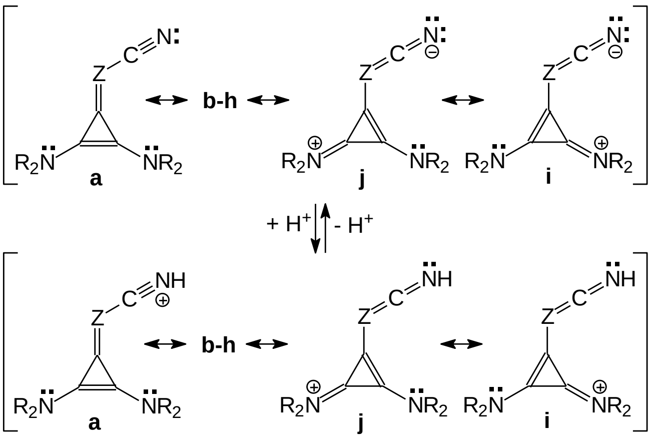

In the guanidino substituent, there are two amino groups cross n-π conjugated with one imino N atom. Hence, two additional resonance structures (**i** and **k**, or **j** and **l** for isomers **A** or **B**, respectively) can be taken into account for the monosubstituted derivatives **I.5**, **I.6**, **II.5**, and **II.6**. In the resonance hybrids of the disubstituted guanidino derivatives **I.15**, **I.16**, **II.15**, and **II.16**, four additional resonance structures (**i-l**) are possible. For simplicity, only disubstituted derivatives are included in Scheme S3. By analogy, three and six additional resonance structures can be written for mono- (**I.7** and **II.7**) and disubstituted (**I.17** and **II.17**) phosphazeno derivatives owing to the presence of three amino groups cross n-π conjugated with one phosphimino N atom in the phosphazeno group.

Scheme S3. Selected resonance structures for disubstituted guanidino derivatives.

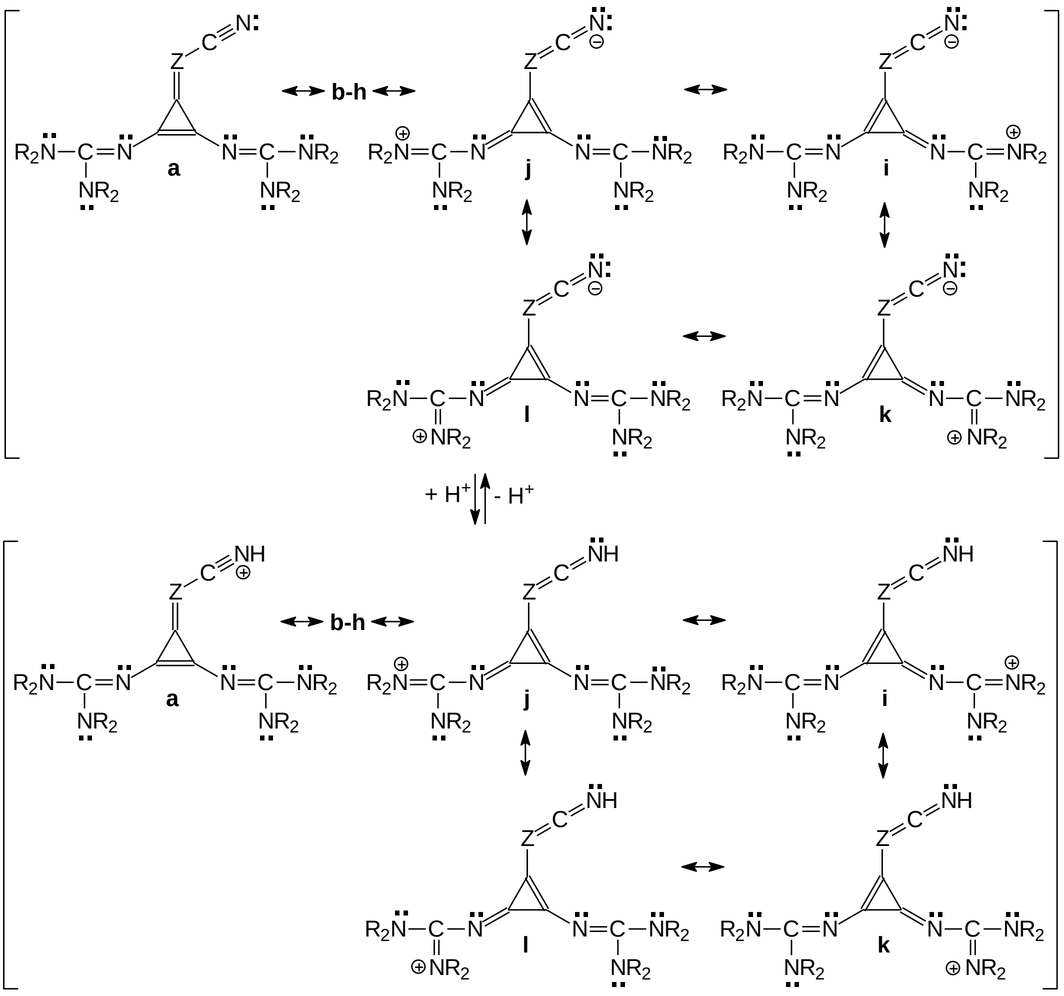

The number of additional resonance structures that can be written for the unsymmetrically disubstituted derivatives **I.8-I.11** and **II.8-II.11**, corresponding to the pushing effect of X and Y is as follows. Three additional resonance structures can be considered for derivatives with the amino and guanidino groups (**I.8-I.9** and **II.8-II.9**), four structures for derivatives with the amino and phosphazeno groups (**I.10** and **II.10**), and five structures for derivatives with the guanidino and phosphazeno groups (**I.11** and **II.11**). Generally, an increase of the number of resonance structures in the resonance hybrid increases electron conjugation in the π-electron system. For investigated compounds, delocalization of electron can increase in the following way: amino < guanidino < phosphazeno derivatives. Some intramolecular interactions between functional groups can destroy this order for favorable or unfavorable conformations.

Table S6. HOMEDs[a] estimated for selected fragments of neutral and cyano N-protonated nitriles.

Isomer	Neutral		C≡NH$^+$		Isomer	Neutral		C≡NH$^+$	
	HOMED3	HOMED4	HOMED3	HOMED4		HOMED3	HOMED4	HOMED3	HOMED4
**I.1**[b]	0.823	0.780	0.917	0.920	**II.1**[b]	0.898	0.838	0.949	0.933
**I.2A**	0.843	0.806	0.939	0.944	**II.2A**	0.912	0.863	0.961	0.952
**I.2B**	0.841	0.803	0.941	0.945	**II.2B**	0.912	0.868	0.968	0.959
**I.3A**	0.908	0.878	0.962	0.965	**II.3A**	0.947	0.908	0.958	0.958
**I.3B**	0.900	0.870	0.964	0.967	**II.3B**	0.956	0.914	0.973	0.975
**I.4A**	0.925	0.900	0.959	0.960	**II.4A**	0.953	0.922	0.942	0.947
**I.4B**	0.918	0.891	0.963	0.965	**II.4B**	0.969	0.932	0.965	0.967
**I.5Aa**	0.872	0.859	0.948	0.948	**II.5Aa**	0.923	0.910	0.934	0.940
**I.5Ab**	0.911	0.875	0.977	0.978	**II.5Ab**	0.958	0.916	0.958	0.962
**I.5Ba**	0.869	0.848	0.952	0.953	**II.5Ba**	0.965	0.941	0.960	0.963
**I.5Bb**	0.914	0.878	0.979	0.979	**II.5Bb**	0.970	0.927	0.972	0.974
**I.6Aa**	0.891	0.874	0.944	0.941	**II.6Aa**	0.931	0.913	0.924	0.930
**I.6Ab**	0.910	0.881	0.970	0.969	**II.6Ab**	0.955	0.920	0.945	0.950
**I.6Ba**	0.885	0.863	0.949	0.947	**II.6Ba**	0.956	0.908	0.953	0.955
**I.6Bb**	0.913	0.883	0.971	0.970	**II.6Bb**	0.969	0.934	0.961	0.965
**I.7Aa**	0.900	0.889	0.934	0.928	**II.7Aa**	0.931	0.919	0.908	0.915
**I.7Ab**	0.918	0.892	0.959	0.958	**II.7Ab**	0.955	0.926	0.929	0.934
**I.7Ba**	0.896	0.879	0.942	0.938	**II.7Ba**	0.963	0.946	0.999	0.941
**I.7Bb**	0.919	0.893	0.934	0.928	**II.7Bb**	0.971	0.941	0.949	0.952
**I.8Aa**	0.924	0.918	0.995	0.978	**II.8Aa**	0.975	0.977	0.986	0.983
**I.8Ab**	0.930	0.907	0.996	0.987	**II.8Ab**	0.977	0.947	0.979	0.979
**I.8Ba**	0.924	0.913	0.995	0.981	**II.8Ba**	0.984	0.971	0.988	0.981
**I.8Bb**	0.940	0.918	0.994	0.984	**II.8Bb**	0.980	0.954	0.980	0.980
**I.9Aa**	0.942	0.936	0.998	0.974	**II.9Aa**	0.985	0.977	0.980	0.970
**I.9Ab**	0.949	0.933	0.991	0.976	**II.9Ab**	0.989	0.970	0.960	0.926
**I.9Ba**	0.945	0.936	0.999	0.976	**II.9Ba**	0.987	0.977	0.982	0.972
**I.9Bb**	0.956	0.942	0.990	0.972	**II.9Bb**	0.987	0.971	0.969	0.955
**I.10Aa**	0.948	0.946	0.993	0.963	**II.10Aa**	0.982	0.978	0.968	0.958
**I.10Ab**	0.956	0.942	0.988	0.970	**II.10Ab**	0.992	0.977	0.950	0.944
**I.10Ba**	0.968	0.962	0.995	0.968	**II.10Ba**	0.994	0.990	0.972	0.957
**I.10Bb**	0.963	0.952	0.986	0.964	**II.10Bb**	0.993	0.982	0.947	0.941
**I.12**[b]	0.860	0.838	0.962	0.965	**II.12**[b]	0.931	0.891	0.981	0.978
**I.13**[b]	0.927	0.911	0.988	0.980	**II.13**[b]	0.973	0.950	0.986	0.987
**I.14**[b]	0.954	0.942	0.994	0.976	**II.14**[b]	0.991	0.971	0.974	0.972
**I.15a**[b]	0.909	0.907	0.999	0.976	**II.15a**[b]	0.980	0.979	0.989	0.973
**I.15b**[b]	0.917	0.901	0.999_8	0.987	**II.15b**[b]	0.982	0.966	0.981	0.972
**I.15c**[b]	0.929	0.919	0.999	0.981	**II.15c**[b]	0.976	0.968	0.984	0.980
**I.16a**[b]	0.923	0.917	0.999_7	0.974	**II.16a**[b]	0.976	0.971	0.984	0.969
**I.16b**[b]	0.932	0.918	0.997	0.978	**II.16b**[b]	0.983	0.968	0.969	0.957
**I.16c**[b]	0.940	0.930	0.994	0.972	**II.16c**[b]	0.976	0.966	0.965	0.955

[a] HOMED3 corresponds to the cyclopropene ring, and HOMED4 refers to the methylenecyclopropene or cyclopropenimine fragment. Both HOMEDs estimated for the DFT-optimized structures. [b] According to data taken from ref. [9].

Table S7. HOMA estimated for the cyclopropene ring in the neutral and cyano N-protonated forms of nitriles containing the methylenecyclopropene (series **I**) and cyclopropenimine transmitters (series **II**).

Isomer	Neutral	C≡NH$^+$	Isomer	Neutral	C≡NH$^+$
**I.1**	0.38	0.75	**II.1**[a]	0.64	0.87
**I.2A**	0.44	0.83	**II.2A**	0.69	0.90
**I.2B**	0.44	0.83	**II.2B**	0.71	0.93
**I.3A**	0.67	0.90	**II.3A**	0.81	0.88
**I.3B**	0.65	0.90	**II.3B**	0.85	0.94
**I.4A**	0.72	0.87	**II.4A**	0.83	0.81
**I.4B**	0.70	0.90	**II.4B**	0.88	0.90
**I.5Aa**	0.52	0.82	**II.5Aa**	0.71	0.77
**I.5Ab**	0.66	0.93	**II.5Ab**	0.83	0.86
**I.5Ba**	0.67	0.84	**II.5Ba**	0.87	0.87
**I.5Bb**	0.67	0.93	**II.5Bb**	0.87	0.90
**I.6Aa**	0.59	0.81	**II.6Aa**	0.74	0.73
**I.6Ab**	0.66	0.90	**II.6Ab**	0.82	0.81
**I.6Ba**	0.58	0.83	**II.6Ba**	0.83	0.84
**I.6Bb**	0.68	0.90	**II.6Bb**	0.88	0.86
**I.7Aa**	0.62	0.76	**II.7Aa**	0.73	0.66
**I.7Ab**	0.69	0.85	**II.7Ab**	0.81	0.74
**I.7Ba**	0.61	0.79	**II.7Ba**	0.86	0.78
**I.7Bb**	0.69	0.76	**II.7Bb**	0.88	0.82
**I.8Aa**	0.72	0.99	**II.8Aa**	0.91	0.95
**I.8Ab**	0.73	0.99_5	**II.8Ab**	0.90	0.93
**I.8Ba**	0.72	0.99	**II.8Ba**	0.94	0.96
**I.8Bb**	0.78	0.99	**II.8Bb**	0.92	0.92
**I.9Aa**	0.78	0.99	**II.9Aa**	0.93	0.92
**I.9Ab**	0.80	0.97	**II.9Ab**	0.95	0.85
**I.9Ba**	0.79	0.99	**II.9Ba**	0.94	0.92
**I.9Bb**	0.83	0.96	**II.9Bb**	0.94	0.83
**I.10Aa**	0.80	0.96	**II.10Aa**	0.92	0.87
**I.10Ab**	0.82	0.95	**II.10Ab**	0.95	0.80
**I.10Ba**	0.81	0.97	**II.10Ba**	0.97	0.97
**I.10Bb**	0.85	0.94	**II.10Bb**	0.96	0.79
**I.11Aaa**	0.73	0.96	**II.11Aaa**	0.88	0.87
**I.11Aba**	0.80	0.95	**II.11Aba**	0.92	0.84
**I.11Aab**	0.77	0.93	**II.11Aab**	0.88	0.79
**I.11Baa**	0.75	0.97	**II.11Baa**	0.92	0.88
**I.11Bba**	0.76	0.95	**II.11Bba**	0.94	0.81
**I.11Bab**	0.77	0.96	**II.11Bab**	0.93	0.83
**I.12**	0.49	0.90	**II.12**	0.75	0.97
**I.13**	0.75	0.99	**II.13**	0.91	0.97
**I.15a**	0.65	0.98	**II.15a**	0.91	0.94
**I.15b**	0.68	0.99	**II.15b**	0.92	0.92
**I.15c**	0.73	0.98	**II.15c**	0.89	0.92

Figure S6. Plot between HOMED3s for cyano N-protonated forms of nitriles **I** and **II**.

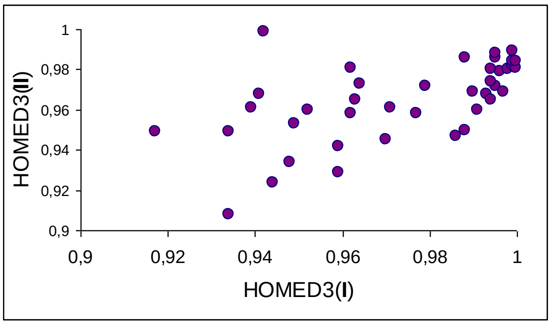

Figure S7. Intramolecular interactions for nitriles containing one or two N=C(NH$_2$)$_2$ groups.

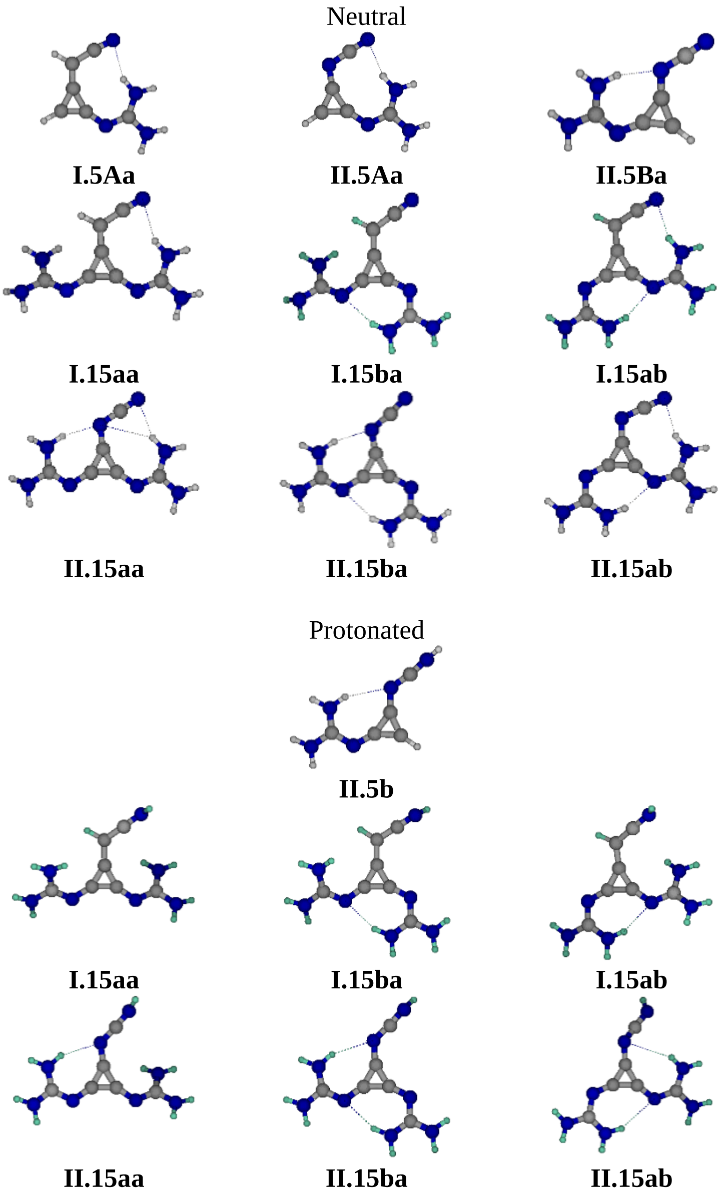

Figure S8. Additivity of substituent effects from linear relationships between mono- and disubstituted systems.

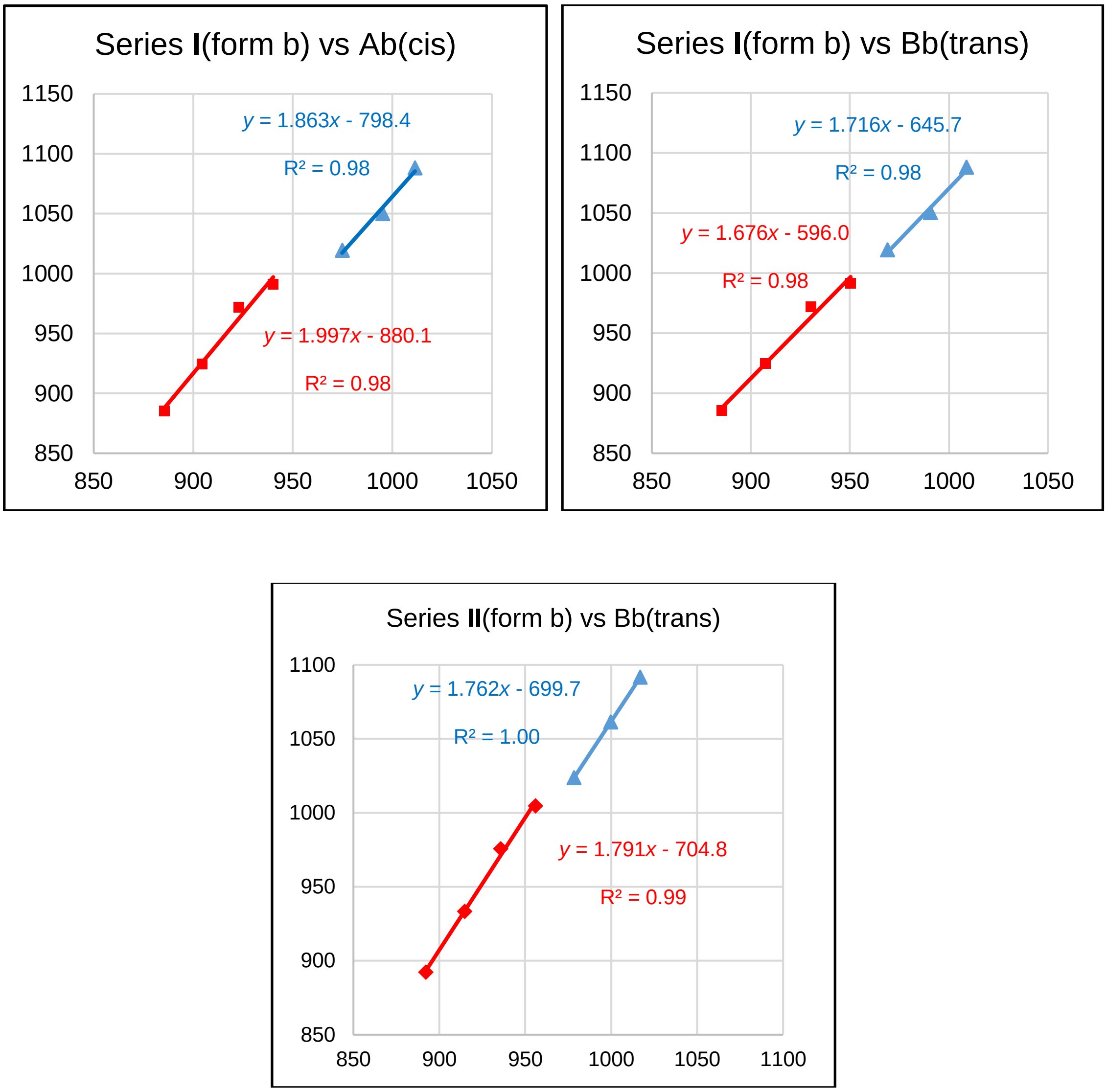

The small substituents (H, Me, NR_2) and the larger ones (N=CNR_2, N=$PNMe_2$) appear to give separate regression lines. The slopes indicate the degree of non-additivity: the slope for a perfect additivity of a correlation of a disubstituted vs a monosubstituted nitrile should be 2; a lower value indicate the degree of non-additivity.

Scheme S4. Partial effects (from Table S2) of simple substituents (Me, NH_2, and NMe_2) on DFT-calculated PA_is of the cyano N atom in monosubstituted nitriles studied here and symmetrically disubstituted derivatives studied previously [9]. The indicated percentages correspond to the attenuation of the effect of the second substitution, as compared to the effect of the first introduced substituent at the same position.

Me effects (DFT2)

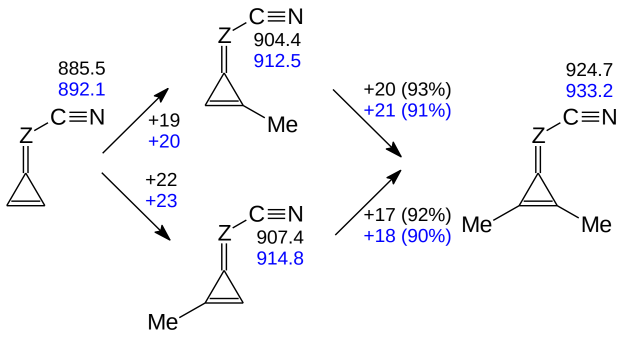

NH$_2$ effects (DFT1)

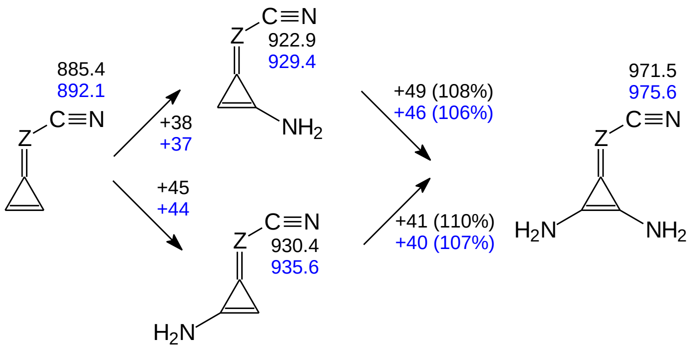

NMe$_2$ effect (DFT2)

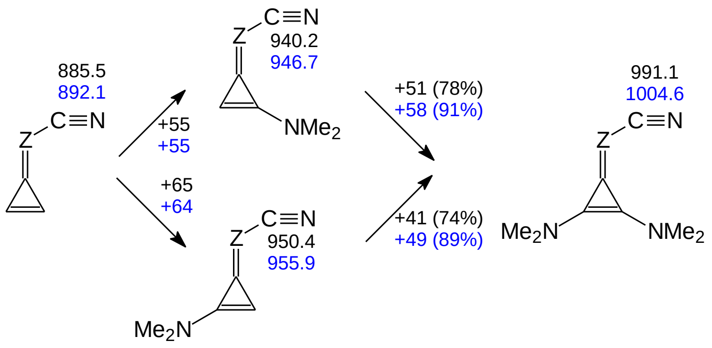

Z: CH (black data) or N (blue data)
PAi in kJ mol^{-1}

Scheme S5. Partial effects (in kJ mol^{-1}) of large substituents {$N{=}C(NH_2)_2$, $N{=}C(NMe_2)_2$, and $N{=}P(NMe_2)_3$} on DFT-calculated PA_is of the cyano N atom in monosubstituted nitriles studied here and symmetrically disubstituted derivatives studied in Part I [9]. The indicated percentages correspond to the attenuation of the effect of the second substitution, as compared to the effect of the first introduced substituent at the same position and, when possible, in the same conformation.

N=C(NH2)2 effects (DFT1)

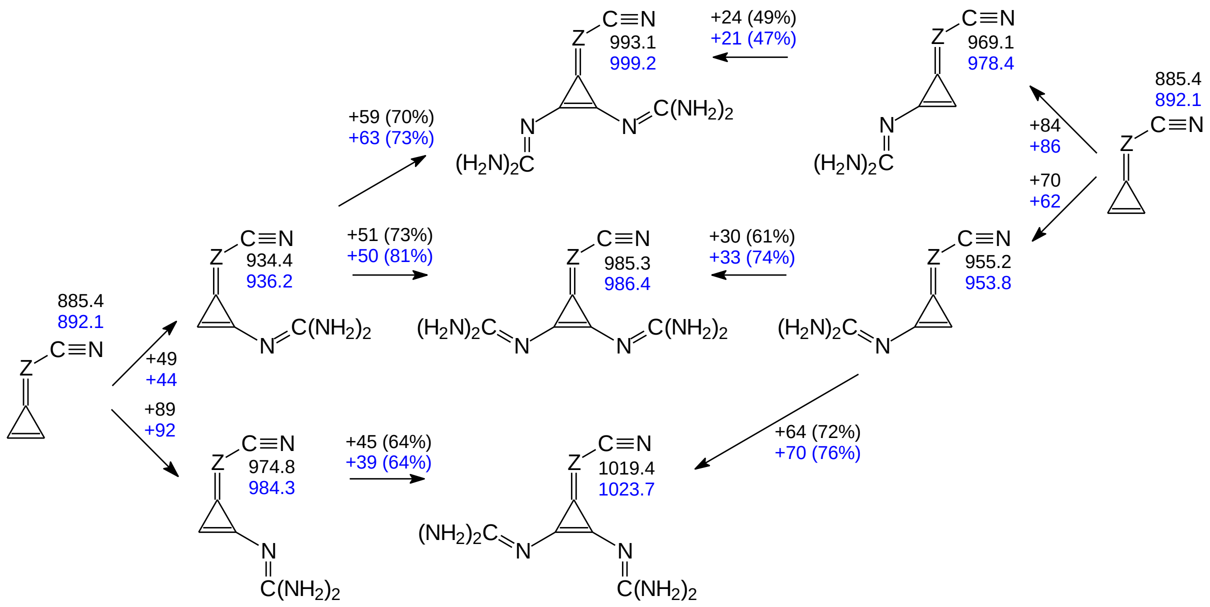

N=C(NMe2)2 effects (DFT2)

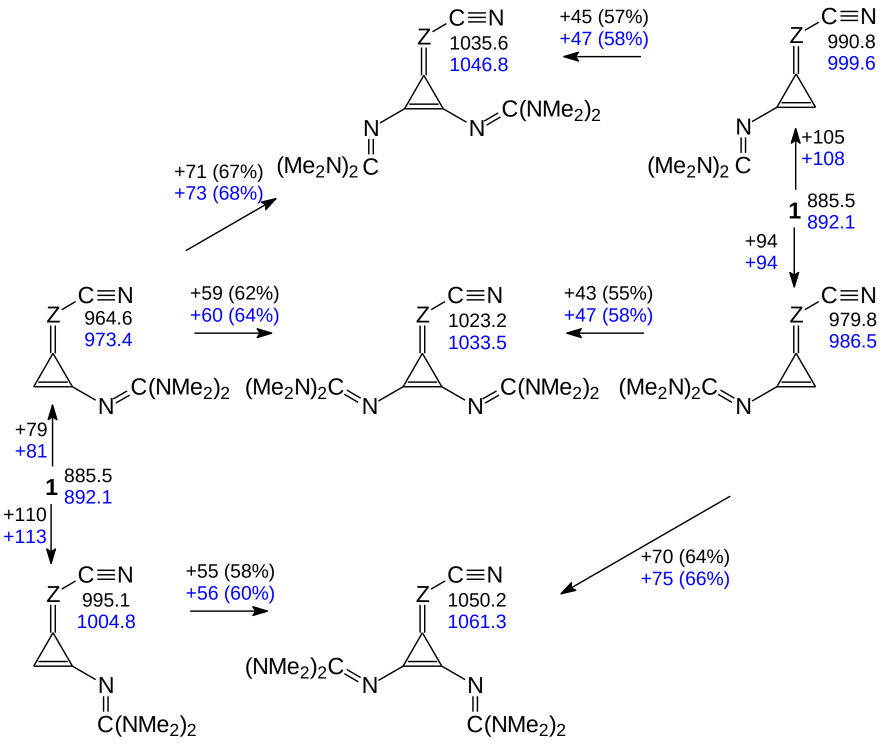

N=P(NMe$_2$)$_3$ effects (DFT2)

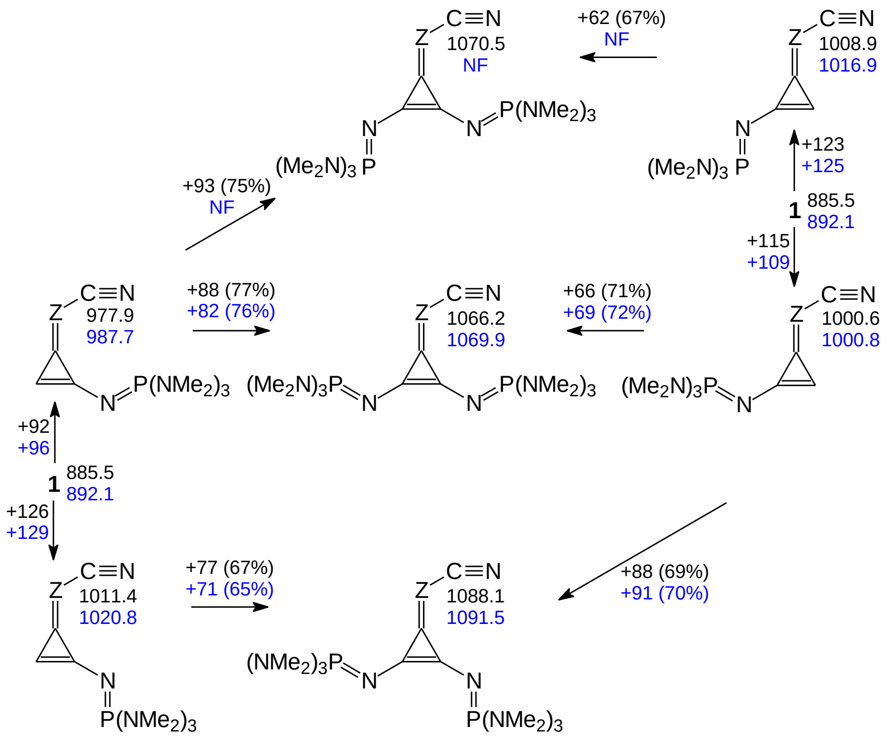

Z: CH (black data) or N (blue data)

PA$_i$ in kJ mol^{-1}

Table S8. Partial substituent effects on PA_is for mono- and disubstituted nitriles

(a) effect of first substituent in monosubstituted nitriles (**I.2-I.7** and **II.2-II.7**)

Isomer	δPA_i (at 298 K in kJ mol^{-1})											
	Me[a]		NH_2[b]		NMe_2[a]		N=C$(NH_2)_2$[b]		N=C$(NMe_2)_2$[a]		N=P$(NMe_2)_3$[a]	
	**I.2**	**II.2**	**I.3**	**II.3**	**I.4**	**II.4**	**I.5**	**II.5**	**I.6**	**II.6**	**I.7**	**II.7**
**Aa**[c]	18.9	20.4	37.5	37.3	54.7	54.6	49.0	44.1	79.1	81.3	92.4	95.6
**Ab**	-	-	-	-	-	-	89.4	92.2	109.6	112.7	125.9	128.7
**Ba**[d]	21.9	22.7	45.0	43.5	64.9	63.8	69.8	61.7	94.3	94.4	115.1	108.7
**Bb**	-	-	-	-	-	-	83.7	86.3	105.3	107.5	123.4	124.8

(b) effect of second substituent in unsymmetrically disubstituted nitriles (**I.8-I.10** and **II.8-II.10**)

Isomer	δPA_i (at 298 K in kJ mol^{-1})											
	NH_2[b]		NMe_2[a]		NMe_2[a]		N=C$(NH_2)_2$[b]		N=C$(NMe_2)_2$[a]		N=P$(NMe_2)_3$[a]	
	**I.8**	**II.8**	**I.9**	**II.9**	**I.10**	**II.10**	**I.8**	**II.8**	**I.9**	**II.9**	**I.10**	**II.10**
**Aa**[c]	31.0	35.3	39.7	40.3	43.8	44.2	43.3	41.1	65.6	66.7	86.2	87.8
**Ab**[c]	26.6	26.2	32.0	31.3	38.9	38.0	76.4	79.3	85.1	87.4	106.3	108.6
**Ba**[d]	39.3	40.5	51.4	49.2	58.7	56.0	63.3	59.7	79.3	80.1	104.2	98.3
**Bb**[d]	32.0	30.6	40.4	38.5	45.3	43.7	72.8	75.2	82.6	84.2	107.6	108.2

(c) effect of second substituent in unsymmetrically disubstituted nitriles (**I.11** and **II.11**)

Isomer	δPA_i (at 298 K in kJ mol^{-1})				Isomer	δPA_i (at 298 K in kJ mol^{-1})			
	N=C$(NMe_2)_2$		N=P$(NMe_2)_3$			N=C$(NMe_2)_2$		N=P$(NMe_2)_3$	
	**I.11**	**II.11**	**I.11**	**II.11**		**I.11**	**II.11**	**I.11**	**II.11**
**Aaa**	65.2	65.3	63.3	66.5	**Baa**	49.2	52.6	85.2	80.0
**Aba**	55.6	56.0	87.2	90.3	**Bba**	74.8	73.8	80.3	69.8
**Aab**	72.1	76.2	59.2	64.3	**Bab**	46.8	47.5	91.1	91.0

(d) effect of second substituent in symmetrically disubstituted nitriles (**I.12-I.17** and **II.12-II.17**)

Isomer	δPA_i (at 298 K in kJ mol^{-1})											
	Me		NH_2		NMe_2		N=C$(NH_2)_2$		N=C$(NMe_2)_2$		N=P$(NMe_2)_3$	
	**I.12**	**II.12**	**I.13**	**II.13**	**I.14**	**II.14**	**I.15**	**II.15**	**I.16**	**II.16**	**I.17**	**II.17**
**Aa**[c]/**a**[e]	17.3	18.4	41.1	40.0	40.7	48.7	30.1	32.6	43.4	47.0	65.6	69.1
**Ab**/**b**[e]	-	-	-	-	-	-	64.2	69.9	70.4	74.8	87.5	90.7
**Aa**/**c**[e]	-	-	-	-	-	-	24.0	20.8	44.8	47.2	61.6	[f]
**Ba**[d]/**a**[e]	20.3	20.7	48.6	46.2	50.9	57.9	50.9	50.2	58.6	60.1	88.3	82.2
**Ba**/**b**[e]	-	-	-	-	-	-	44.6	39.4	55.1	56.5	76.7	70.7
**Bb**/**c**[e]	-	-	-	-	-	-	58.7	63.0	71.0	73.4	92.6	[f]

[a] Estimated at the DFT2 level. [b] Estimated at the DFT1 level. [c] *Syn*-position (**A**) for Me, NH_2, and NMe_2. [d] *Anti*-position (**B**) for Me, NH_2, and NMe_2. [e] Isomer **a**, **b**, or **c** for symmetrically disubstituted nitriles investigated previously for substituents at *syn*- (**A**) or *anti*-position (**B**) *vis-à-vis* C≡N. [f] Not found.

Table S9. Percentage contents of neutral and protonated isomers {% neutral, % C≡NH$^+$, % C=N(Z)H$^+$, % *syn*-NH$^+$=A(NR$_2$)$_n$, and % *anti*-NH$^+$=A(NR$_2$)$_n$}, and macroscopic basicity parameters (PA$_m$ and GB$_m$ in kJ mol^{-1}) estimated at the DFT2 level for nitriles containing two different large substituents, N=C(NMe$_2$)$_2$ and N=P(NMe$_2$)$_3$ (structures see in Fig. S4 and DFT data see in Table S1).

Isomer	% Neutral	% C≡NH$^+$	% C=N(Z)H$^+$	% NH$^+$=A(NR$_2$)$_n$ *syn*	% NH$^+$=A(NR$_2$)$_n$ *anti*	PA$_m$	GB$_m$
**I.11Aaa**	4.09	0.03	-	<0.001	<0.001		
**I.11Aba**	2.01	40.10	-	0.00$_5$	<<0.001		
**I.11Aab**	1.46	0.10	-	<<0.001	0.00$_4$		
**I.11Abb**	a	0.00$_6$	-	a	a		
**I.11Baa**	9.23	0.54	-	1.44	0.00$_2$	1055.1	1024.4
**I.11Bba**	0.03	30.44	-	0.01	<<0.001		
**I.11Bab**	83.18	27.30	-	0.00$_9$	<0.001		
**I.11Bbb**	a	0.01	-	a	a		
**II.11Aaa**	11.45	0.40	<0.001	<<0.001	<<0.001		
**II.11Aba**	0.83	58.63	0.00$_1$	<<0.001	<<0.001		
**II.11Aab**	0.67	1.02	0.03	<<0.001	<<0.001		
**II.11Abb**	a	<0.001	a	a	a		
**II.11Baa**	83.86	5.68	0.00$_2$	<0.001	<<0.001	1061.7	1034.2
**II.11Bba**	0.57	29.67	<0.001	<<0.001	<<0.001		
**II.11Bab**	2.62	4.46	0.13	<<0.001	<<0.001		
**II.11Bbb**	a	<0.001	a	a	a		

[a] Not Found.
